# Mannosylxylarinols A and B, Two New 3,4‐*Seco*‐ergostane‐type Steroidal Saponins From the Fungus *Xylaria necrophora* SZSS1‐2‐2

**DOI:** 10.1002/cbdv.71800

**Published:** 2026-09-30

**Authors:** Qian Luo, Yu Yang, Rui Xu, Xue Zhao, Hui‐Fang Tan, Yu‐Ying Wei, Er‐Wei Li, Li‐Xia Chen, You‐Zhi Zhang, Chang‐Wei Li

**Affiliations:** ^1^ Academy of Military Medical Sciences Beijing China; ^2^ Wuya College of Innovation Shenyang Pharmaceutical University Shenyang China; ^3^ Institutional Center for Shared Technologies and Facilities Institute of Microbiology Chinese Academy of Sciences Beijing China

**Keywords:** cytotoxicity, mannosylxylarinol, natural products, *Xylaria necrophora*

## Abstract

Two new 3,4‐*seco*‐ergostane‐type steroidal saponins (**1** and **2**) were isolated from the fermentation products of the fungus *Xylaria necrophora* SZSS1‐2‐2, together with four known compounds: 2‐hexylidene‐3‐methylsuccinic acid (**3**), xylcarpin C (**4**), (24*R*)‐22,23‐dihydroxy‐ergosta‐4,6,8(14)‐trien‐3‐one 23‐*β*‐D‐glucopyranoside (**5**), and 5‐carboxymellein (**6**). The structures and absolute configurations of the new compounds were elucidated by comprehensive spectroscopic analyses, electronic circular dichroism (ECD), and GIAO ^1^ ^3^C NMR computational methods, and chemical hydrolysis followed by derivatization to determine the D‑mannose configuration of the sugar moiety. All isolated compounds (**1–6**) were evaluated for their biological activities. Only compound **5** showed selective inhibitory activity, inhibiting BV2 microglial cells with an IC_50_ value of 17.10 ± 4.18 µM. Additionally, none of the compounds exhibited antimicrobial activity against the tested microorganisms.

## Graphical abstract

Two new 3,4‐seco‐ergostane saponins (**1**–**2**) and four known compounds were isolated from *Xylaria necrophora*. Structures were elucidated via spectroscopy, ECD, and computational NMR. Compound **5** selectively inhibited BV2 microglial cells (IC_5__0_ = 17.10 µM), while all compounds showed no antimicrobial activity against tested microorganisms.

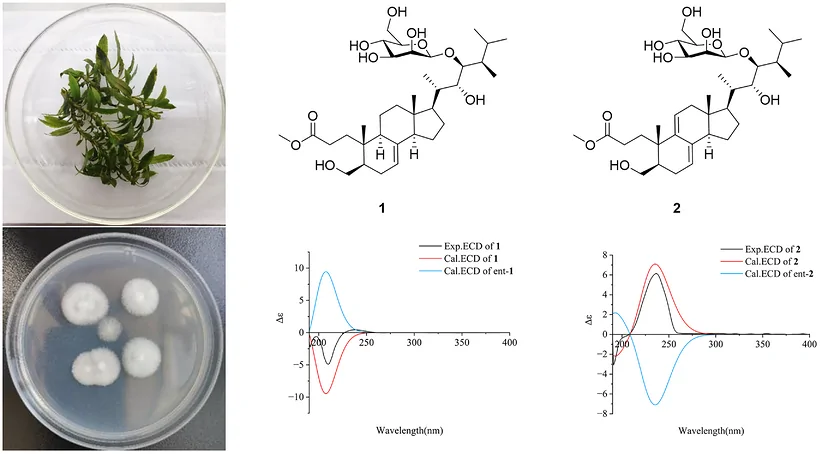

## 1 Introductions

The fungal genus *Xylaria* (family *Xylariaceae*) comprises species that typically grow on decaying wood or plant debris [1]. These fungi are known to produce a broad range of secondary metabolites, including polyketides, terpenoids, alkaloids, and cytochalasins [2]. Many of these secondary metabolites possess antitumor, antimicrobial, antiviral, immunosuppressive, and enzyme inhibitory activities [3, 4, 5, 6, 7, 8, 9, 10]. However, one member that has received limited attention is *Xylaria necrophora*. This species acts as a saprophyte on soybean, cotton, corn, and woody debris and has been linked to taproot decline in soybean [11, 12]. To date, aside from our previous work on the secondary metaboslites of its liquid fermentation, which led to the identification of 11 cytochalasins [13], the secondary metabolome of *X. necrophora* remains largely unexplored.

3,4‐*Seco*‐ergostane‐type steroidal saponins are a class of structurally novel rearranged steroidal natural products derived from fungi. They are derived from the typical ergostane tetracyclic skeleton via oxidative cleavage of the C‐3/C‐4 carbon‐carbon bond, representing an important subtype of *seco*‐ and *abeo*‐ergostane natural products [14]. Structurally, these 3,4‐seco‐ergostane‐type steroidal saponins feature an opened A‐ring and, depending on whether the cleaved termini undergo further cyclization, can exist either as acyclic open‐chain forms or as bridged forms containing an oxygen heterocycle. Since the first representative compound was isolated from the endophytic fungus *Xylaria* sp. in 2018 [15], dozens of structurally diverse 3,4‐*seco*‐ergostane analogues have been identified from endophytic, marine‐derived, and mushroom‐forming fungi [16, 17]. Ergostane‐type steroids represent a major class of steroidal natural products in fungi [16]. These steroids exhibit various pharmacological effects, including antifungal and anti‐phytopathogenic activities [18]. Owing to their novel skeletal rearrangement and potential medicinal value, 3,4‐*seco*‐ergostane‐type steroidal saponins are emerging as a research hotspot in natural product chemistry and drug discovery.

The genus *Xylaria* is well recognized for its capacity to produce structurally diverse secondary metabolites. To explore more novel secondary metabolites from *X. necrophora*, we further isolated two previously undescribed 3,4‐seco‐ergostane‐type steroidal saponins (**1** and **2**) and four other known compounds (**3**‐**6**) (Figure 1).

**FIGURE 1:**
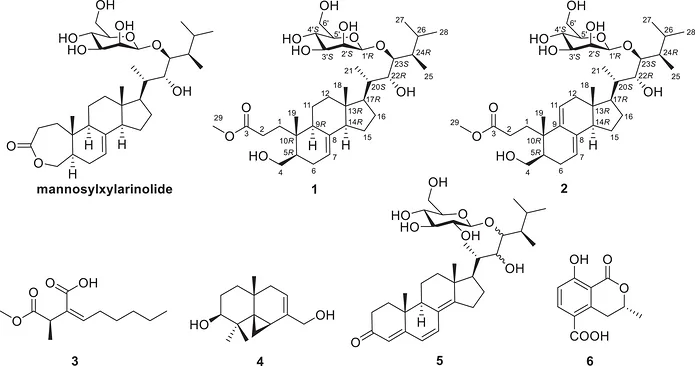
Structures of compounds **1**–**6** and mannosylxylarinolide.

## 2 Results and Discussion

### 2.1 Compounds Isolated From *X. necrophora* SZSS1‐2‐2

Compound **1** was obtained as an amorphous white powder. Its molecular formula was established as C_35_H_60_O_10_ by HR–ESI–MS (Figure S1, *m/z* 663.4081 [M+Na]^+^, calcd for C_35_H_60_O_10_Na, 663.4084, Δ = −0.0003 Da), indicating six degrees of unsaturation. The UV spectrum (Figure S2) showed absorptions at 207 and 250 nm. The IR spectrum (Figure S3) exhibited absorption bands for hydroxy (3377 cm^−^^1^), ester carbonyl (1733 cm^−^^1^), and double bond (1437 cm^−^^1^) functionalities.

The ^1^H and ^1^ ^3^C NMR spectra of **1** (Table 1, Figures S4 and S5) revealed the presence of a sugar moiety, as evidenced by an anomeric proton signal at *δ*_H_ 4.47 (1H, s) and six oxygenated carbon resonances: five oxygenated methines (*δ*_C_ 103.0, 78.1, 75.7, 73.1, 68.3) and one oxygenated methylene (*δ*_C_ 62.7). The aglycone portion displayed an ester carbonyl carbon (*δ*_C_ 176.5), a methoxyl group [*δ*_H_ 3.66 (3H, s, H‐29); *δ*_C_ 52.1], two tertiary methyls [*δ*_H_ 0.83 (3H, s, H‐19), 0.61 (3H, s, H‐18)], four secondary methyls [*δ*_H_ 0.96 (3H, d, *J* = 6.8 Hz, H‐21), *δ*_H_ 0.98 (3H, d, *J* = 6.8 Hz, H‐28), 0.91 (3H, d, *J* = 6.8 Hz, H‐25) and 0.93 (3H, d, *J* = 6.8 Hz, H‐27)], a hydroxymethyl group [*δ*_H_ 3.75 (dd, *J* = 10.4, 4.1 Hz, Ha‐4) and 3.35 (m, Hb‐4); *δ*_C_ 63.3], and a trisubstituted double bond [*δ*_H_ 5.24 (dd, *J* = 5.4, 2.1 Hz, H‐7); *δ*_C_ 140.3 (C‐8), 118.1 (C‐7)].

**TABLE 1:** NMR assignments for **1** and **2** (in CD_3_OD)^a^.

Position	1		2	
	*δ_C_*	*δ*_H_ (*J* in Hz)	*δ*_C_	*δ*_H_ (*J* in Hz)
1	33.1	1.80 (1H, ddd, 14.4, 11.5, 5.4)	35.3	1.83 (1H, ddd, 13.6, 10.7, 5.4)
		1.68 (1H, m, overlapped)		1.65 (1H, ddd, 10.7, 8.3, 5.4)
2	29.0	2.37 (1H, ddd, 15.4, 11.5, 5.4)	30.0	2.30 (1H, ddd, 15.9, 10.7, 5.4)
		2.31 (1H, ddd, 15.4, 11.5, 5.4)		2.18 (1H, m)
3	176.5		176.5	
4	63.3	3.75 (1H, dd, 10.4, 4.1)	62.7	3.63 (1H, m, overlapped)
		3.35 (1H, m)		3.18 (1H, t, 10.1)
5	43.3	1.58 (1H, m, overlapped)	46.4	1.65 (1H, ddd, 10.1, 8.3, 5.4)
6	27.0	2.23 (1H, ddt, 17.7, 5.4, 2.8)	25.4	2.38 (2H, m, overlapped)
		1.88 (1H, m, overlapped)		
7	118.1	5.24 (1H, dd, 5.4, 2.1)	122.7	5.37 (1H, dd, 5.4, 2.0)
8	140.3		136.7	
9	44.6	1.88 (1H, m, overlapped)	139.5	
10	37.3		38.9	
11	23.2	1.47 (1H, m, overlapped)	118.8	5.24 (1H, brs)
		1.32 (1H, m, overlapped)		
12	41.1	2.07 (1H, dt, 12.6, 3.5)	44.0	2.38 (1H, m, overlapped)
		1.32 (1H, m, overlapped)		2.22 (1H, m, overlapped)
13	44.8		44.2	
14	56.7	1.94 (1H, td, 6.6, 2.5)	52.6	2.22 (1H, m, overlapped)
15	23.8	1.58 (1H, m, overlapped)	23.6	1.73 (1H, m, overlapped)
		1.47 (1H, m, overlapped)		1.51 (1H, m, overlapped)
16	28.6	2.01 (1H, m)	29.0	2.06 (1H, m)
		1.38 (1H, m)		1.37 (1H, m)
17	54.2	1.68 (1H, m, overlapped)	54.4	1.73 (1H, m, overlapped)
18	12.4	0.61 (3H, s)	12.7	0.59 (3H, s)
19	18.2	0.83 (3H, s)	22.1	1.08 (3H, s)
20	37.9	1.68 (1H, m, overlapped)	37.8	1.73 (1H, m, overlapped)
21	13.5	0.96 (3H, d, 6.8)	13.0	0.96 (3H, d, 6.5)
22	73.4	3.66 (1H, dd, 8.7, 4.4)	73.4	3.66 (1H, dd, 8.5, 3.8)
23	82.3	3.80 (1H, dd, 8.7, 2.1)	82.4	3.80 (1H, dd, 8.5, 2.2)
24	42.0	1.47 (1H, m, overlapped)	42.0	1.51 (1H, m, overlapped)
25	11.7	0.91 (3H, d, 6.8)	11.7	0.91 (3H, d, 6.5)
26	30.1	1.94 (1H, m, overlapped)	30.1	1.93 (1H, m)
27	21.3	0.93 (3H, d, 6.8)	21.2	0.93 (3H, d, 6.5)
28	22.3	0.98 (3H, d, 6.8)	22.3	0.98 (3H, d, 6.5)
29	52.1	3.66 (3H, s)	52.1	3.63 (3H, s)
1'	103.0	4.47 (1H, brs)	103.0	4.49 (1H, brs)
2'	73.1	3.86 (1H, d, 3.3)	73.1	3.86 (1H, d, 3.2)
3'	75.7	3.39 (1H, dd, 9.6, 3.3)	75.6	3.39 (1H, dd, 9.5, 3.2)
4'	68.3	3.63 (1H, t, 9.6)	68.3	3.63 (1H, m, overlapped)
5'	78.1	3.15 (1H, ddd, 9.6, 4.4, 2.6)	78.0	3.15 (1H, ddd, 9.5, 4.4, 2.6)
6'	62.7	3.83 (1H, dd, 11.5, 2.6)	62.7	3.83 (1H, dd, 11.6, 2.6)
		3.77 (1H, dd, 11.5, 4.4)		3.77 (1H, dd, 11.6, 4.4)

^a^ ^1^H NMR data were measured at 600 MHz and ^1^ ^3^C NMR data at 150 MHz.

A comparison of these NMR data with those of the known analogue mannosylxylarinolide (Figure 1) [15] revealed a close structural resemblance, with a key difference: the seven‐membered lactone ring (C‐3–C‐4) in the latter appeared to be opened in **1**. This was supported by the presence of an additional methoxy singlet [*δ*_H_ 3.66 (3H, s, H‐29); *δ*_C_ 52.1] and a hydroxymethyl group [*δ*_H_ 3.75 (dd, *J* = 10.4, 4.1 Hz, Ha‐4) and 3.35 (m, Hb‐4); *δ*_C_ 63.3] in **1**, suggesting that the seven‐membered lactone ring (C‐3–C‐4) in mannosylxylarinolide had been hydrolyzed and methylated to form a methyl ester, while the other terminus of the cleaved bond became a primary alcohol. This assignment was further corroborated by detailed 2D NMR analysis (Figures 2 and S6–S9). The HMBC correlations from the methoxy protons to C‐3 placed a methyl ester at the opened ring‐A terminus (C‐3). The ^1^H NMR, ^1^H‐^1^H COSY, and MS data confirmed a hydroxymethyl substituent at C‐5. The Δ^7^ double bond was established through the ^1^H‐^1^H COSY and HMBC correlations shown in Figure 2. The sugar unit was attached at C‐23 based on the key HMBC correlation from H‐1′ to C‐23. Other observed COSY and HMBC correlations were consistent with the proposed 3,4‐*seco*‐ergostane framework. Thus, the planar structure of **1** was assigned as shown in Figure 2.

**FIGURE 2:**
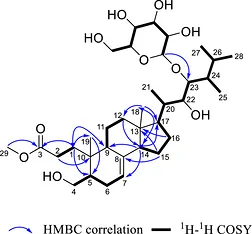
Key HMBC and ^1^H‐^1^H‐COSY data for **1**.

The relative configuration of **1** was established by analysis of the NOESY spectrum (Figure 3). For the sugar unit, the presence of NOESY correlations between H‐1′ and both H‐3′ and H‐5′, along with the absence of such correlations between H‐4′ and H‐3′/H‐5′, revealed that H‐1′, H‐3′, and H‐5′ reside on the same face, while H‐4′ is on the opposite face. This spatial arrangement, coupled with the small coupling constant of the anomeric proton, is in agreement with a *β*‐D‐mannopyranosyl moiety in which H‐1′ occupies an axial position, and the assignment was further corroborated by the close agreement of its NMR data with those of mannosylxylarinolide [15]. In the aglycone, the NOESY cross‐peaks of H‐19/H‐4, H‐19/H*_β_*‐6, H‐18/H*_β_*‐12, H‐18/H‐20, H‐9/H*_α_*‐12, H*_α_*‐12/H‐14, H*_α_*‐12/H‐17 and H‐14/H‐17 revealed that CH_3_‐18, CH_3_‐19 are cofacial (*β*‐oriented), whereas H‐5, H‐9, H‐14 and H‐17 are on the opposite face (*α*‐oriented). For the C‐17 side chain, the ^1^H NMR data and coupling constants of the relevant protons are essentially identical to those of the known mannosylxylarinolide, whose absolute configuration was previously established by x‐ray diffraction (CCDC 1557189) [15]. Collectively, these data indicated that **1** and mannosylxylarinolide share the same relative configurations at C‐5, C‐9, C‐10, C‐13, C‐14, C‐17, C‐20, C‐22, C‐23, and C‐24. To further validate the glycosidic configuration, theoretical ^1^ ^3^C NMR calculations were performed for both *α*‐ and *β*‐D‐mannopyranose anomers (Figure 4, Tables S1 and S2). The calculated data for the *β*‐configuration exhibited significantly better agreement with the experimental values (R^2^ = 0.9977) than those for the *α*‐anomer (R^2^ = 0.994), with a DP4+ probability of 100% (Figure S30) for the *β*‐form, thereby unambiguously confirming the *β*‐D‐mannopyranose assignment.

**FIGURE 3:**
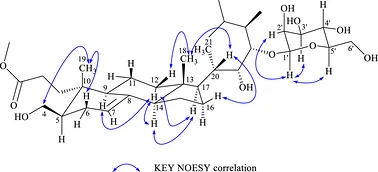
Key NOESY data for **1**.

**FIGURE 4:**
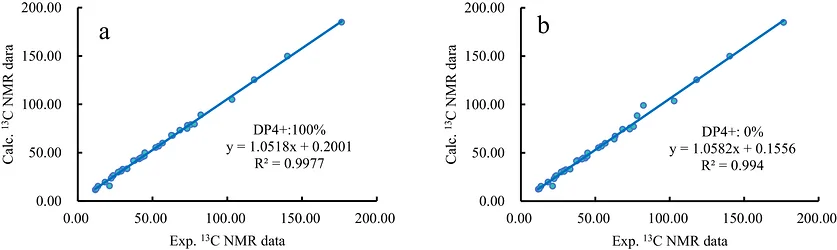
Correlations between experimental and calculated ^1^ ^3^C NMR data for compound **1**: (a) *β*‐D‐mannopyranose configuration; (b) *α*‐D‐mannopyranose configuration.

To confirm the absolute configuration of **1**, the ECD spectra of **1** and *ent*‐**1** were calculated. The experimental curve of **1** matched well with the calculated curve of **1** (Figure 5). Thus, the absolute configuration of **1** was established as drawn in Figure 1. Therefore, the absolute configuration of **1** was established as identical to that of mannosylxylarinolide, and it was named mannosylxylarinol A.

**FIGURE 5:**
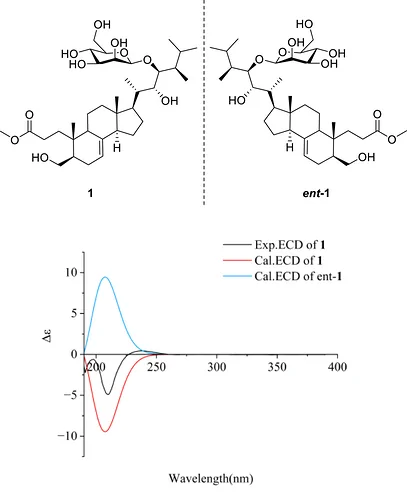
Experimental and calculated ECD spectra of compound **1**.

Compound **2** was also obtained as an amorphous white powder. Its molecular formula was determined as C_35_H_58_O_10_ by HR–ESI–MS (Figure S10, *m/z* 661.3923 [M+Na]^+^, calcd for C_35_H_58_O_10_Na, 661.3928, Δ = −0.0005 Da), indicating one additional degree of unsaturation compared to **1**. The UV spectrum displayed absorptions at 203 and 238 nm (Figure S11). The IR spectrum showed a carbonyl band at 1721 cm^−^^1^ (Figure S12).

The ^1^H and ^1^ ^3^C NMR spectra of **2** (Table 1, Figures S13 and S14) closely resembled those of **1**, with the key difference being that the sp^3^ methine C‐9 and methylene C‐11 in **1** were replaced by two sp^2^ carbons in **2** (*δ*_C_ 139.5 for C‐9 and *δ*_C_ 118.8 for C‐11), and a new olefinic proton signal appeared at *δ*_H_ 5.24 (s, H‐11). Meanwhile, C‐7 and C‐8 retained their original sp^2^ olefinic character. This observation, combined with the molecular formula indicating one additional degree of unsaturation, pointed to the presence of a second double bond in the B ring of **2**. This deduction was firmly supported by 2D NMR data (Figure 6 and S15–S18). The ^1^H‐^1^H COSY correlation of H‐11 with H_2_‐12 and the key HMBC correlations from H‐11 to C‐8 and C‐14, from H‐14 to C‐7 and C‐9, confirmed that the two double bonds were located at Δ^7^ and Δ^9^^(^^1^ ^1^^)^. Other observed COSY and HMBC correlations were fully consistent with the same planar structure as that of **1**. Therefore, **2** was identified as the 9,11‐dehydro analog of **1**, and its planar structure was assigned as depicted in Figure 1.

**FIGURE 6:**
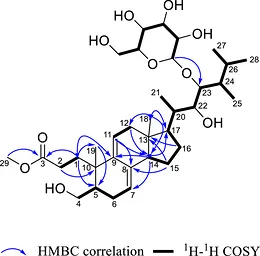
Key HMBC and ^1^H,^1^H‐COSY data for **2**.

The NOESY spectrum of **2** (Figure 7) displayed essential correlations similar to those of **1**, and the diagnostic sugar NOE pattern characteristic of a *β*‐D‐mannopyranose unit. Moreover, the ^1^H NMR data and coupling constants of the side‐chain protons were nearly identical to those of **1** and mannosylxylarinolide [15], indicating their same relative stereochemistry. The *β*‐D‐mannopyranose configuration of **2** was further confirmed by theoretical ^1^ ^3^C NMR calculations (Figure 8, Tables S3 and S4), which gave a DP4+ probability of 100% for the *β*‐form (Figure S34), consistent with the assignment for **1** as described above.

**FIGURE 7:**
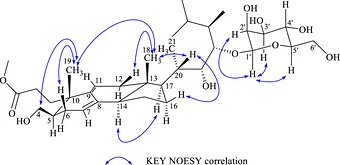
Key NOESY data for **2**.

**FIGURE 8:**
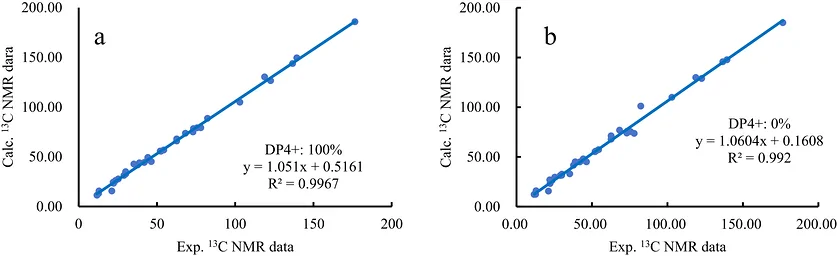
Correlations between experimental and calculated ^1^ ^3^C NMR data for compound 2: (a) *β*‐D‐mannopyranose configuration; (b) *α*‐D‐mannopyranose configuration.

To confirm the absolute configuration of **2**, calculated ECD (Figure 9) was also performed. All results confirmed that the absolute configuration of **2** is as depicted in Figure 1. Compound **2** was named mannosylxylarinol B.

**FIGURE 9:**
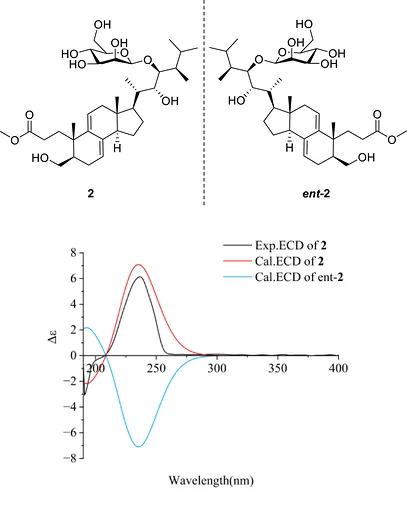
Experimental and calculated ECD spectra of compound **2**.

To determine the D/L configuration of the mannose moiety, compound **2** was subjected to hydrolysis, and the liberated mannose was derivatized. The resulting derivative was then analyzed by HPLC under the same conditions as those used for the derivatives of D‑mannose and L‑mannose standards. The retention time of the sample derivative matched that of the D‑mannose standard (Figure S40), indicating that the sugar unit in compound **2** is D‑mannose. Furthermore, since compounds **1** and **2** share the same biosynthetic origin, this assignment could be further corroborated for compound **1** as well. These findings are in line with the results described above.

Compounds **3–6** were identified as 2‐hexylidene‐3‐methylsuccinic acid (**3**) [19], xylcarpin C (**4**) [20], (24*R*)‐22,23‐dihydroxyergosta‐4,6,8(14)‐trien‐3‐one 23‐*β*‐D‐glucopyranoside (**5**) [21] and 5‐carboxymellein (**6**) [22] by comparing their spectroscopic data (Figures S19–S26), MS and [*α*]_D_ data with literature values.

### 2.2 Cytotoxic and Antimicrobial Activity

The cytotoxic and antimicrobial activities of compounds **1–6** were evaluated. Cytotoxicity was assessed against murine microglial (BV2), human breast cancer (MCF‐7), human lung adenocarcinoma (A549), and human neuroblastoma (SH‐SY5Y) cells using the MTT assay (Table 2). To ensure the reliability of the assay, 5‐fluorouracil was used as a positive control. Among all tested compounds (**1–6**), only compound **5** exhibited selective inhibitory activity against BV2 cells, with an IC_50_ value of 17.10 ± 4.18 µM. The remaining compounds (**1–4** and **6**) were inactive across all cell lines tested, with IC_50_ values exceeding 100 µM. Antimicrobial activity was evaluated using the broth microdilution method. None of the compounds exhibited antimicrobial activity against *Candida albicans*, *Staphylococcus aureus*, or *Escherichia coli* at concentrations up to 256 *µ*g/mL.

**TABLE 2:** Cytotoxicity assay results (IC_50_) of compounds **1–6** (*µ*M, *n* = 3).

Comp.	BV2	MCF‐7	A549	SH‐SY5Y
**1**	>100	>100	>100	>100
**2**	>100	>100	>100	>100
**3**	>100	>100	>100	>100
**4**	>100	>100	>100	>100
**5**	17.10 ± 4.18	>100	>100	>100
**6**	>100	>100	>100	>100
**5‐Fluorouracil^1^**	0.42 ± 0.36	4.21 ± 2.34	37.38 ± 2.47	7.81 ± 0.40

*Note*: ^1^5‐Fluorouracil was used as the positive control.

## 3 Conclusion

In summary, we isolated and characterized six compounds from the fungus *X. necrophora* SZSS1‐2‐2. Their structures were determined by comprehensive spectroscopic analysis and theoretical chemical calculations. The absolute configurations of **1** and **2** were established by comparing experimental and calculated ECD spectra. All compounds were assessed for their biological activities. Onlycompound **5** exhibited inhibitory activity against BV2 cells, with an IC_50_ value of 17.10 ± 4.18 µM, while none of the compounds showed significant cytotoxicity against MCF‐7, A549, or SH‐SY5Y cell lines at concentrations up to 100 µM. Additionally, no significant antimicrobial activity was observed for any of the compounds against *C. albicans*, *S. aureus*, or *E. coli* in the preliminary screening.

This study expands the chemical diversity of 3,4‐*seco*‐ergostane derivatives. Although compounds **1** and **2** showed no cytotoxic or antimicrobial activity in this preliminary screening, their unique 3,4‐seco‐ergostane skeleton still merits further investigation into other pharmacological properties. The bioactivity results suggest that compound **5** warrants further biological investigation due to its selective activity against BV2 cells. The isolation of these 3,4‐*seco*‐ergostane derivatives from *X. necrophora* expands the known chemical diversity of this fungal species.

## 4 Experimental

### 4.1 General

Fermentation was performed using ZHWY‐2102 rotary shakers (Shanghai Zhi Cheng Analyzing Instrument Manufactory Co. Ltd., Shanghai, China). Chromatographic separations were performed using a Waters HPLC system (600 controller, 600 pump, and a 2996 photodiode array detector for analytical HPLC or a 2998 detector for preparative HPLC; Waters, Milford, MA, USA) and on a Pilot100 system (SP0530 high‐pressure pump and UV1001 UV detector; Sanotac Scientific Instrument Co., Ltd., Shanghai, China). The columns utilized were Venusil XBP C_4_ (4.6 × 250, 10 × 250, and 21.2 × 250 mm, 5 µm; Agela Technologies, Tianjin, China), YMC‐Pack ODS‐A (4.6 × 250 and 20 × 250 mm, 5 µm; YMC Co., Ltd, Shanghai, China), and Capcell pak C_18_ MGII and Capcell pak C_18_ AQ (each available in 4.6 × 250, 10 × 250, and 20 × 250 mm, 5 µm; Shiseido Co., Ltd., Tokyo, Japan). High‐resolution and low‐resolution ESI–MS data were acquired on an Agilent 1260‐G6230 A LC/MS spectrometer (Agilent Technologies, Santa Clara, USA). NMR spectra (^1^H at 600 MHz, ^1^ ^3^C at 150 MHz) were recorded on a Bruker Avance 600 spectrometer (Bruker, Karlsruhe, Germany). UV spectra were measured on a Genesys10S UV/VIS spectrophotometer, and IR spectra were recorded on a Nicolet IS5 FT–IR spectrometer (Thermo–Fisher Scientific, New York, USA). ECD spectra were recorded on a MOS 450 AF–CD spectrometer (Bio‐Logic, France), and optical rotations were measured using an MCP 4100 spectropolarimeter (Anton Paar GmbH, Graz, Austria). Cell morphology was observed using an AE31 EF–INV inverted microscope (Motic China Group Co., Ltd., Shanghai, China). MTT assays were read on a SpectraMax ABS Plus microplate reader (Molecular Devices, USA).

### 4.2 Cell Lines, Bacteria and Reagents

The following cell lines were kindly provided by Professors Zhiyong Xiao, Yuan Luo, and Jun Yang (Academy of Military Medical Sciences): MCF‐7 human breast cancer cells (RRID: CVCL_0031), SH‐SY5Y human neuroblastoma cells (RRID: CVCL_0019), A549 human lung carcinoma cells (RRID: CVCL_0023), and BV2 murine microglial cells (RRID: CVCL_0182). Microbial strains (*C. albicans* ATCC 10231, *S. aureus* ATCC 29213, and *E. coli* ATCC 25922) were obtained from Prof. Kui Zhu at China Agricultural University (Beijing, China). For cell culture, RPMI‐1640 medium (lot 6125115/2351416) was produced by ThermoFisher Biochemical Products (Beijing) Co., Ltd., and DMEM (lot 8122580) was purchased from Sigma‐Aldrich (St. Louis, MO, USA); fetal bovine serum (lot 25010702) was sourced from Zhejiang Tianhang Biotechnology Co., Ltd. (Zhejiang, China). MTT (lot 0793) was also purchased from Sigma‐Aldrich. Fluorouracil (5‐FU, lot 5402) was supplied by Aladdin Chemistry Co., Ltd. (Shanghai, China). The antibiotics benzylpenicillin potassium (lot 20190905) and streptomycin sulfate (lot 20190902) were obtained from Sichuan Jishanzhijia Pharmaceutical Co., Ltd. (Sichuan, China), while amphotericin B (lot 620378) was purchased from Sigma–Aldrich. For microbial experiments, brain‐heart infusion broth (lot 20210813), Mueller–Hinton broth (lot 20200806), glucose (lot 20220512), yeast extract (lot 20201118), and peptone (lot 20210729) were all purchased from Beijing Aoboxing Biotechnology Co., Ltd.

### 4.3 Fungal Material

The strain *X. necrophora* SZSS1‐2‐2 was identified by Engineer Bangbang Yang (Shiyanjia Lab, www.shiyanjia.com). It was isolated from the plant *Huperzia serrata* (voucher Nos. BIPT‐P‐2023001, BIPT‐P‐2023002, BIPT‐P‐2023003, and BIPT‐P‐2023004) [23]. Its ITS sequence was deposited in GenBank under accession number PQ774807, and the strain was preserved at the Academy of Military Medical Sciences, Beijing 100850, China.

### 4.4 Fermentation, Extraction and Isolation

*X. necrophora* SZSS1‐2‐2 was grown on potato dextrose agar (PDA) at 28°C to obtain a crude spore suspension. This suspension was used to inoculate flasks containing a liquid medium (200 g potato extract, 20 g dextrose, and 1 mL of 95% ethanol per liter of water). Based on previous reports that ethanol supplementation can enhance the diversity of fungal secondary metabolites [24], 1 mL of 95% ethanol per liter of medium was added to the culture medium during the preliminary screening process to promote the production of more diversified metabolites. A total of 38.25 L of liquid medium was used for the fermentation. Fermentation was conducted at 28°C with shaking for 14 days. The broth was subsequently extracted with acetone and ethyl acetate (EtOAc) at room temperature, yielding 28.9 g of crude extract.

The extract was subjected to silica gel column chromatography (7.5 × 25.5 cm) eluted with a gradient of petroleum ether/CH_2_Cl_2_ (1:0 to 0:1) and then CH_2_Cl_2_/MeOH (1:0 to 0:1), yielding nine fractions (Fr1–Fr9). Fr3 (7.57 g) was subjected to ODS column chromatography eluted with a gradient of MeOH/H_2_O (20:80 to 100:0) and then CH_2_Cl_2_/MeOH (1:1), yielding eleven fractions (Fr3‐1–Fr3‐11). Fr3‐2 (0.60 g, eluted by MeOH/H_2_O 2:3) was separated on preparative HPLC (YMC‐Pack ODS‐A, 8.0 mL/min, MeCN/H_2_O 25:75) to give Fr3‐2‐2 (139 mg, *t_R_* = 45–55 min), from which compound **6** (4 mg) was obtained by recrystallization from MeOH‐H_2_O (9:1). Fr3‐3 (0.54 g, eluted by MeOH/H_2_O 3:2) was separated on preparative HPLC (YMC–Pack ODS‐A, 8.0 mL/min, MeCN/H_2_O 28:72) to give Fr3‐3‐3 (15 mg, *t_R_* = 105 min), which was further purified on a Capcell pak C18 MGII column (8.0 mL/min, MeCN/H_2_O 28:72) to yield **4** (2 mg, *t_R_* = 130–135 min). Fr3‐6 (0.47 g, eluted by MeOH/H_2_O 8:2) was separated on preparative HPLC (YMC–Pack ODS‐A, 8.0 mL/min, MeOH/H_2_O 60:40) to give Fr3‐6‐8 (31 mg, *t_R_* = 80–84 min). Purification of Fr3‐6‐8 on a Venusil XBP C_4_ column (2.5 mL/min, MeCN/H_2_O 34:66) yielded **3** (15 mg, *t_R_* = 145–155 min). Fr5 (8.40 g) was subjected to ODS column chromatography, eluted with a gradient of H_2_O to MeOH/H_2_O (20:80 to 100:0) and then CH_2_Cl_2_/MeOH (1:1), to give six fractions (Fr5‐1–Fr5‐6). Fr5‐4 (0.48 g, eluted by MeOH/H_2_O 4:6 to 6:4) was separated on preparative HPLC (Capcell pak C_18_ AQ, 8.0 mL/min, MeCN/H_2_O 34:64) to give Fr5‐4‐4 (34 mg, *t_R_* = 98–102 min) and Fr5‐4‐5 (18 mg, *t_R_* = 130–140 min). Purification of Fr5‐4‐4 on Capcell pak C_18_ AQ column (8.0 mL/min, MeOH/H_2_O 32:68) afforded **2** (6 mg, *t_R_* = 105–110 min). Purification of Fr5‐4‐5 on a Venusil XBP C_4_ column (8.0 mL/min, MeOH/H_2_O 57:43) yielded **1** (4 mg, *t_R_* = 155–165 min). Fr5‐5 (0.40 g, eluted by MeOH/H_2_O 6:4) was separated on preparative HPLC (Capcell pak C_18_ MGII, 8.0 mL/min, MeOH/H_2_O 62:38) to give Fr5‐5‐5 (59 mg, *t_R_* = 100–110 min), which was purified on a Capcell pak C_18_ AQ column (8.0 mL/min, MeOH/H_2_O 61:39) to yield **5** (41 mg, *t_R_* = 150–155 min). Representative preparative HPLC chromatograms for compounds **1–5** are provided in Figures S35–S39.

1. Mannosylxylarinol A (**1**): Colorless powder (MeOH), [*α*]^25^_D_ −7.11 (c = 0.100, MeOH); UV (MeOH) *λ*_max_ (log *ɛ*): 207 (2.67), 250 (1.83); ECD (MeOH) *λ*_max_ (*Δɛ*): 200 (‐0.98) nm, 210 (‐4.91) nm, 238 (0.44) nm, 285 (−12.00) nm; IR (film) *ν*_max_: 3377, 2950, 2871, 1733, 1437, 1381, 1315, 1256, 1175, 1071, 1027, 987 cm^−1^; ^1^H and ^13^C NMR data see Table 1; positive HR–ESI–MS *m/z* 663.4081 [M+Na]^+^ (calcd. for C_35_H_60_O_10_Na^+^, 663.4084, Δ = −0.0003 Da).
2. Mannosylxylarinol B (**2**): Colorless powder (MeOH), [*α*]^25^_D_ +28.13 (c = 0.100, MeOH); UV (MeOH) *λ*_max_ (log *ɛ*): 203 (2.37), 238 (1.83); ECD (MeOH) *λ*_max_ (*Δɛ*): 191 (−3.07) nm, 236 (6.13) nm; IR (film) *ν*_max_: 3378, 2950, 2871, 1721, 1440, 1378, 1262, 1176, 1071, 1026, 985 cm^−1^; ^1^H and ^13^C NMR data see Table 1; positive HR–ESI–MS *m/z* 661.3923 [M+Na]^+^ (calcd. for C_35_H_58_O_10_Na^+^, 661.3928, Δ = −0.0005 Da).
3. Compound **3**: White powder (MeOH), [*α*]^25^_D_ −34.29 (c = 0.102, CHCl_3_); positive ESI–MS *m/z* 251.1 [M+Na]^+^.
4. Compound **4**: White powder (MeOH), [*α*]^25^_D_ +87.63 (c = 0.101, Me_2_CO); positive ESI–MS *m/z* 237.1 [M+H]^+^.
5. Compound **5**: White powder (MeOH), [*α*]^25^_D_ −236.33 (c = 0.100, MeOH); positive ESI–MS *m/z* 611.3 [M+Na]^+^.
6. Compound **6**: White powder (DMSO), [*α*]^25^_D_ −224.06 (c = 0.100, Me_2_CO); positive ESI–MS *m/z* 223.0 [M+H]^+^.

### 4.5 Hydrolysis of Compound **2** and Determination of the Mannose Configuration

The hydrolysis of compound **2** and determination of the mannose configuration were performed according to the procedure reported in the literature [25]. Standard D‐mannose (5.0 mg) and L‐cysteine methyl ester hydrochloride (5.0 mg) were dissolved in 1.0 mL of pyridine and heated at 60°C. Then, 0.2 mL of *o*‐tolylisothiocyanate was added to the mixture, and the reaction was allowed to proceed for 1 h to obtain the derivatized products. The same procedure was applied to standard L‐mannose to prepare its corresponding derivatives. Compound **2** (0.5 mg) was dissolved in 300 µL of acetone, and then 6 N HCl (700 µL) was added to the reaction vial. The mixture was heated at 100°C for 24 h. The reaction was quenched by the addition of 3.0 mL of H_2_O, and the aglycone moiety was removed by extraction twice with 2.0 mL of CHCl_3_. After evaporation of the aqueous layer to dryness, the residue was treated with L‐cysteine methyl ester hydrochloride (1.0 mg) in 1100 µL of pyridine at 60°C for 1 h. Finally, *o*‐tolylisothiocyanate (100 µL) was added, and the mixture was heated at 60°C for an additional 1 h. The reaction mixture was then analyzed by analytical HPLC on a CAPCELL PAK AQ C_18_ column (4.6 × 250 mm) with 25% CH_3_CN in H_2_O containing 0.1% TFA as the mobile phase at a flow rate of 0.6 mL/min and a column temperature of 35°C, with detection at 250 nm.

### 4.6 Cytotoxicity Assay

Cell viability was assessed by the MTT assay according to our previously described procedure [26]. Four cell lines (MCF‐7, SH‐SY5Y, BV2, and A549) in the exponential growth phase were harvested and resuspended in fresh RPMI‐1640 medium containing 10% fetal bovine serum and 100 µg/mL penicillin‐streptomycin. The cell suspensions were seeded into 96‐well plates. Stock solutions of test compounds **1–6** were prepared in methanol at a concentration of 4 mM. 5‐Fluorouracil (5‐Fu) was dissolved in dimethyl sulfoxide (DMSO) to give a 10.0 mg/mL stock solution and served as the positive control, while methanol was used as the blank control. After the addition of test samples or controls, the plates were incubated at 37°C for 48 h.

All experiments were performed in triplicate, and the absorbance was read at 570 nm on a SpectraMax ABS Plus microplate reader. The inhibition rate (IR%) was calculated as: (IR)% = (*OD*_control_‐*OD*_sample_)/*OD*_control_ × 100%. IC_50_ values were obtained by nonlinear regression of the concentration–response curves (percent inhibition vs. compound molar concentration) fitted with a four‑parameter logistic model. The IC_50_ calculations were performed using Excel software (Microsoft Corporation, Redmond, WA, USA), and the dose–response curves were generated with GraphPad Prism (San Diego, CA, USA). All data are presented as the mean ± standard deviation (SD) of three independent replicates. No pre‐processing procedures such as data transformation or outlier exclusion were applied. All data are presented as the mean ± standard deviation (SD) of three independent replicates (*n* = 3). Since the purpose of this study was to determine IC_50_ values for preliminary activity screening, no statistical hypothesis tests were performed to compare between groups, and therefore no *p* values are reported.

### 4.7 Antimicrobial Assay

The MICs of compounds **1–6** against *C. albicans* ATCC 10231 were measured by the microbroth dilution method according to CLSI M60‐Ed2 [27], while those against *E. coli* ATCC 25922 and *S. aureus* ATCC 29213 were determined following CLSI M100‐Ed32 [28]. Stock solutions of the compounds were serially diluted two‐fold to give a concentration range of 256 to 8 µg/mL.

*C. albicans* was grown on YPD agar plates at 28°C for 24 h. The bacterial strains were cultured in brain heart infusion (BHI) broth at 37°C with shaking until the logarithmic growth phase was reached. All microbial suspensions were adjusted to a 0.5 McFarland standard and then further diluted to obtain the final inoculum densities: approximately 2.5 × 10^3^ CFU/mL for *C. albicans* in RPMI 1640 medium and 1.0 × 10^6^ CFU/mL for bacteria in Mueller–Hinton broth (MHB). After inoculation, the microtiter plates were mixed gently and incubated at 37°C for 18 h (bacteria) or at 35°C for 24 h (yeast). The MIC was defined as the lowest concentration that completely prevented visible growth. All MIC determinations were performed in duplicate, and the results were consistently reproducible. No statistical analyses were applied to the antimicrobial data, as the endpoint (MIC) is a discrete categorical value determined by visual inspection. Amphotericin B and benzylpenicillin potassium served as the positive controls for the yeast and bacterial assays, respectively.

### 4.8 Calculation of ^13^C NMR Data of 1–2 and ECD of Compounds **1**–**2**

Conformational searches were initially carried out with Spartan'14 (Version 1.1.4). For compounds **1** and **2** (Figures S27 and S31), all conformers (Figures S28, S29, S32, and S33) with a Boltzmann population above 1% were retained and further optimized at the B3LYP/6‐311+G (2d, p) level. NMR chemical shifts of **1** (with *β*‐D‐ mannosyl group and *α*‐D‐ mannosyl group) and **2** (with *β*‐D‐ mannosyl group and *α*‐D‐ mannosyl group) were then predicted by the GIAO/mPW1PW91/6‐311+G (d, p) method. ECD spectra of **1**, ***ent*‐1**, **2**, and ***ent*‐2** were simulated at the B3LYP/6‐311+G (2d, p) level. Optical rotations were computed at the APFD/6‐311+G (2d, p) level. All quantum chemical calculations were performed using the Gaussian 16 A03 package [29].

## Author Contributions

Isolation: L. Q.; bioactivity evaluation: Z. X.; structure elucidation: L. Q., X. R., T. H.‐F., W. Y.‐Y.; computational calculation: Y. Y., L. C.‐W. and L. E.‐W.; writing – original draft preparation, L. Q.; supervision, C. L.‐X., Z. Y.‐Z. and L. C.‐W.; writing – review and editing, project administration, L. C.‐W.

## Funding

The authors have nothing to report.

## Conflicts of Interest

The authors declare no conflicts of interest.

## Supporting information




**Supporting File 1**: cbdv71800‐sup‐0001‐SuppMat.pdf.

## Data Availability

The data that support the findings of this study are available from the corresponding author upon reasonable request.

## References

[1] M. L. Macías‐Rubalcava and R. E. Sánchez‐Fernández , “Secondary Metabolites of Endophytic *Xylaria* Species With Potential Applications in Medicine and Agriculture,” World Journal of Microbiology and Biotechnology 33 (2017): 15. 10.1007/s11274-016-2174-5 27896581

[2] K. Becker and M. Stadler , “Recent Progress in Biodiversity Research on the *Xylariales* and Their Secondary Metabolism,” Journal of Antibiotics 74 (2021): 1–23. 10.1038/s41429-020-00376-0 PMC7732752 33097836

[3] W. Chen , M. Yu , S. Chen , et al., “Structures and Biological Activities of Secondary Metabolites From *Xylaria spp* ,” Journal of Fungi 10 (2024): 190. 10.3390/jof10030190 PMC10971283 38535199

[4] A. Schüffler , O. Sterner , and H. Anke , “Cytotoxic α‑Pyrones From *Xylaria Hypoxylon* ,” Zeitschrift für Naturforschung C 62 (2007): 169–172. 10.1515/znc-2007-3-403 17542480

[5] S. T. Deyrup , J. B. Gloer , K. O'Donnell , and D. T. Wicklow , “Kolokosides A–D: Triterpenoid Glycosides From a Hawaiian Isolate of *Xylaria sp* ,” Journal of Natural Products 70 (2007): 378–382. 10.1021/np060546k 17284074

[6] C. W. Lei , Z. Q. Yang , Y. P. Zeng , et al., “Xylastriasan A, a New Cytochalasin From the Fungus *Xylaria striata* ,” Natural Product Research 32 (2018): 7–13. 10.1080/14786419.2017.1324959 28486854

[7] W. Zeng , K. Habib , X. Zhou , et al., “Morphology and Multigene Phylogeny Reveal Four New *Xylaria* (Xylariales, Xylariaceae) Species from Karst Region in China,” MycoKeys 108 (2024): 169–196. 10.3897/mycokeys.108.130565 PMC11391122 39268505

[8] Q. R. Li , K. Habib , Y. P. Wu , et al., “Revisiting Xylaria Diversity in Southern China: Descriptions of 40 New Species,” Journal of Systematics and Evolution 62 (2024): 1255–1330.

[9] F. Song , S. H. Wu , Y. Z. Zhai , Q. C. Xuan , and T. Wang , “Secondary Metabolites from the Genus *Xylaria* and Their Bioactivities,” Chem Biodiversity 11 (2014): 673–694. 10.1002/cbdv.201200286 24827679

[10] S. K. Deshmukh , K. R. Sridhar , S. Saxena , and M. K. Gupta , Recent Advances in the Discovery of Bioactive Metabolites From Xylaria Hill Ex Schrank (Springer, 2022).

[11] T. Garcia‐Aroca , P. P. Price , M. Tomaso‐Peterson , et al., “ *Xylaria necrophora*, sp. nov., an Emerging Root‑Associated Pathogen Responsible for Taproot Decline of Soybean in the Southern United States,” Mycologia 113 (2021): 326–347. 10.1080/00275514.2020.1846965 33555993

[12] J. D. Rogers , “The Xylariaceae: Systematic, Biological and Evolutionary Aspects,” Mycologia 71 (1979): 1–42.

[13] Q. Luo , C. Sun , X. Zhao , et al., “Cytochalasinols A and B, Two New Cytochalasins From the Fungus *Xylaria necrophora* SZSS1‐2‐2,” Chemistry & Biodiversity 23 (2026): e03213. 10.1002/cbdv.202503213 41289054

[14] F. L. Duecker , F. Reu , and P. Heretsch , “Rearranged Ergostane‐Type Natural Products: Chemistry, Biology, and Medicinal Aspects,” Organic & Biomolecular Chemistry 17 (2019): 1624–1633. 10.1039/c8ob02325e 30357222

[15] G. H. Tang , Y. H. Zhang , Y. C. Gu , S. Y. Li , Y. B. Wu , and Y. Li , “Mannosylxylarinolide, a New 3,4‐ *Seco*‐Ergostane‐Type Steroidal Saponin Featuring a *β*‐d‐Mannose from the Endophytic Fungus *Xylaria* sp,” Journal of Asian Natural Products Research 22 (2020): 397–403. 10.1080/10286020.2018.1563075 30693785

[16] V. N. Zhabinskii , P. Drasar , and V. A. Khripach , “Structure and Biological Activity of Ergostane‐Type Steroids From Fungi,” Molecules 27 (2022): 2103. 10.3390/molecules27072103 PMC9000798 35408501

[17] T. Kikuchi , “Ergostane‐Type Steroids from Mushrooms of *Pleurotus* Genus,” Journal of Natural Medicines 79 (2025): 289–302. 10.1007/s11418-024-01872-5 PMC11880049 39825075

[18] J. F. Wang , G. Q. Li , H. M. Wang , X. Yin , X. L. Yang , and Y. H. Zhang , “Polyhydroxylated Sesquiterpenes and Ergostane Glycosides Produced by the Endophytic Fungus *Xylaria* sp. from *Azadirachta indica* ,” Phytochemistry 199 (2022): 113188. 10.1016/j.phytochem.2022.113188 35421432

[19] S. Klaiklay , V. Rukachaisirikul , Y. Sukpondma , S. Phongpaichit , J. Buatong , and B. Bussaban , “Metabolites From the Mangrove‐Derived Fungus *Xylaria cubensis* PSU‐MA34,” Archives of Pharmacal Research 35 (2012): 1127–1131. 10.1007/s12272-012-0701-y 22864733

[20] X. Yin , T. Feng , Z. H. Li , et al., “Chemical Investigation on the Cultures of the Fungus *Xylaria Carpophila* ,” Natural Products and Bioprospecting 1 (2011): 75–80.

[21] W. Li , X. Q. Yang , Y. B. Yang , et al., “A Novel Steroid Derivative and a New Steroidal Saponin from Endophytic Fungus *Xylaria* *sp* ,” Natural Products Communications 12 (2017): 901–904.

[22] Z. L. Xu , N. Zheng , S. M. Cao , et al., “Secondary Metabolites From the Endophytic Fungus *Stemphylium lycopersici* and Their Antibacterial Activities,” Chemistry of Natural Compounds 56 (2020): 1162–1165.

[23] Y. S. Kang , H. W. Zhu , X. Zhao , et al., “Diversity of Endophytic Fungi Isolated from *Huperzia serrata* and Research on the Secondary Metabolites of *Penicillium panissanguineum* SZSS 4‐2‐2,” Chem Biodiversity 22 (2025): e202500701. 10.1002/cbdv.202500701 40098595

[24] X. M. Zhao , Z. Q. Wang , S. H. Shu , et al., “Ethanol and Methanol Can Improve Huperzine A Production From Endophytic *Colletotrichum Gloeosporioides* ES026,” PLoS ONE 8 (2013): e61777. 10.1371/journal.pone.0061777 PMC3629160 23613930

[25] T. Tanaka , T. Nakashima , T. Ueda , K. Tomi , and I. Kouno , “Facile Discrimination of Aldose Enantiomers by Reversed‐Phase HPLC,” Chemical & Pharmaceutical Bulletin 55 (2007): 899–901. 10.1248/cpb.55.899 17541189

[26] C. J. Wu , C. W. Li , and C. B. Cui , “Seven New and Two Known Lipopeptides as Well as Five Known Polyketides: The Activated Production of Silent Metabolites in a Marine‐Derived Fungus by Chemical Mutagenesis Strategy Using Diethyl Sulphate,” Marine Drugs 12 (2014): 1815–1838. 10.3390/md12041815 PMC4012460 24686557

[27] Clinical and Laboratory Standards Institute (CLSI) , Performance Standards for Antifungal Susceptibility Testing of Yeasts; Document M60 Standard‐Second Edition, (Clinical and Laboratory Standards Institute, 2020).

[28] Clinical and Laboratory Standards Institute (CLSI) , Performance Standards for Antimicrobial Susceptibility Testing of Yeasts; Document M100 Standard‐Thirty‐two Edition, (Clinical and Laboratory Standards Institute, 2022).

[29] M. J. Frisch , G. W. Trucks , H. B. Schlegel , et al., Gaussian 16, Revision A.03, (Gaussian, Inc., 2016).

