# Supplementary material for: Mannosylxylarinols A and B, Two New 3,4‐Seco‐ergostane‐type Steroidal Saponins From the Fungus Xylaria necrophora SZSS1‐2‐2
Source: Chem Biodivers. 2026 Sep 30;23(10):e71800. doi: 10.1002/cbdv.71800 (PMC13626255; doi:10.1002/cbdv.71800)
Supplement: Supplementary file 1 [file CBDV-23-e71800-s001.pdf]

Contents of Supporting information

Figure S1. HRESIMS spectrum of mannosylxylarinol A (**1**) 3
Figure S2. UV spectrum of mannosylxylarinol A (**1**) 3
Figure S3. IR spectrum of mannosylxylarinol A (**1**) 3
Figure S4. ^{1}H NMR (600 MHz) spectrum of mannosylxylarinol A (**1**) in methanol-d_4 4
Figure S4-1. Enlarged ^{1}H NMR (600 MHz) spectrum of mannosylxylarinol A (**1**) in methanol-d_4 4
Figure S4-2. Enlarged ^{1}H NMR (600 MHz) spectrum of mannosylxylarinol A (**1**) in methanol-d_4 5
Figure S5. ^{13}C NMR and DEPT 135 (150 MHz) spectra of mannosylxylarinol A (**1**) in methanol-d_4 5
Figure S6. ^{1}H−^{1}H COSY spectrum of mannosylxylarinol A (**1**) in methanol-d_4 6
Figure S7. HSQC spectrum of mannosylxylarinol A (**1**) in methanol-d_4 6
Figure S8. HMBC spectrum of mannosylxylarinol A (**1**) in methanol-d_4 7
Figure S9. NOESY spectrum of mannosylxylarinol A (**1**) in methanol-d_4 7
Figure S10. HRESIMS spectrum of mannosylxylarinol B (**2**) 8
Figure S11. UV spectrum of mannosylxylarinol B (**2**) 8
Figure S12. IR spectrum of mannosylxylarinol B (**2**) 8
Figure S13. ^{1}H NMR (600 MHz) spectrum of mannosylxylarinol B (**2**) in methanol-d_4 9
Figure S13-1. Enlarged ^{1}H NMR (600 MHz) spectrum of mannosylxylarinol B (**2**) in methanol-d_4 9
Figure S13-2. Enlarged ^{1}H NMR (600 MHz) spectrum of mannosylxylarinol B (**2**) in methanol-d_4 10
Figure S14. ^{13}C NMR and DEPT 135 (150 MHz) spectra of mannosylxylarinol B (**2**) in methanol-d_4 10
Figure S15. ^{1}H−^{1}H COSY spectrum of mannosylxylarinol B (**2**) in methanol-d_4 11
Figure S16. HSQC spectrum of mannosylxylarinol B (**2**) in methanol-d_4 11
Figure S17. HMBC spectrum of mannosylxylarinol B (**2**) in methanol-d_4 12
Figure S18. NOESY spectrum of mannosylxylarinol B (**2**) in methanol-d_4 12
Figure S19. ^{1}H NMR (600 MHz) spectrum of 2-hexylidene-3-methylsuccinic acid (**3**) in methanol-d_4 13
Figure S20. ^{13}C NMR (150 MHz) spectra of 2-hexylidene-3-methylsuccinic acid (**3**) in methanol-d_4 . 13
Figure S21. ^{1}H NMR (600 MHz) spectrum of xylcarpin C (**4**) in methanol-d_4 14
Figure S22. ^{13}C NMR (150 MHz) spectra of xylcarpin C (**4**) in methanol-d_4 14
Figure S23. ^{1}H NMR (600 MHz) spectrum of (24*R*)-22,23-dihydroxy-ergosta-4,6,8(14)-trien-3-one 23-*β*-D-glucopyranoside (**5**) in methanol-d_4 15
Figure S24. ^{13}C NMR (150 MHz) spectra of (24*R*)-22,23-dihydroxy-ergosta-4,6,8(14)-trien-3-one 23-*β*-D-glucopyranoside (**5**) in methanol-d_4 15
Figure S25. ^{1}H NMR (600 MHz) spectrum of 5-carboxymellein (**6**) in DMSO-d_6 16
Figure S26. ^{13}C NMR (150 MHz) spectra of 5-carboxymellein (**6**) in DMSO-d_6 16
Computational data for Compounds 1 and 2 17
Figure S27. Structures of compound **1** used for NMR and ECD (*β*-D-mannopyranose) calculations 17
Figure S28. Optimized conformers of compound **1** (*β*-D-mannopyranose) 17
Figure S29. Optimized conformers of compound **1** (*α*-D-mannopyranose) 18
Figure S30. DP4+ analysis of the calculated ^{13}C NMR data of compound **1** (*β*-D-mannopyranose) 19
Table S1 The calculated ^{13}C NMR data of compound **1** (*β*-D-mannopyranose) 20
Table S2 The calculated ^{13}C NMR data of compound **1** (*α*-D-mannopyranose) 24
Figure S31. Structures of compound **2** used for NMR and ECD (*β*-D-mannopyranose) calculations 26
Figure S32. Optimized conformers of compound **2** (*β*-D-mannopyranose) 26
Figure S33. Optimized conformers of compound **2** (*α*-D-mannopyranose) 27

Figure S34. DP4+ analysis of the calculated ^{13}C NMR data of compound **2** (*β*-D-mannopyranose)..... 28
Table S3 The calculated ^{13}C NMR data of compound **2** (*β*-D-mannopyranose)……….……………29
Table S4 The calculated ^{13}C NMR data of compound **2** (*α*-D-mannopyranose)……….……………31
Figure S35. Preparative HPLC chromatogram of mannosylxylarinol A (**1**)... 35
Figure S36. Preparative HPLC chromatogram of mannosylxylarinol B (**2**)... 35
Figure S37. Preparative HPLC chromatogram of 2-hexylidene-3-methylsuccinic acid (**3**)................... 35
Figure S38. Preparative HPLC chromatogram of xylcarpin C (**4**) 35
Figure S39. Preparative HPLC chromatogram of (24*R*)-22,23-dihydroxy-ergosta-4,6,8(14)-trien-3-one 23-*β*-D-glucopyranoside (**5**) 36
Figure S40 HPLC chromatogram for sugar derivatization of compound **2** and derivatized D-mannose and L-mannose standards 36

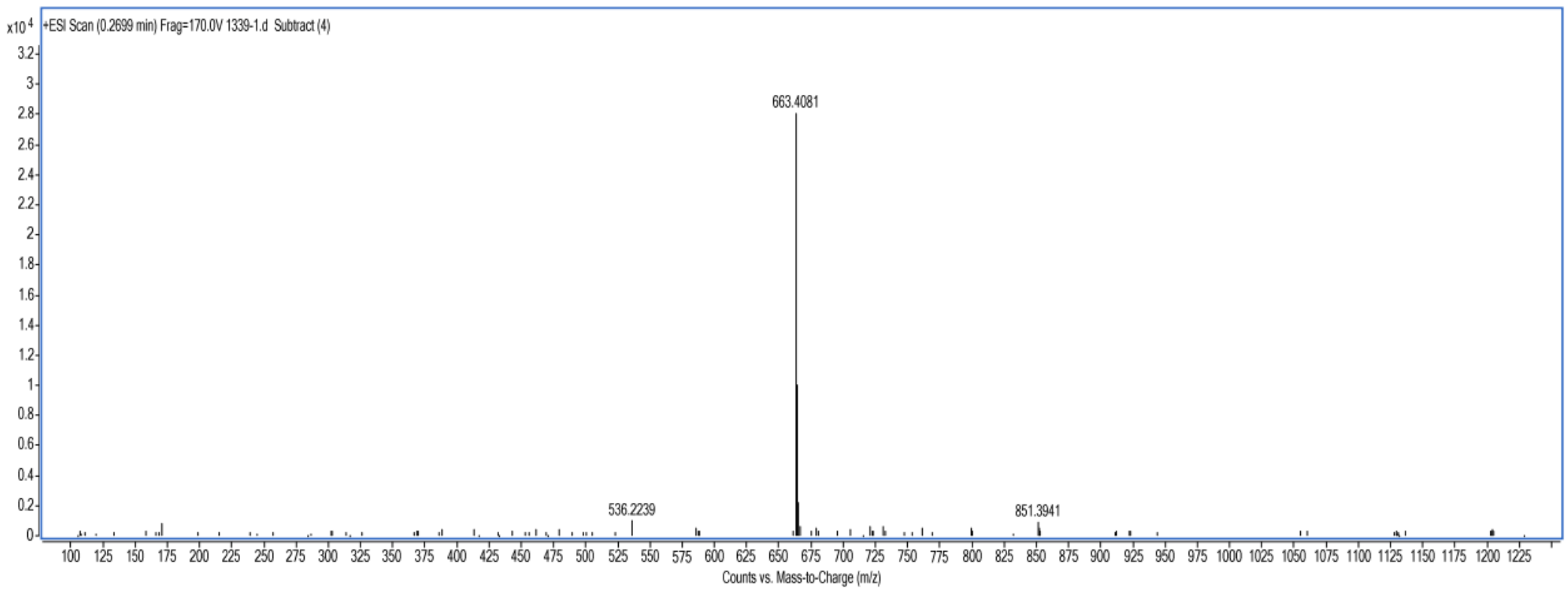

Figure S1. HRESIMS spectrum of mannosylxylarinol A (**1**)

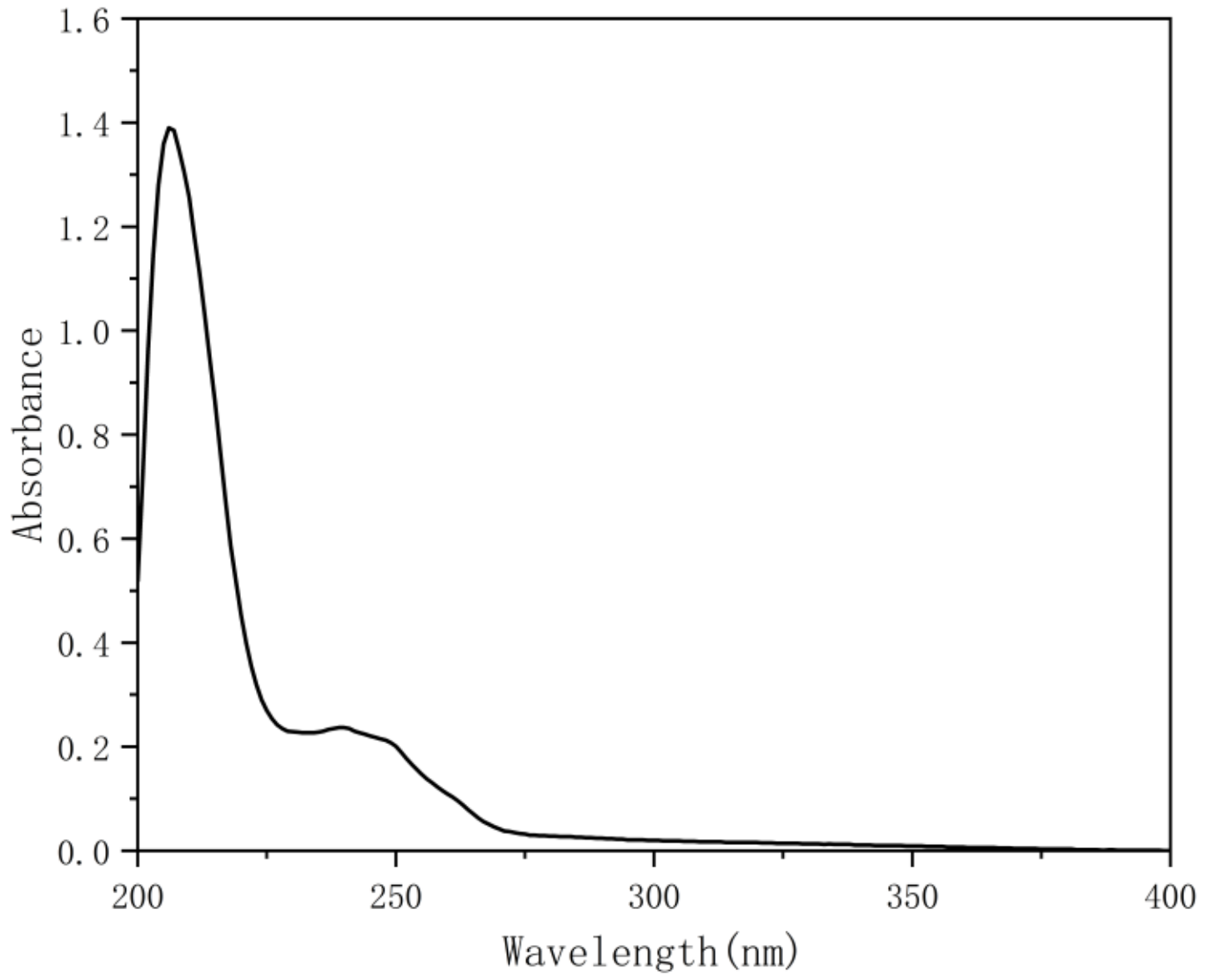

Figure S2. UV spectrum of mannosylxylarinol A (**1**)

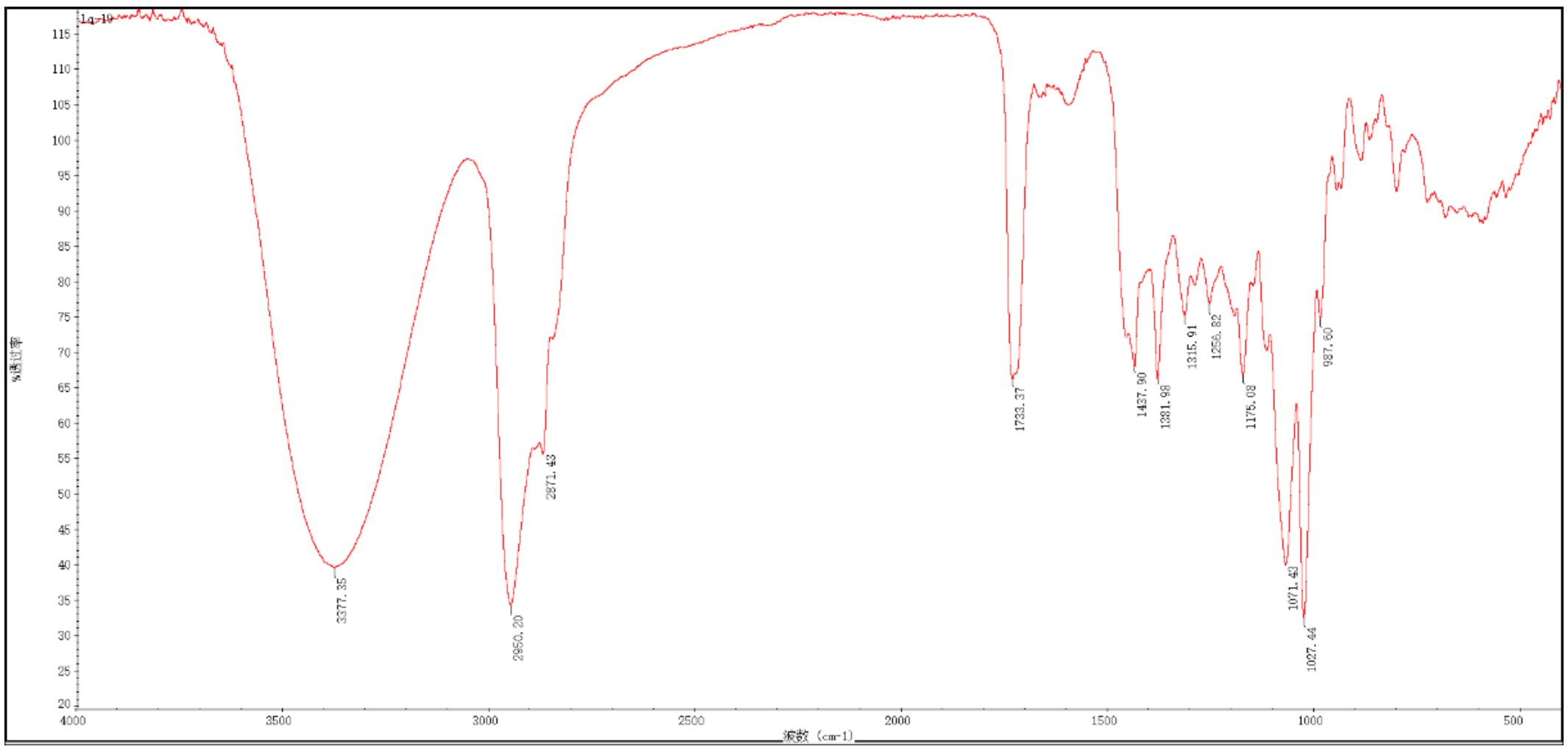

Figure S3. IR spectrum of mannosylxylarinol A (**1**)

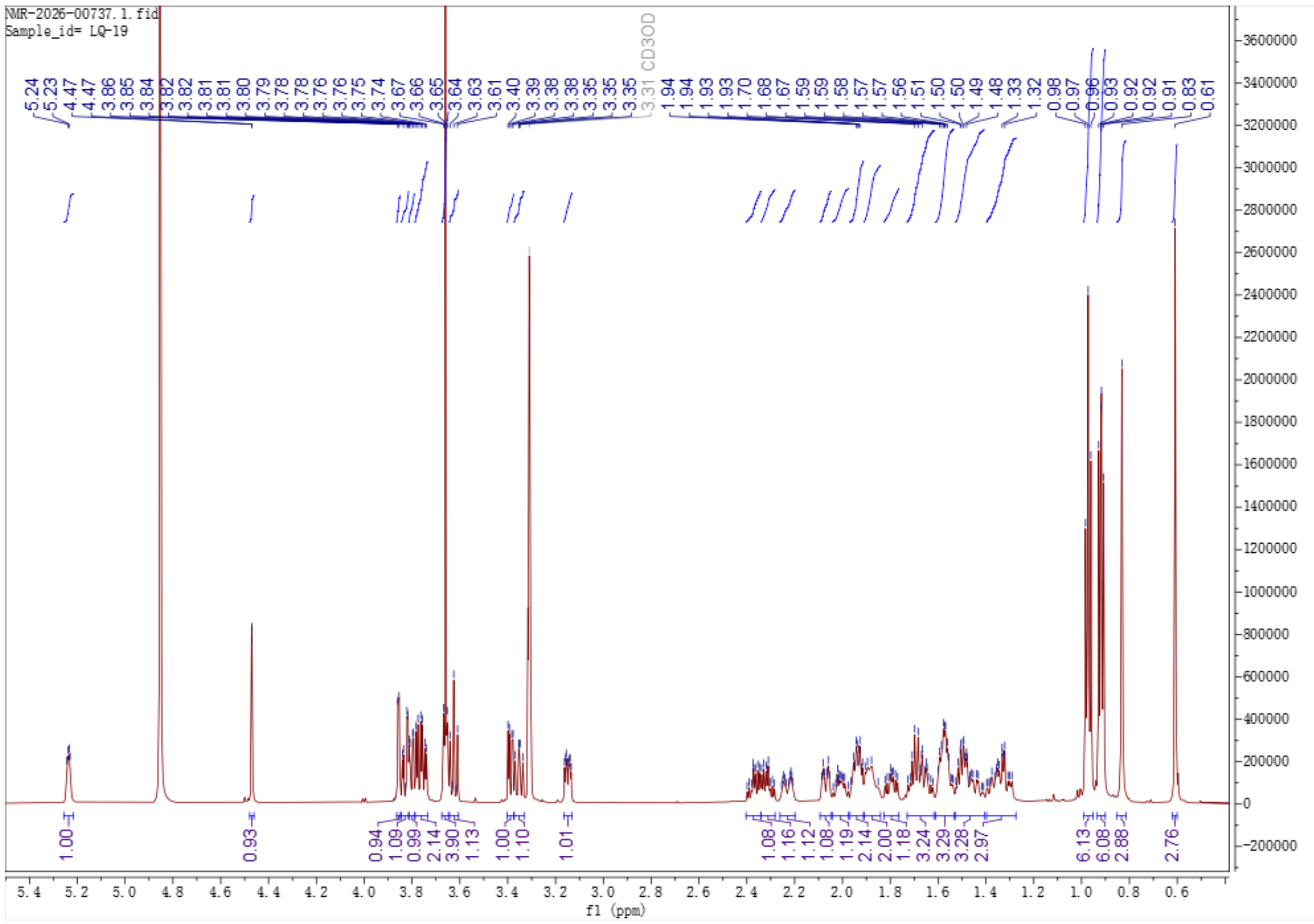

Figure S4. ^{1}H NMR (600 MHz) spectrum of mannosylxylarinol A (**1**) in methanol-d_4

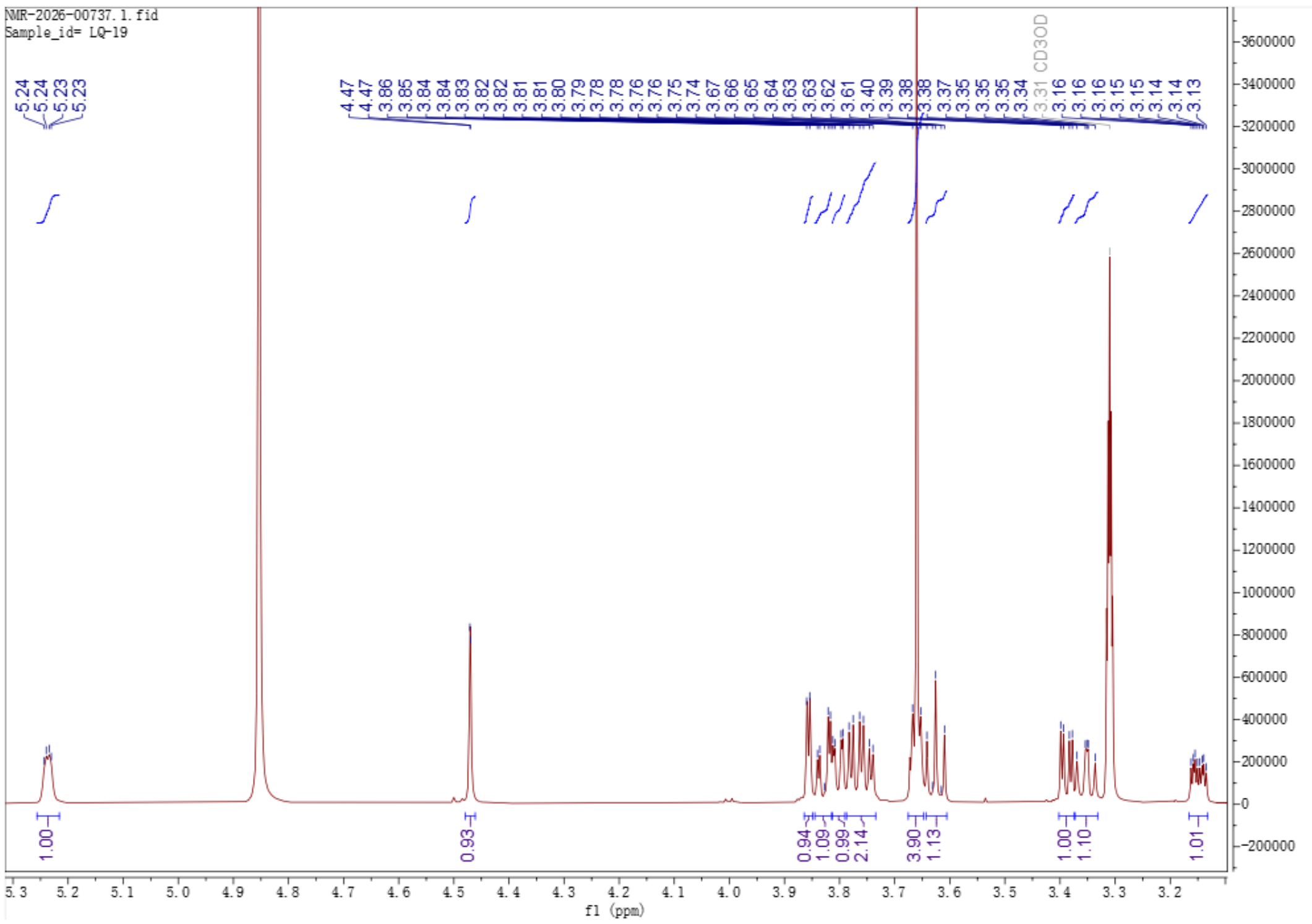

Figure S4-1. Enlarged ^{1}H NMR (600 MHz) spectrum of mannosylxylarinol A (**1**) in methanol-d_4

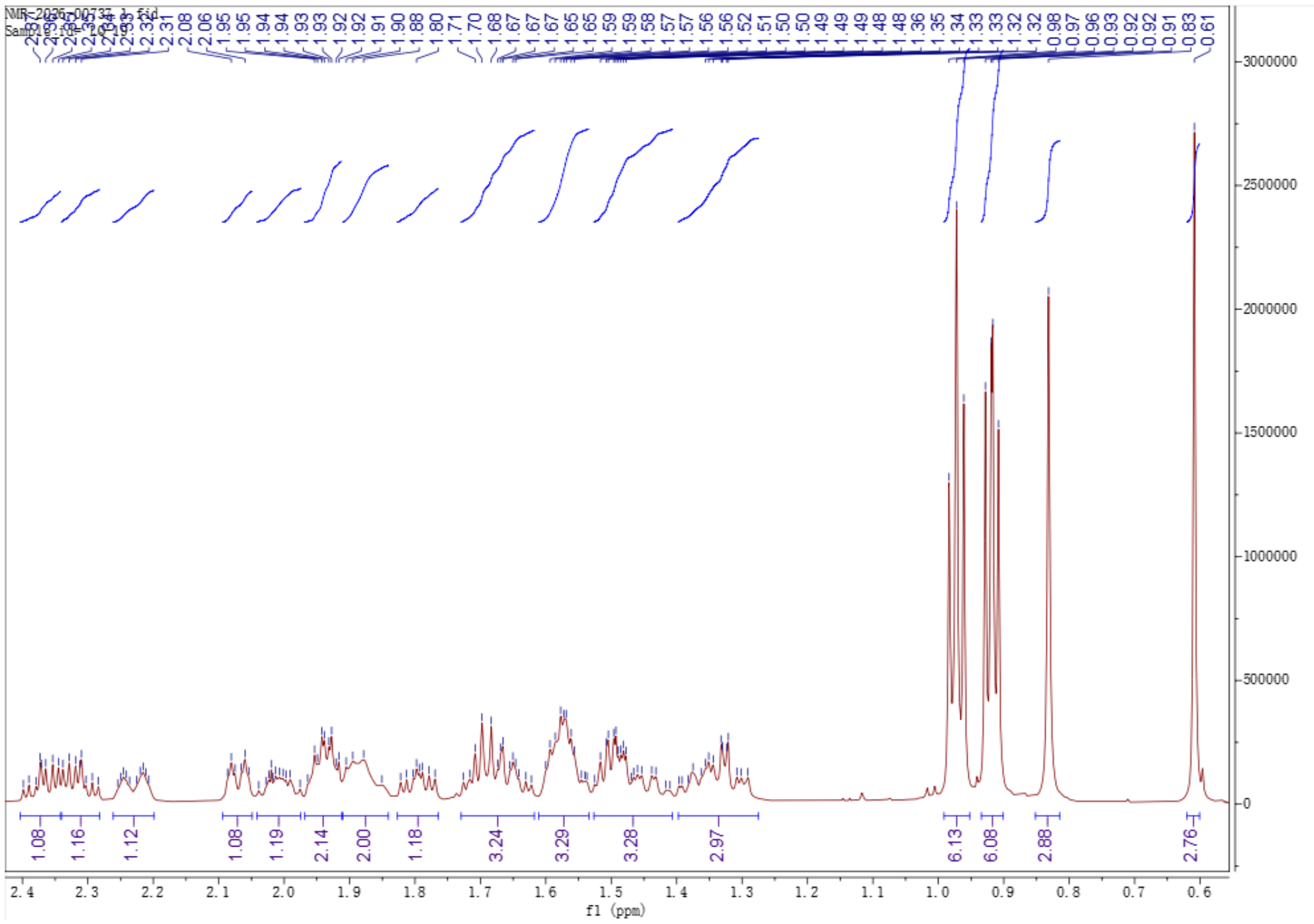

Figure S4-2. Enlarged ^{1}H NMR (600 MHz) spectrum of mannosylxylarinol A (**1**) in methanol-d_4

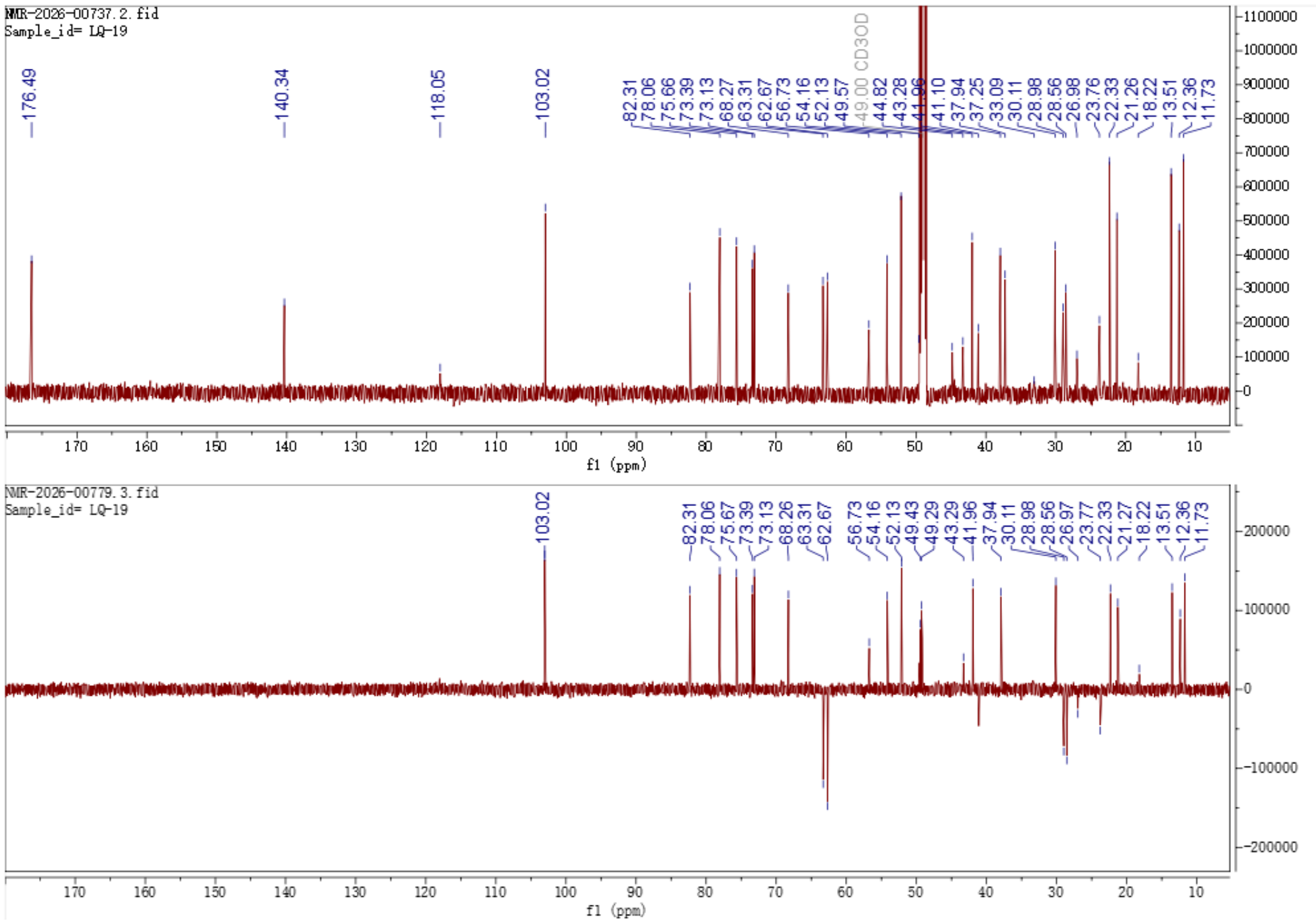

Figure S5. ^{13}C NMR and DEPT 135 (150 MHz) spectra of mannosylxylarinol A (**1**) in methanol-d_4

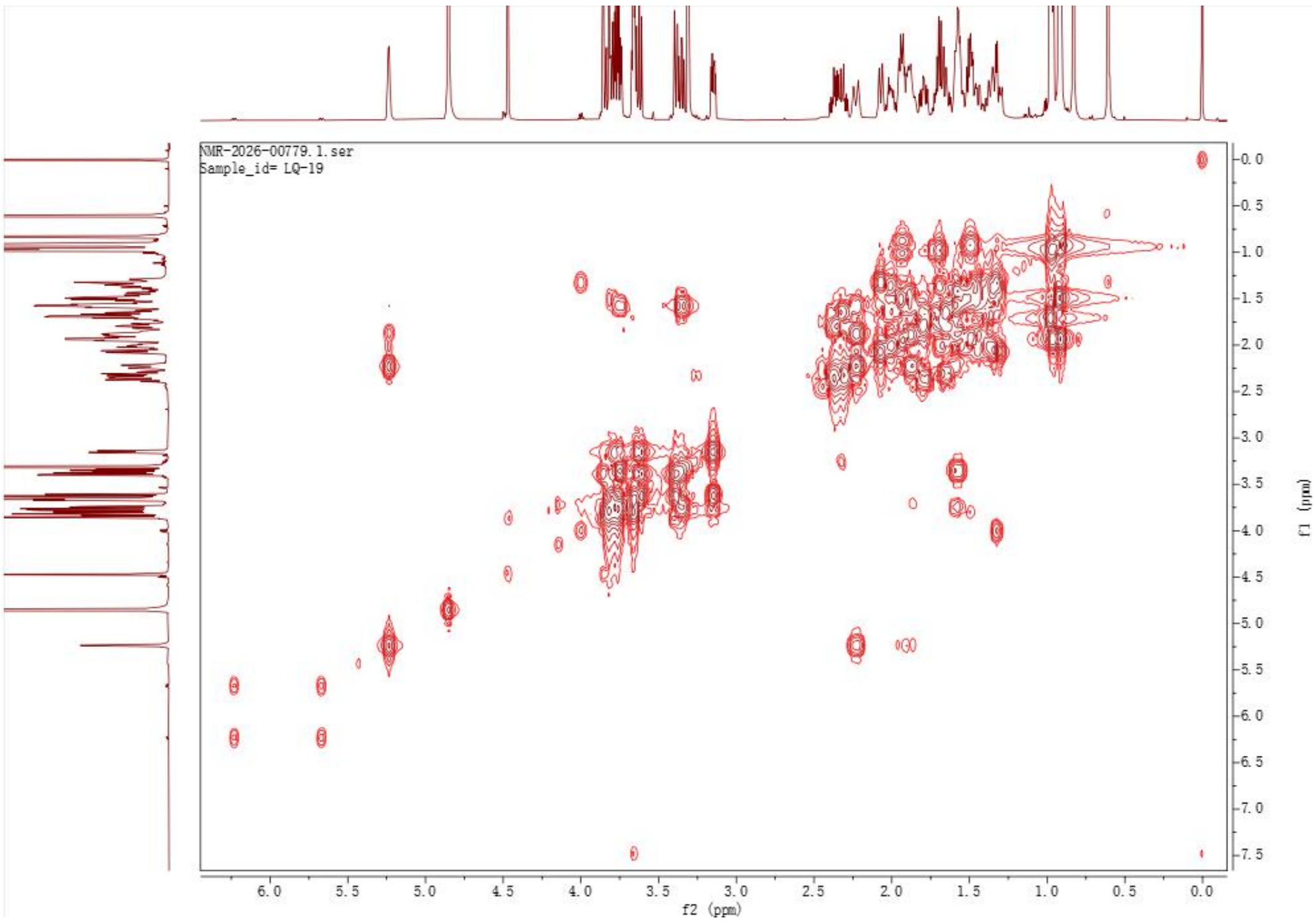

Figure S6. ¹H–¹H COSY spectrum of mannosylxylarinol A (**1**) in methanol-d_4

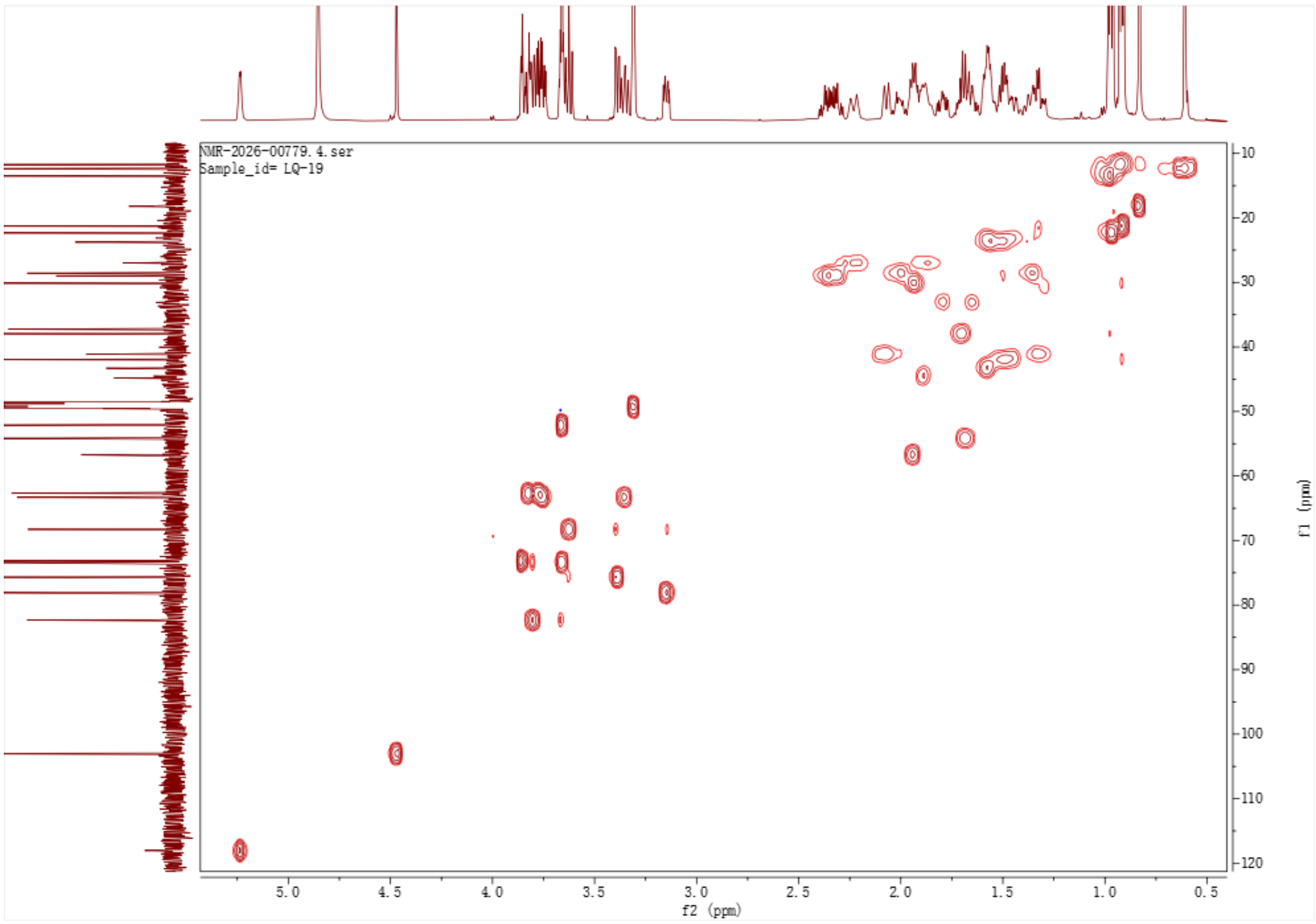

Figure S7. HSQC spectrum of mannosylxylarinol A (**1**) in methanol-d_4

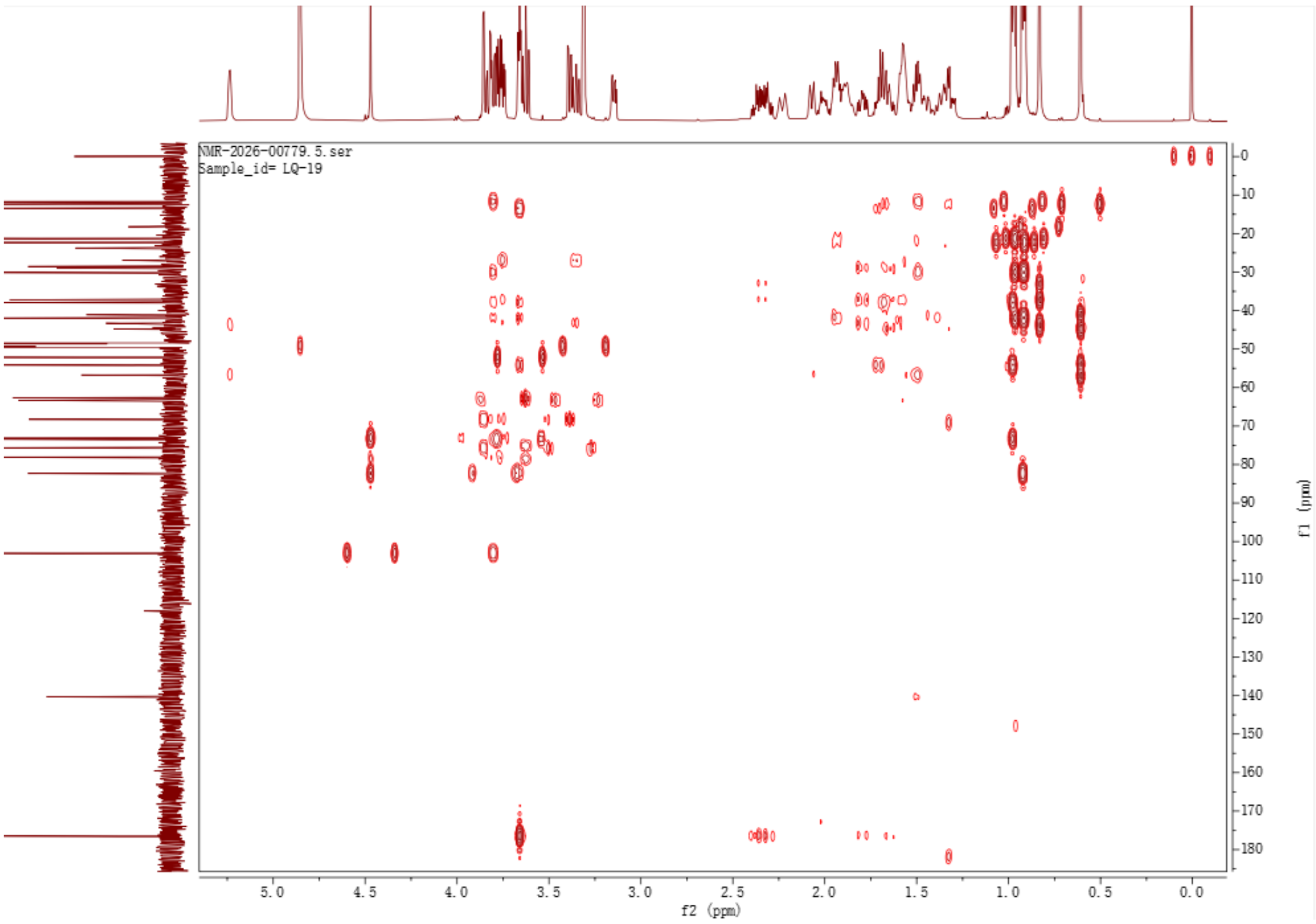

Figure S8. HMBC spectrum of mannosylxylarinol A (**1**) in methanol-d_4

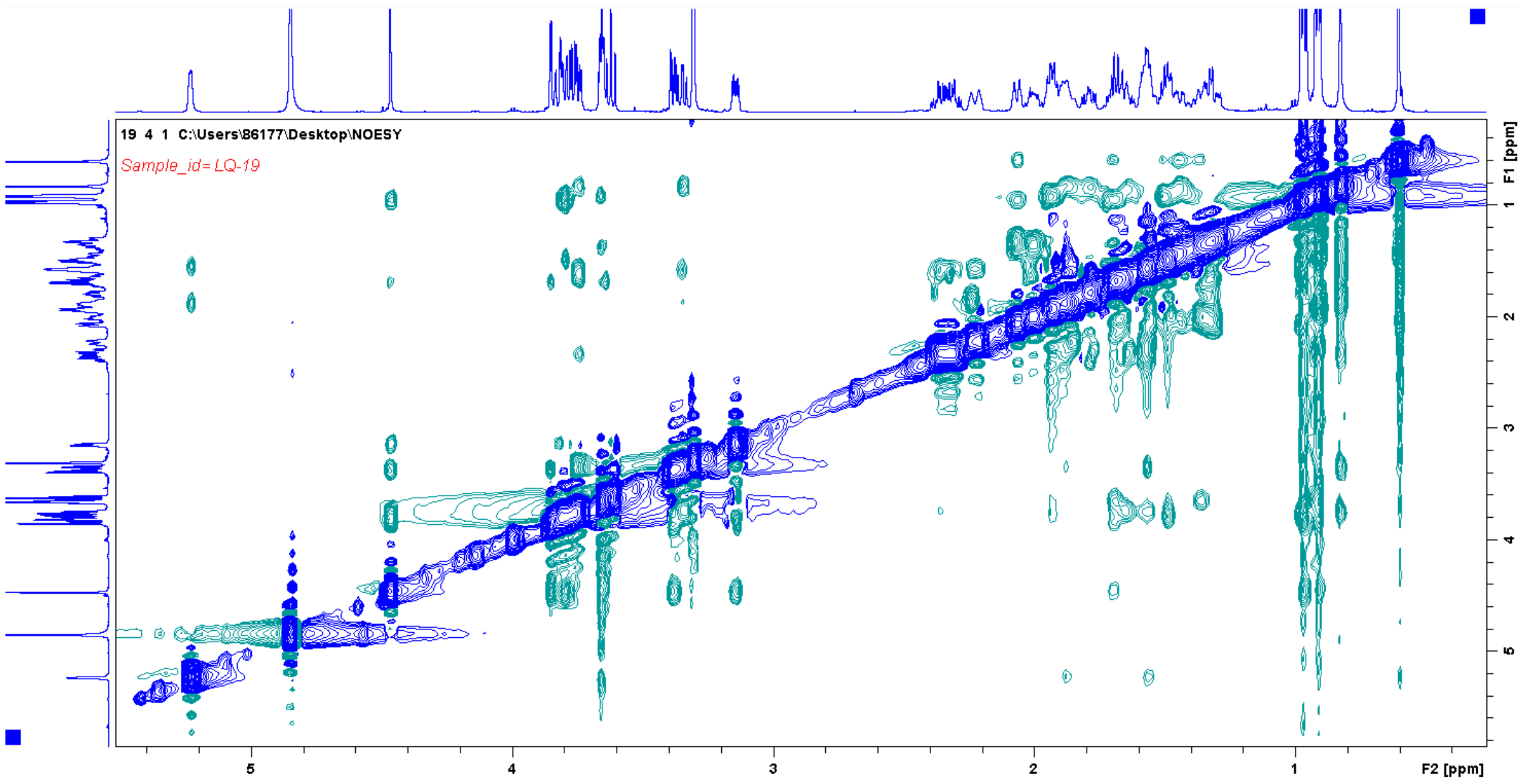

Figure S9. NOESY spectrum of mannosylxylarinol A (**1**) in methanol-d_4

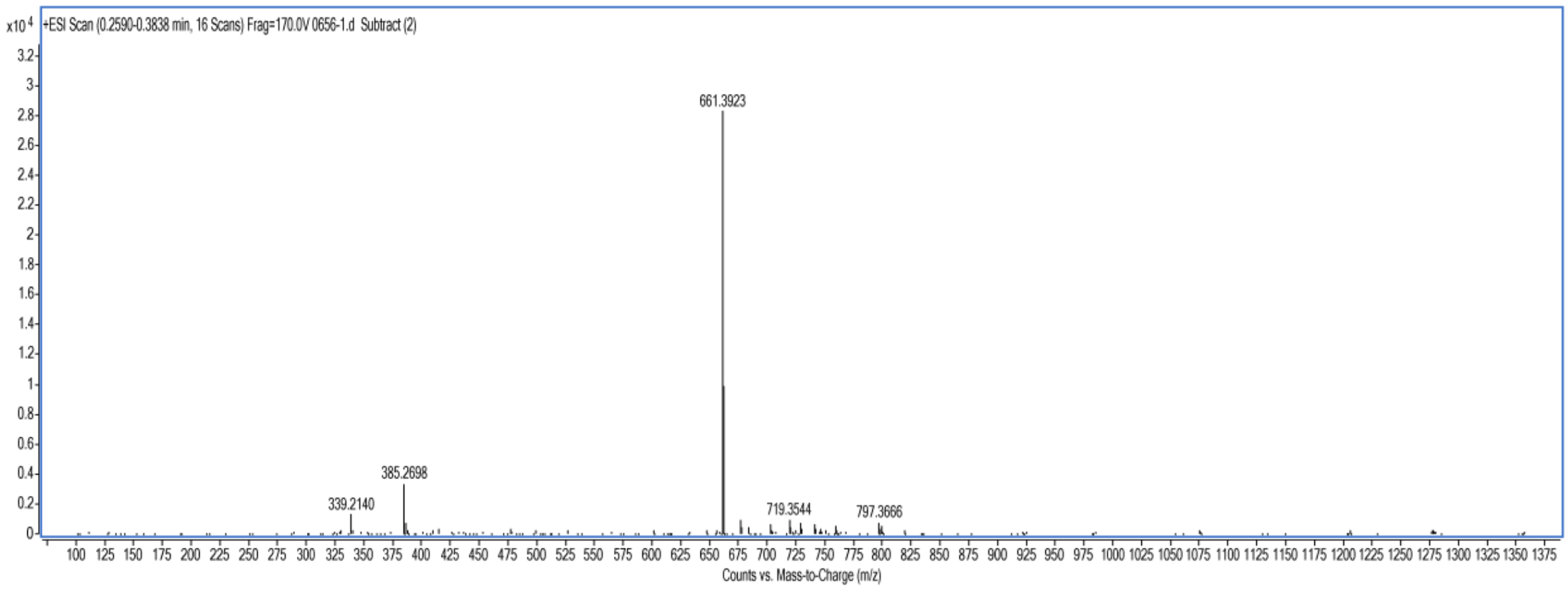

Figure S10. HRESIMS spectrum of mannosylxylarinol B (**2**)

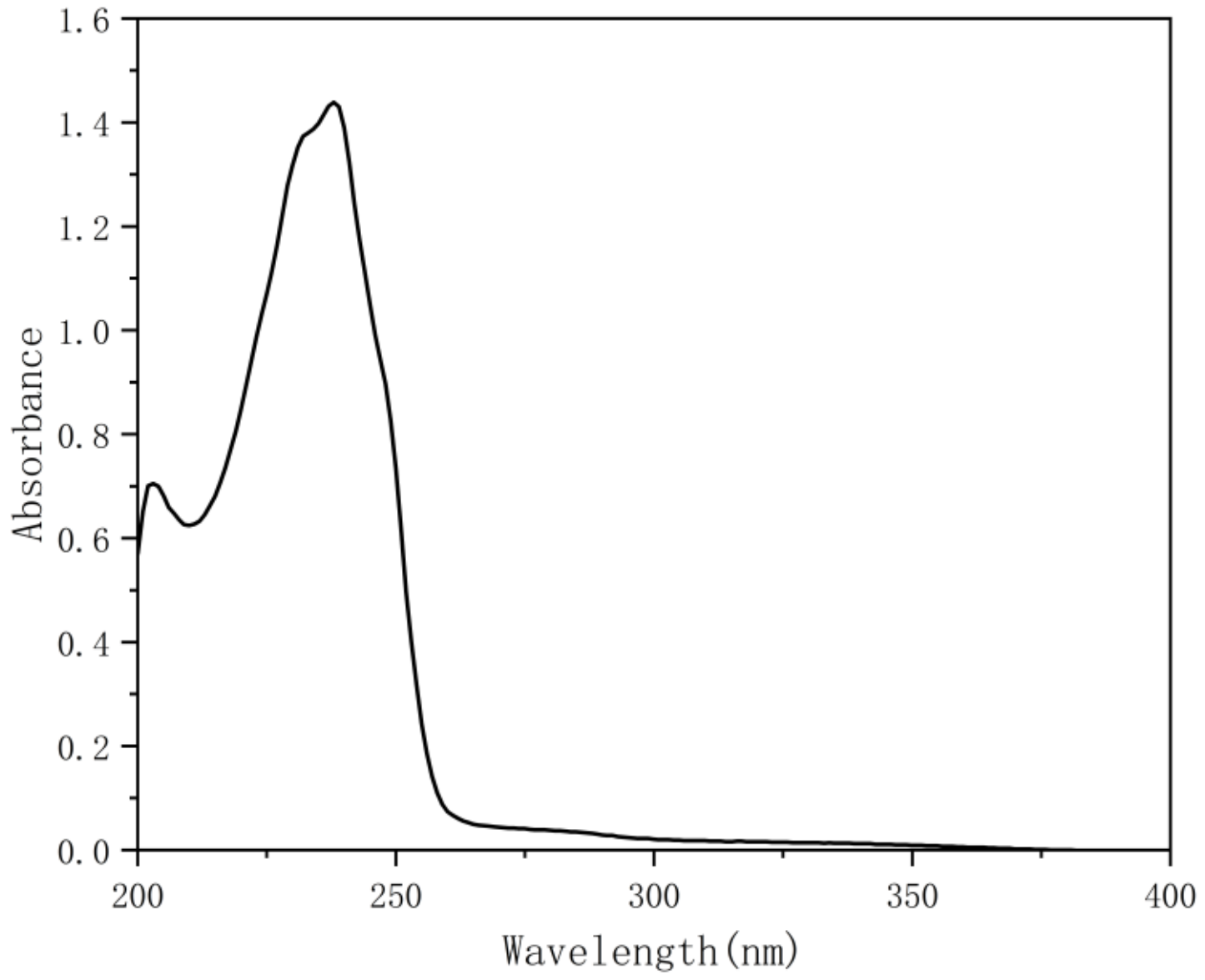

Figure S11. UV spectrum of mannosylxylarinol B (**2**)

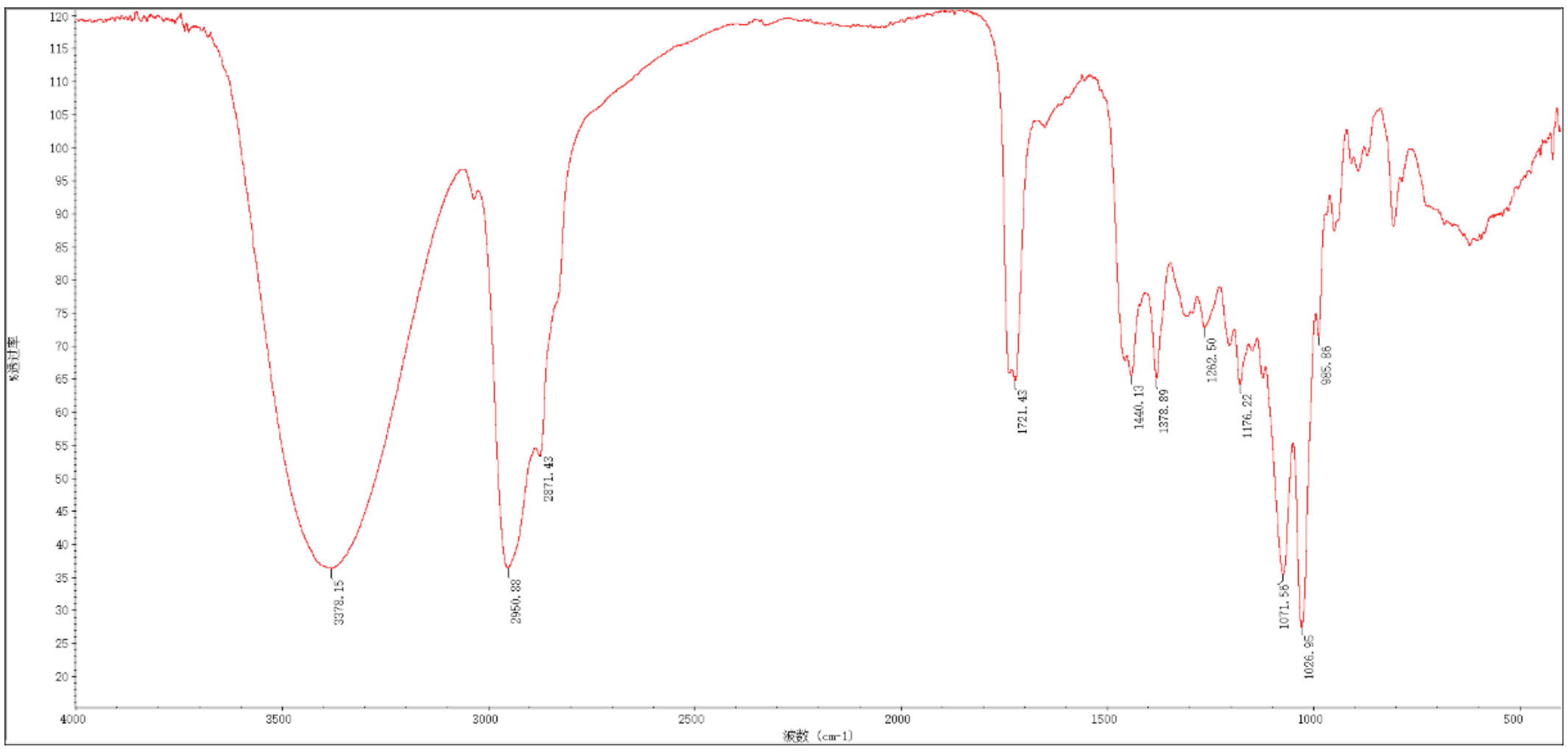

Figure S12. IR spectrum of mannosylxylarinol B (**2**)

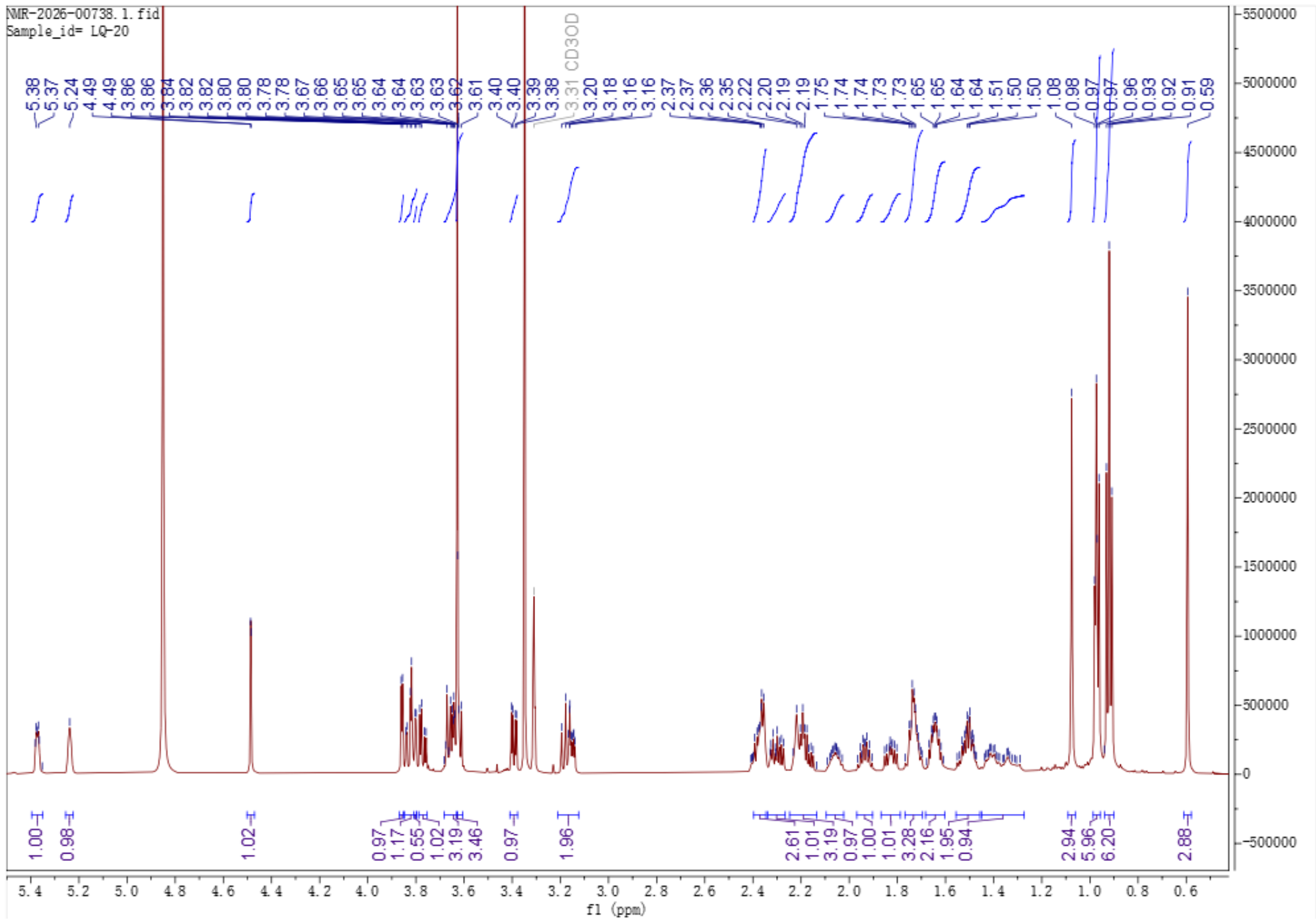

Figure S13. ^{1}H NMR (600 MHz) spectrum of mannosylxylarinol B (**2**) in methanol-d_4

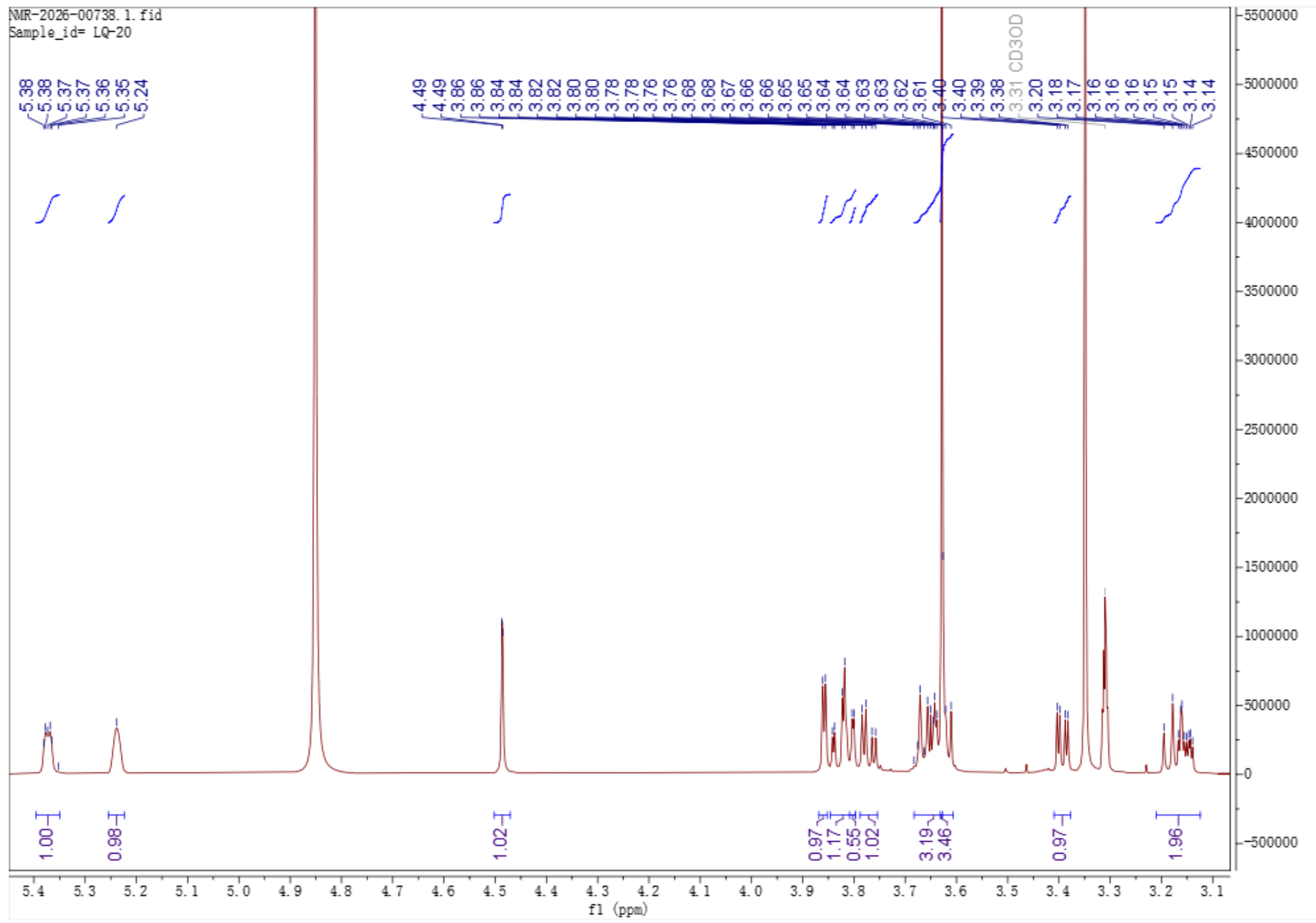

Figure S13-1. Enlarged ^{1}H NMR (600 MHz) spectrum of mannosylxylarinol B (**2**) in methanol-d_4

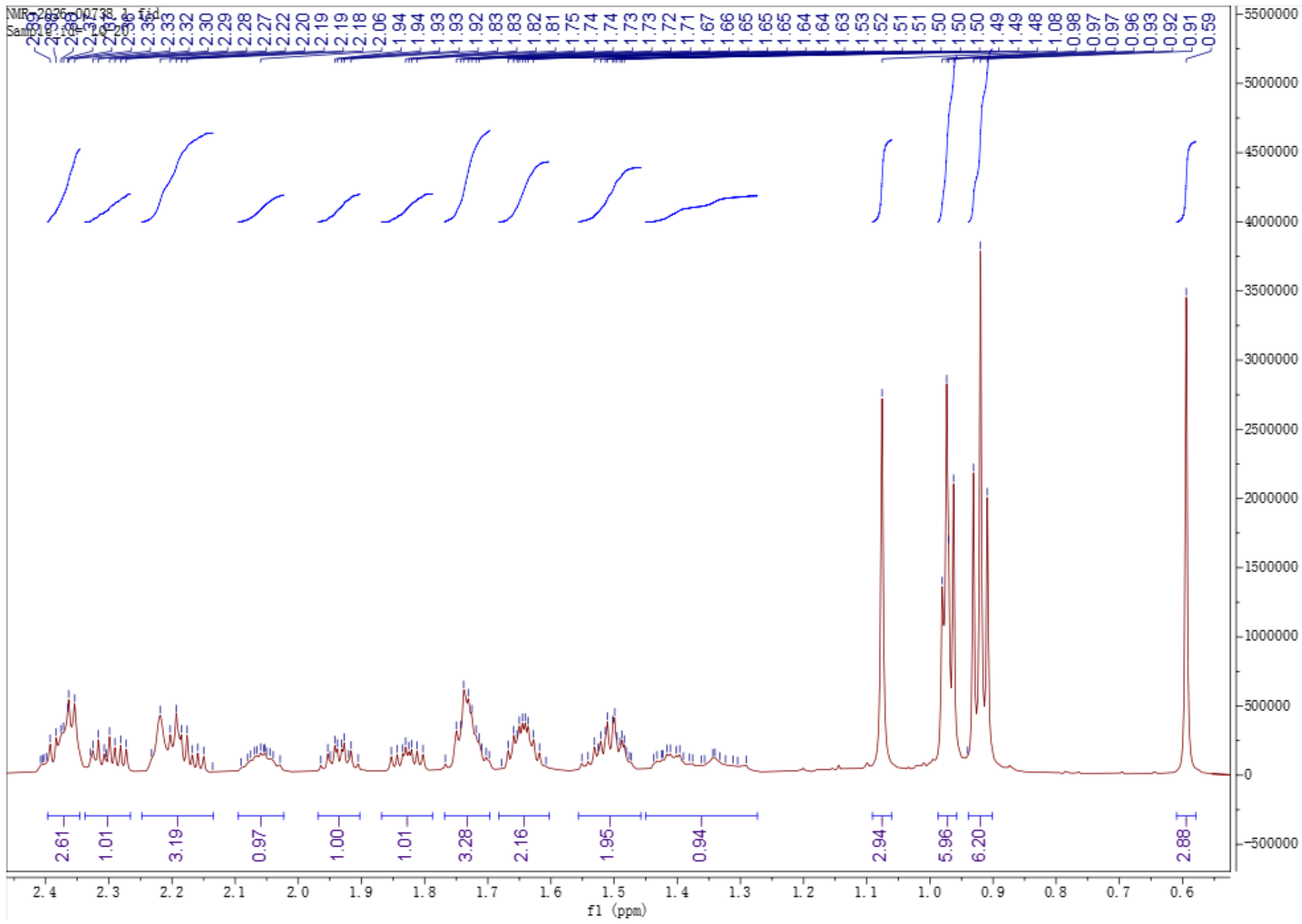

Figure S13-2. Enlarged ^{1}H NMR (600 MHz) spectrum of mannosylxylarinol B (**2**) in methanol-d_4

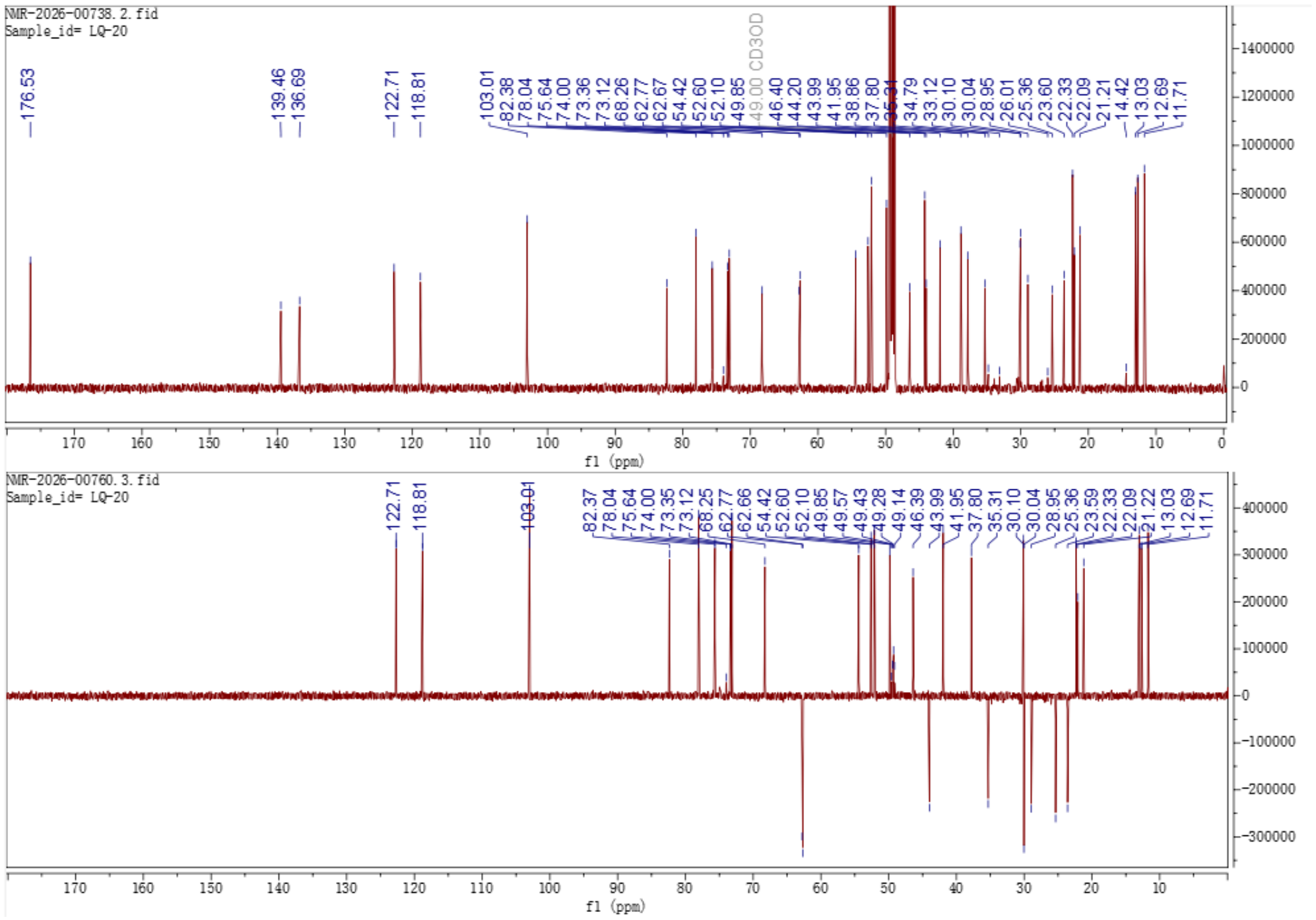

Figure S14. ^{13}C NMR and DEPT 135 (150 MHz) spectra of mannosylxylarinol B (**2**) in methanol-d_4

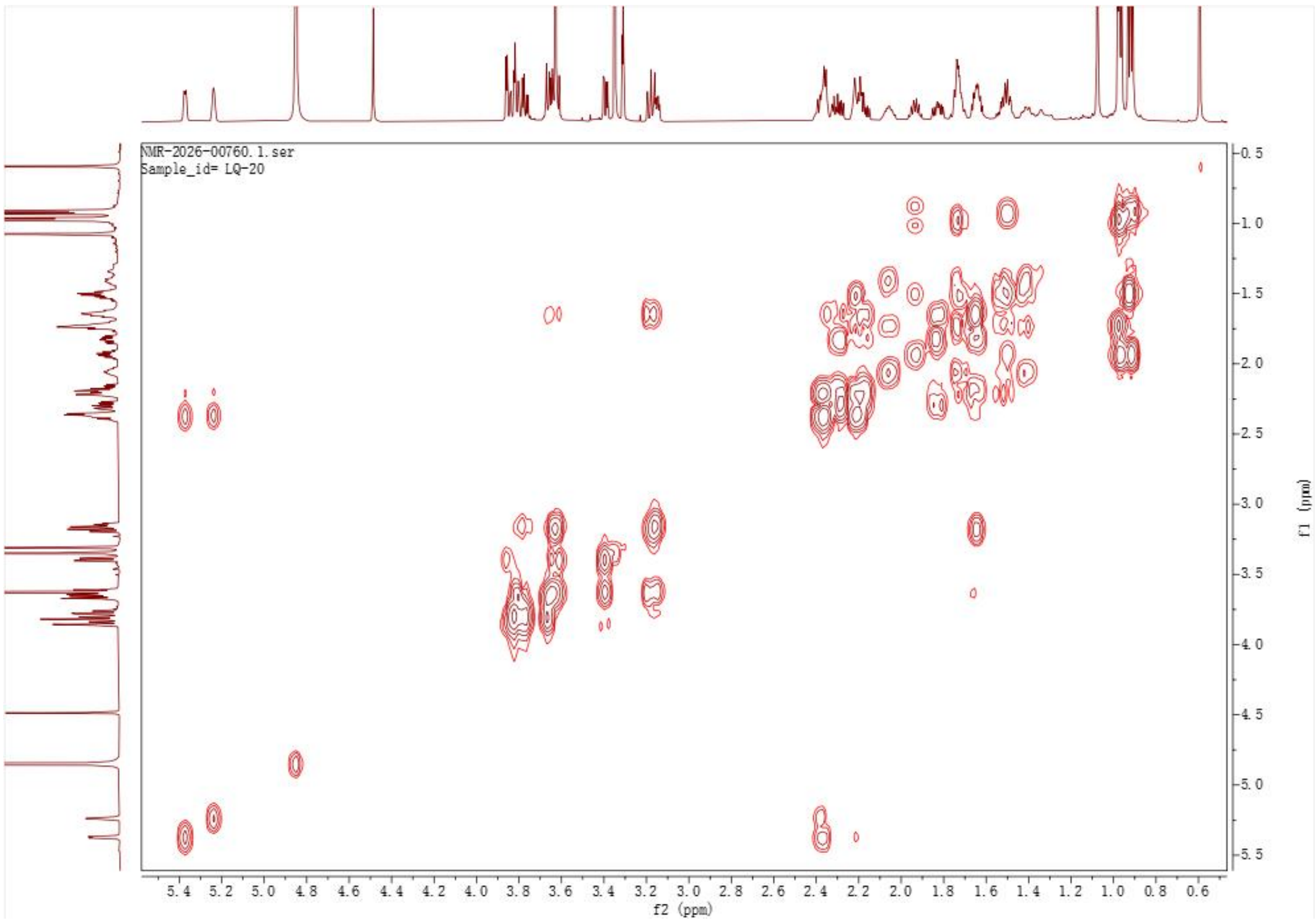

Figure S15. ^{1}H–^{1}H COSY spectrum of mannosylxylarinol B (**2**) in methanol-d_4

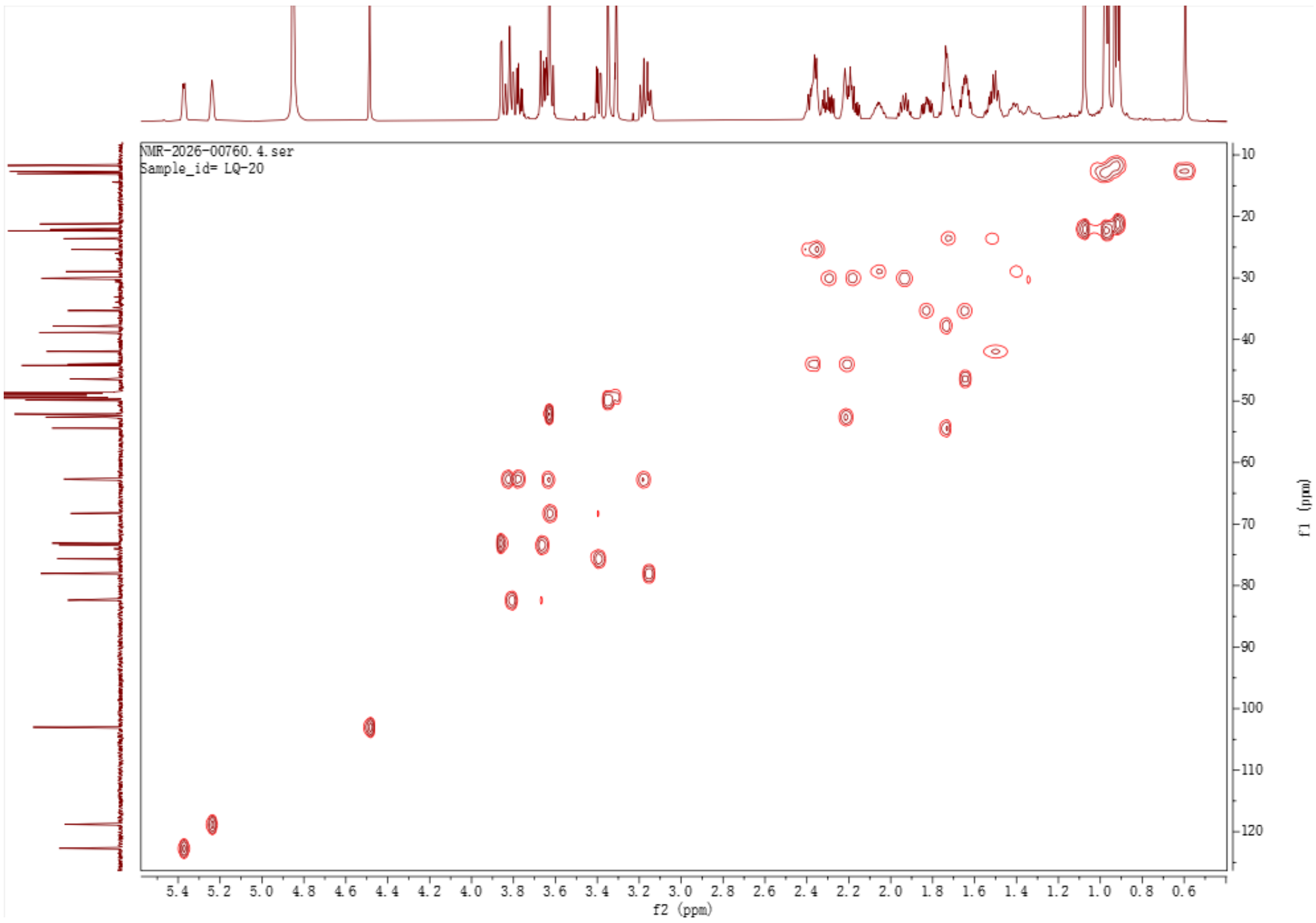

Figure S16. HSQC spectrum of mannosylxylarinol B (**2**) in methanol-d_4

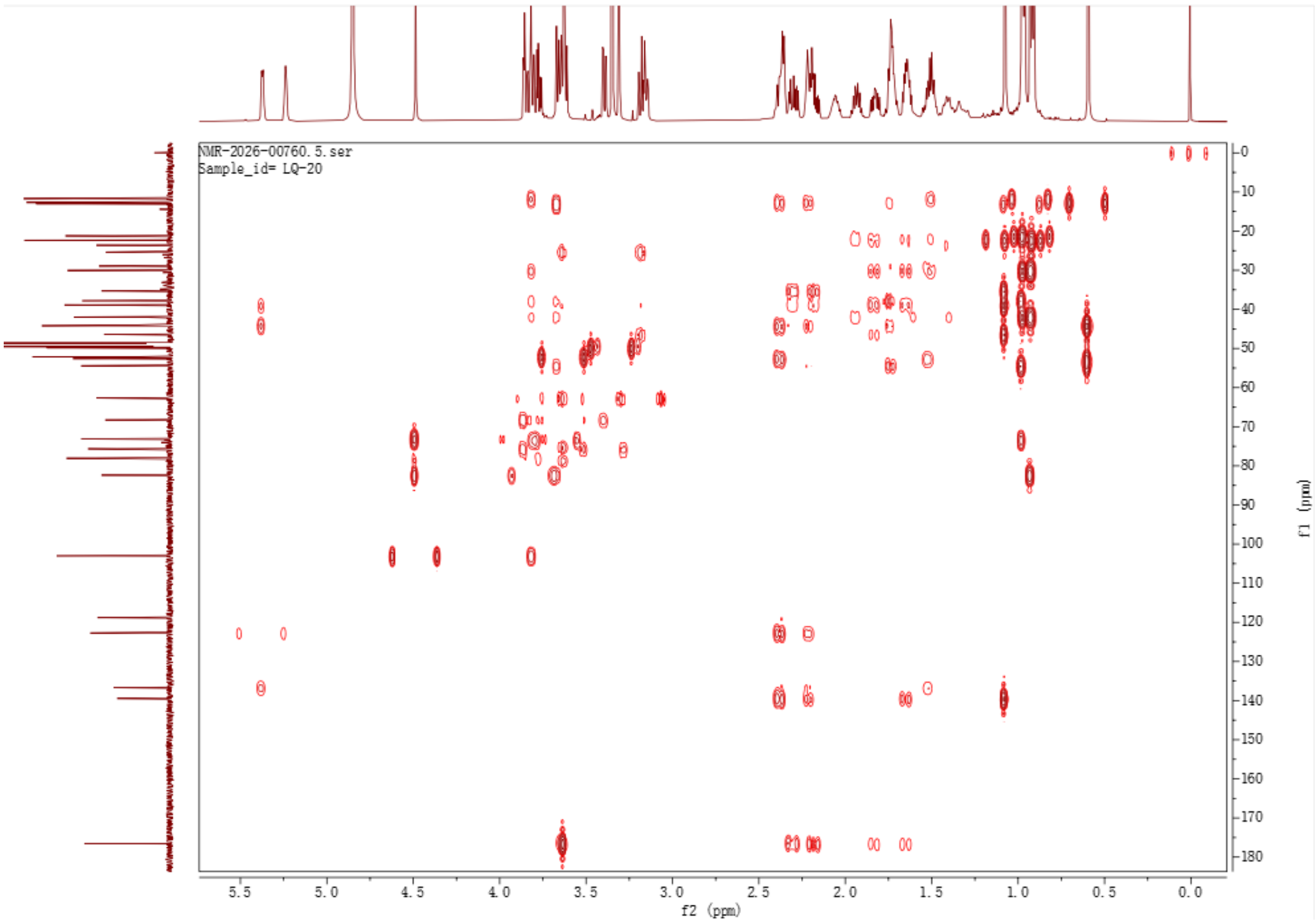

Figure S17. HMBC spectrum of mannosylxylarinol B (**2**) in methanol-d_4

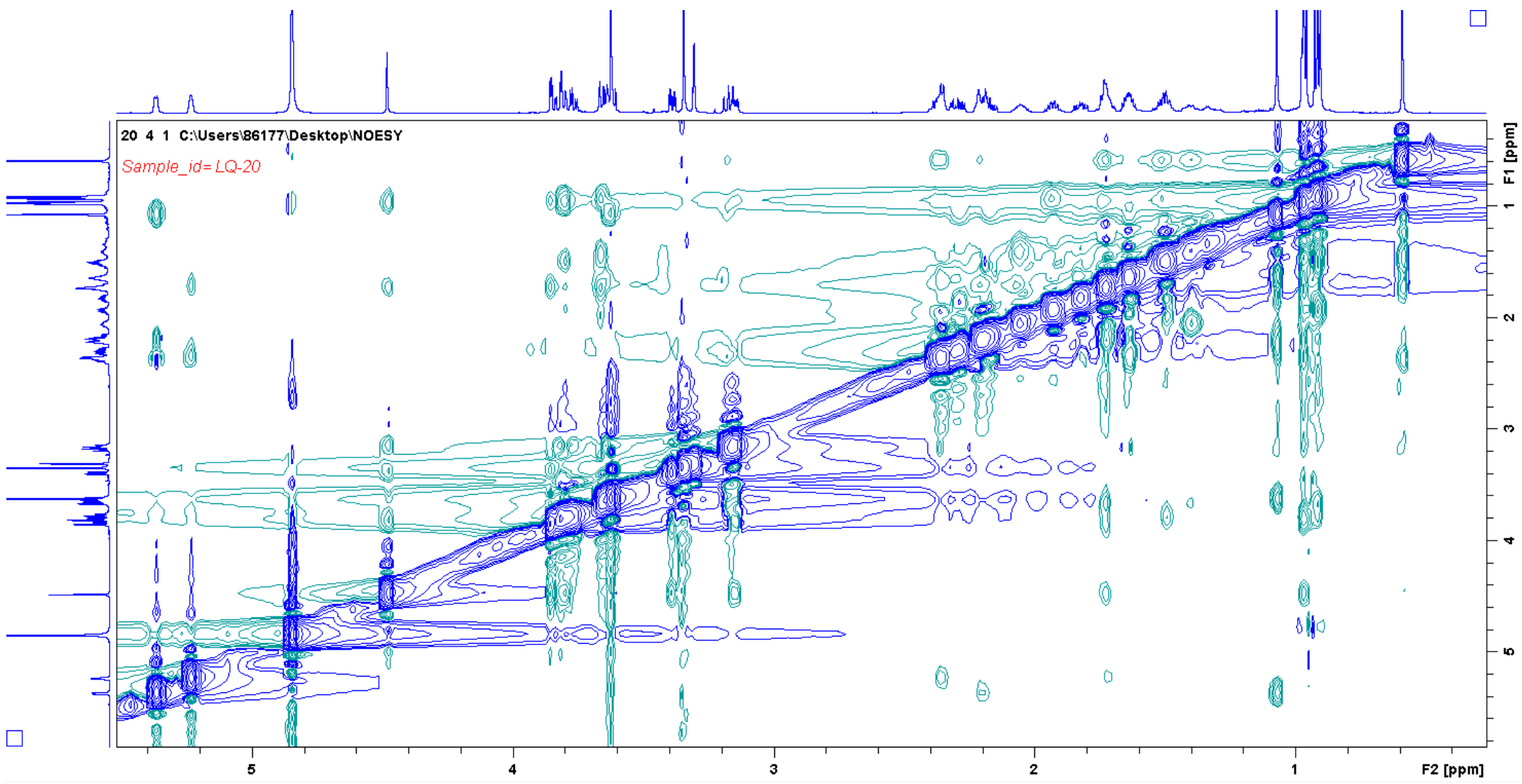

Figure S18. NOESY spectrum of mannosylxylarinol B (**2**) in methanol-d_4

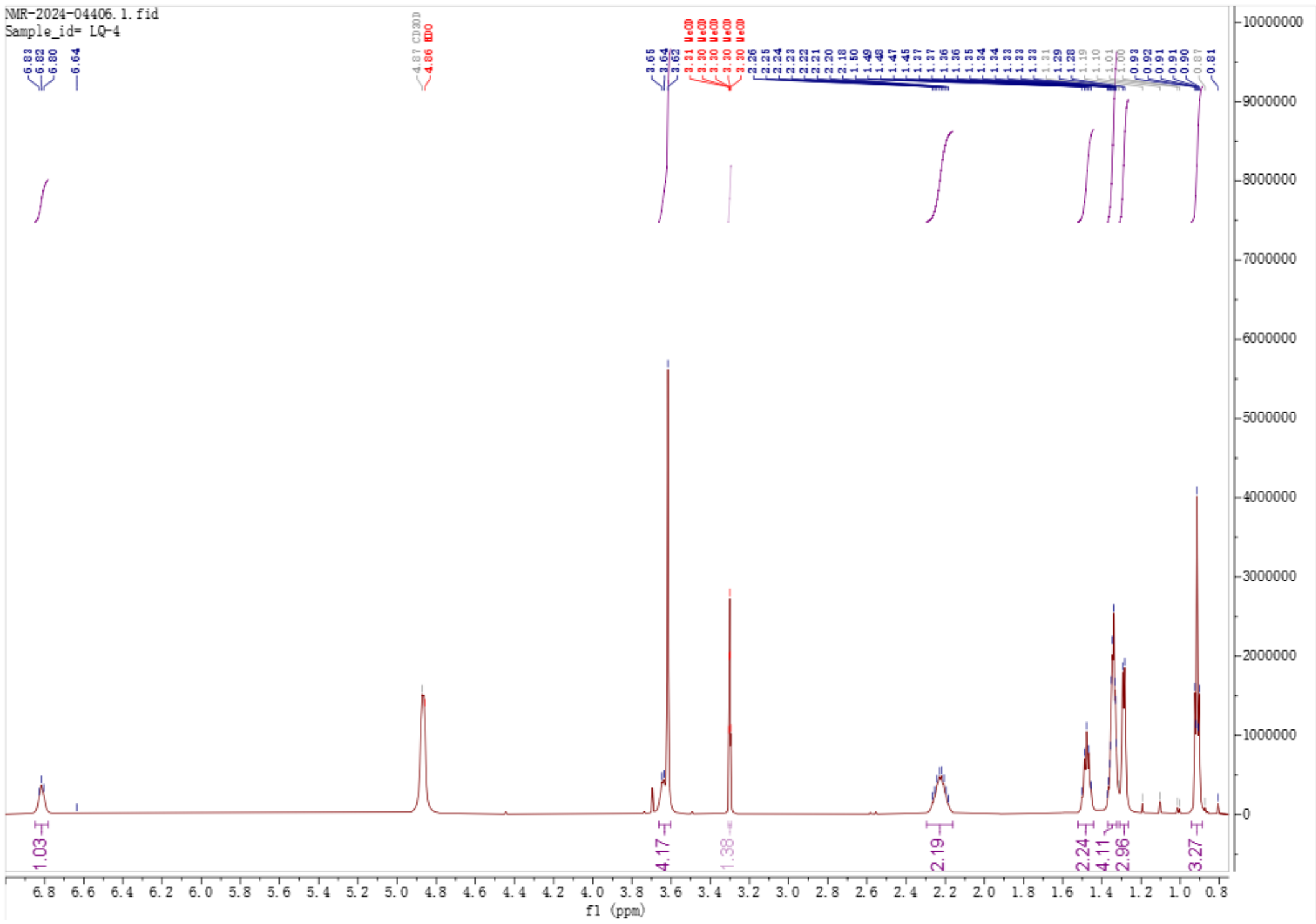

Figure S19. ^{1}H NMR (600 MHz) spectrum of 2-hexylidene-3-methylsuccinic acid (**3**) in methanol-d_4

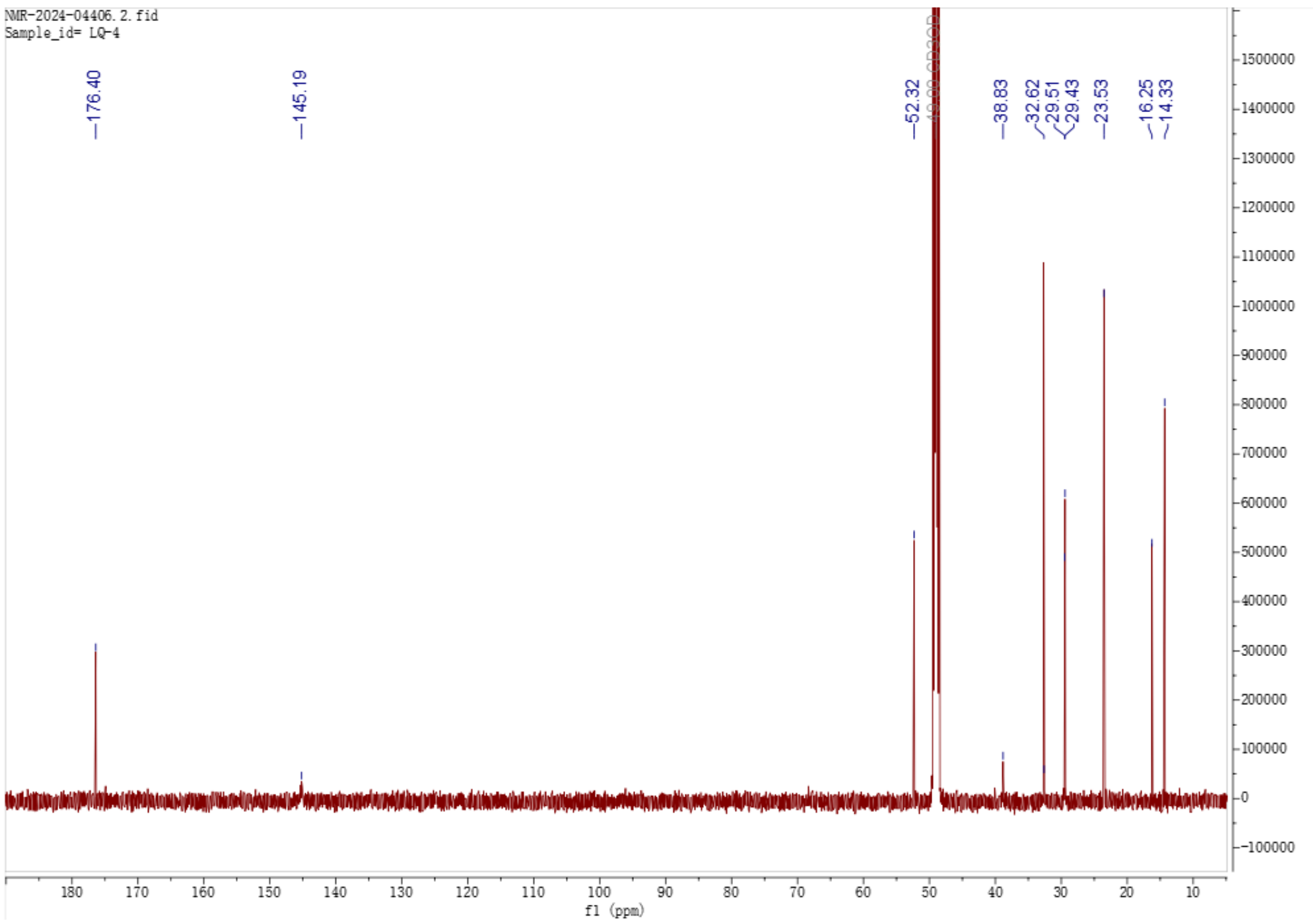

Figure S20. ^{13}C NMR spectra of 2-hexylidene-3-methylsuccinic acid (**3**) in methanol-d_4

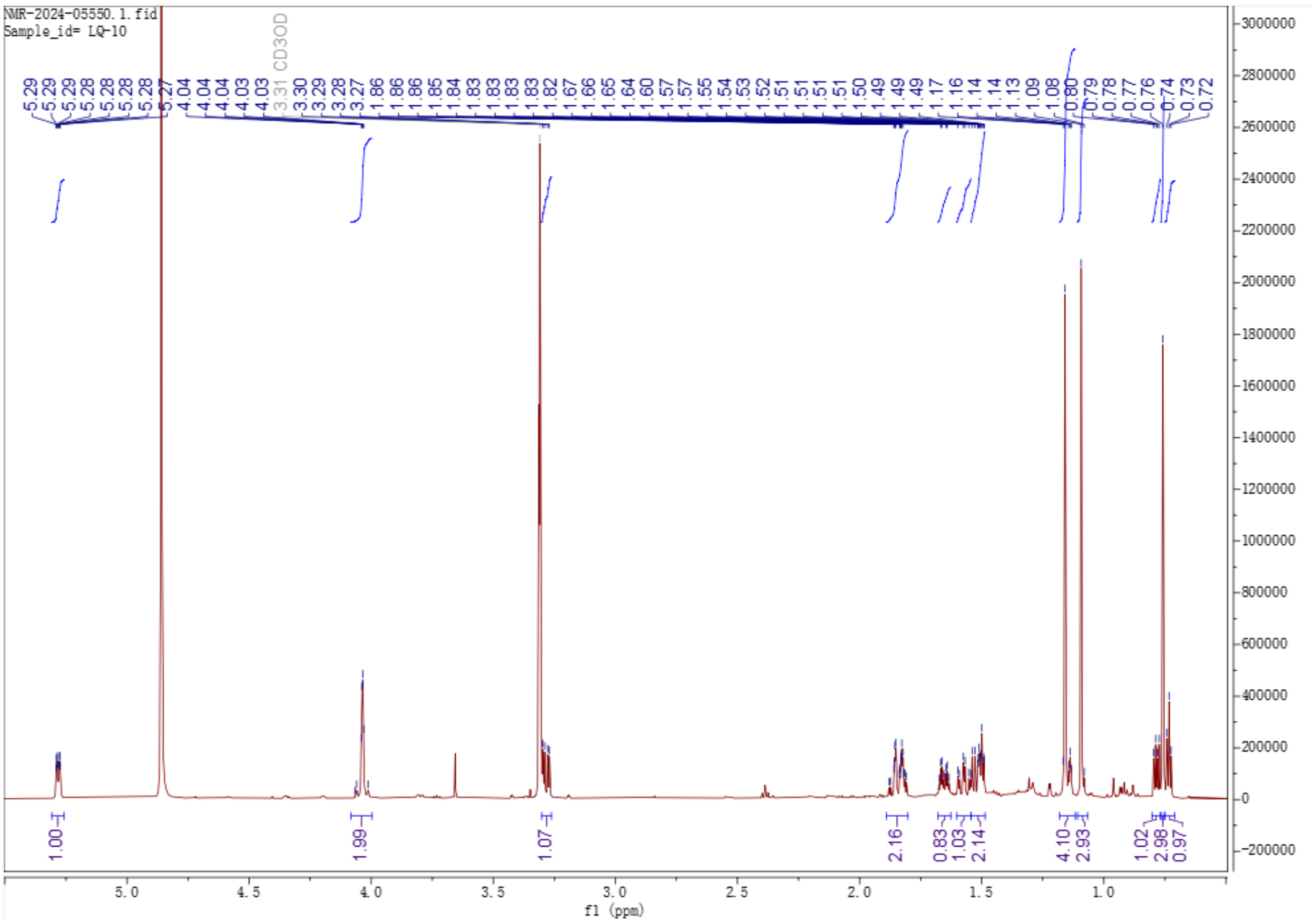

Figure S21. ^{1}H NMR (600 MHz) spectrum of xylcarpin C (**4**) in methanol-d_4

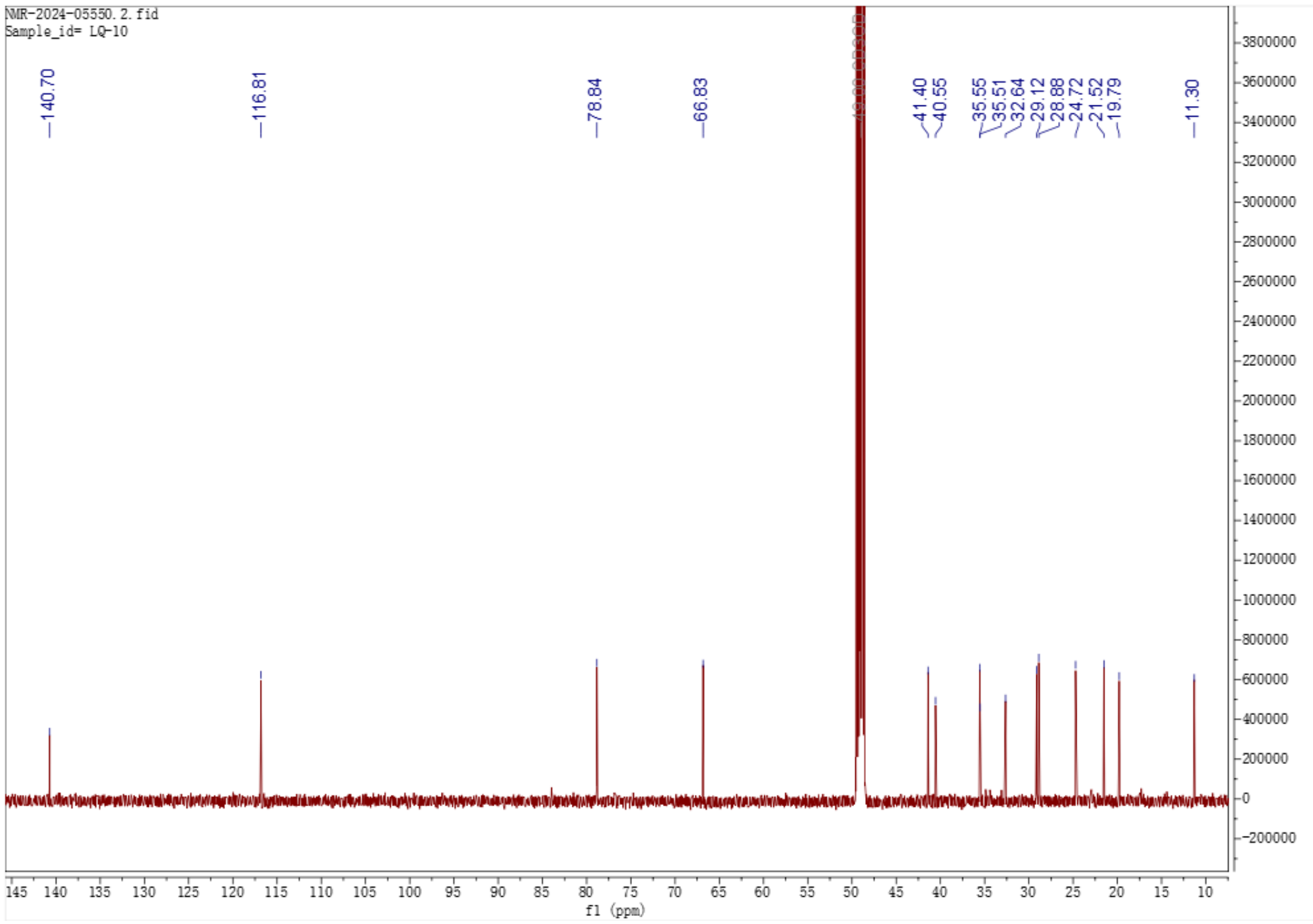

Figure S22. ^{13}C NMR spectra of xylcarpin C (**4**) in methanol-d_4

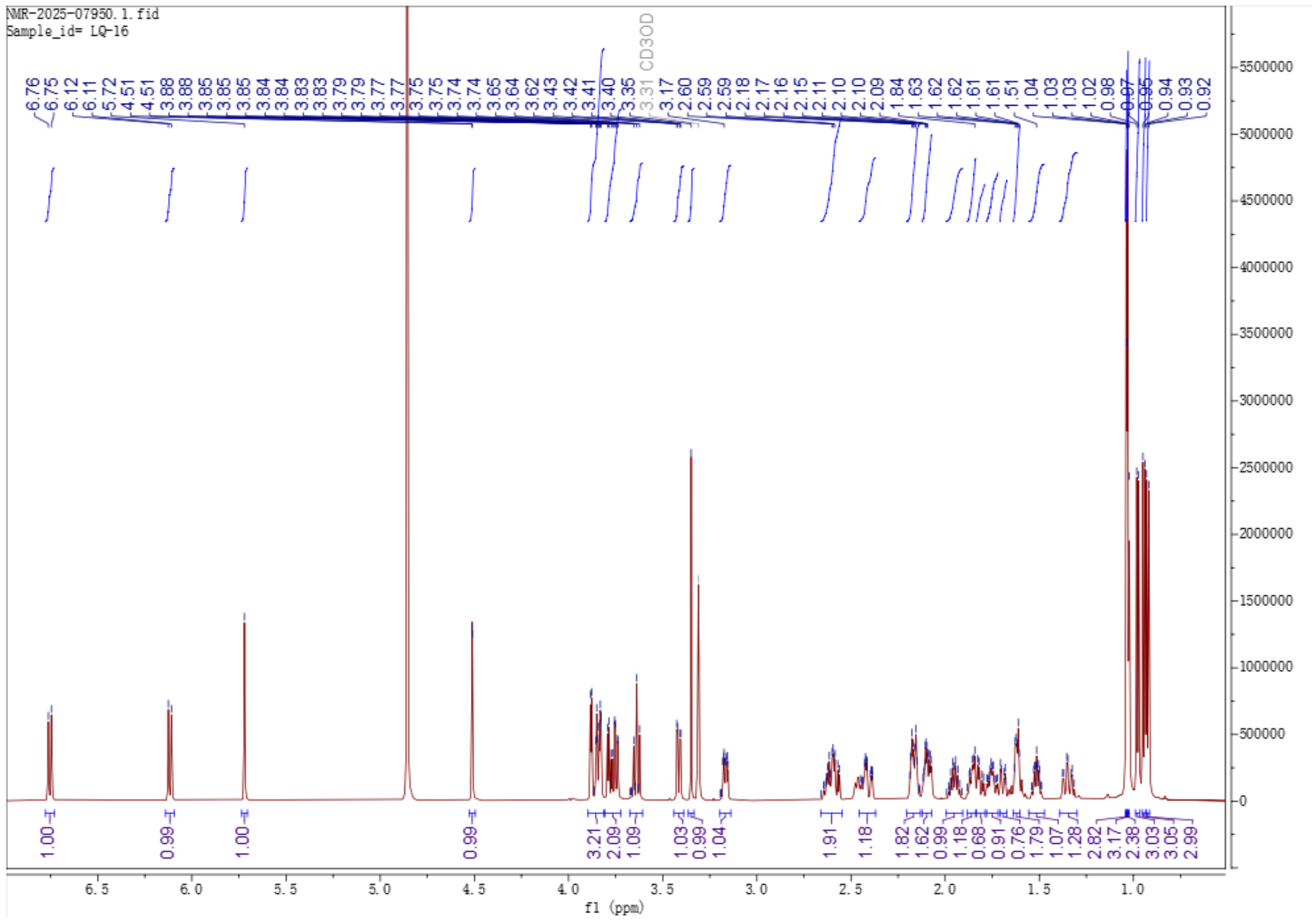

Figure S23. ^{1}H NMR (600 MHz) spectrum of (24*R*)-22,23-dihydroxy-ergosta-4,6,8(14)-trien-3-one 23-*β*-D-glucopyranoside (**5**) in methanol-d_4

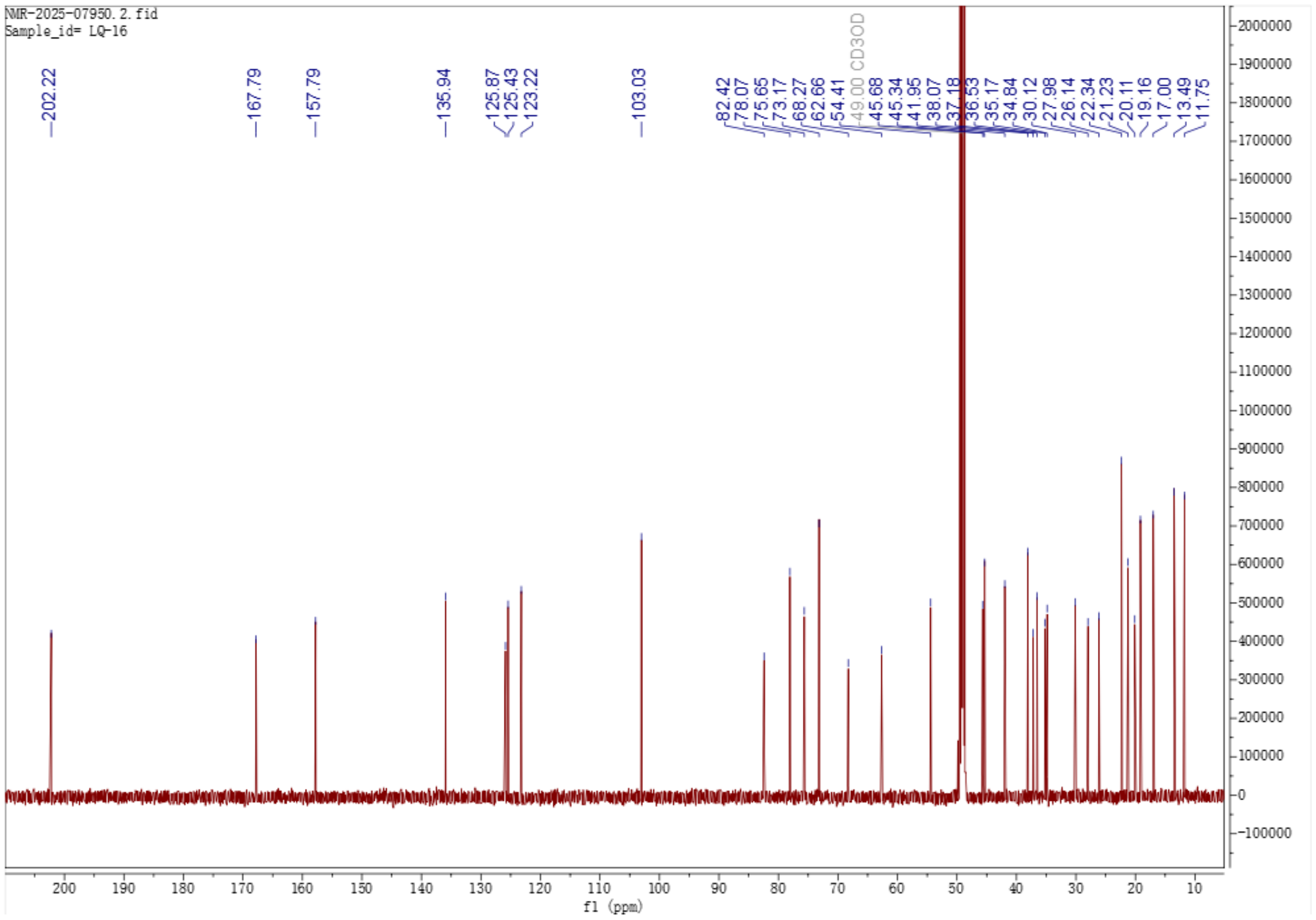

Figure S24. ^{13}C NMR spectra of (24*R*)-22,23-dihydroxy-ergosta-4,6,8(14)-trien-3-one 23-*β*-D-glucopyranoside (**5**) in methanol-d_4

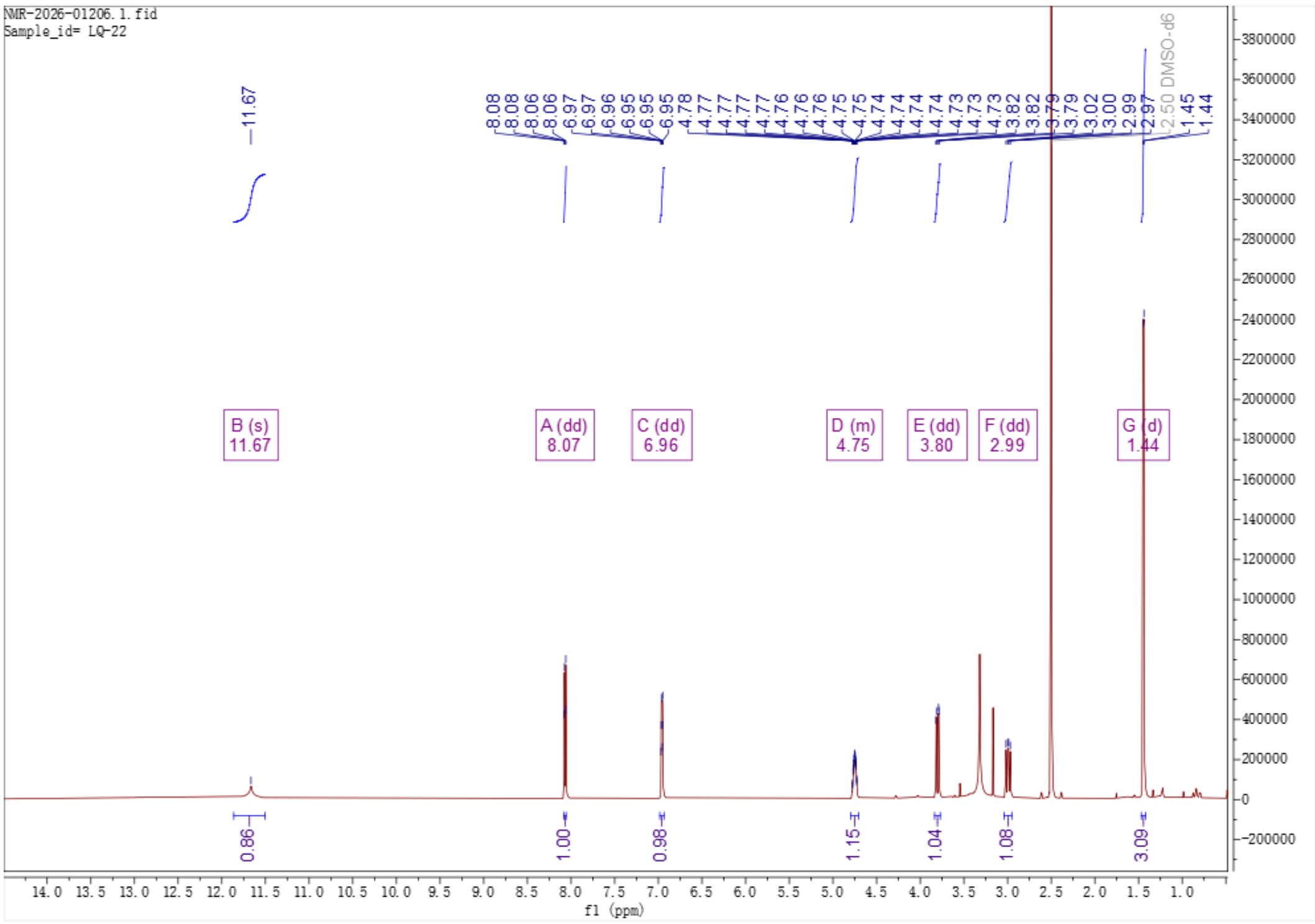

Figure S25. ^{1}H NMR (600 MHz) spectrum of 5-carboxymellein (**6**) in DMSO-d_6

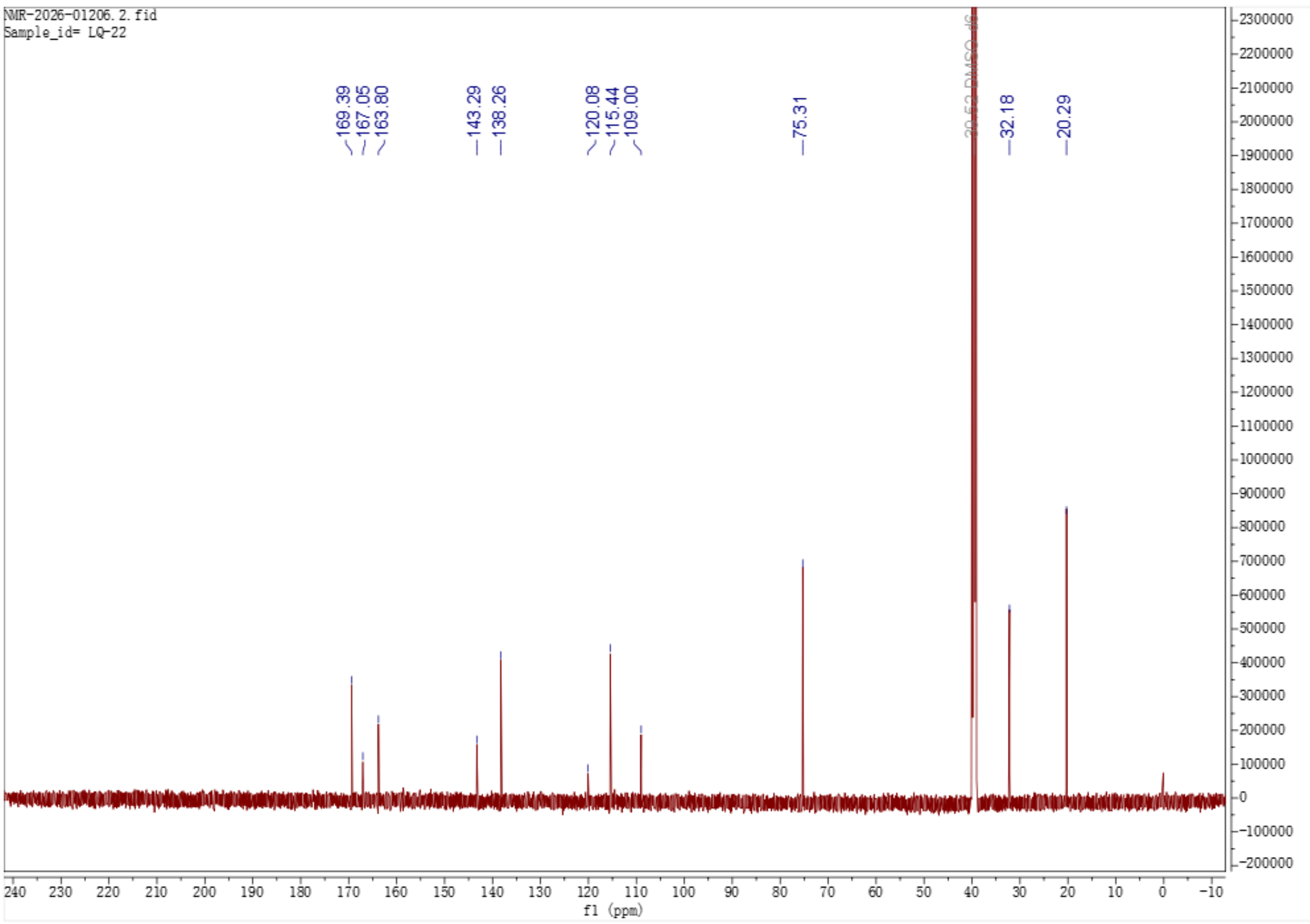

Figure S26. ^{13}C NMR spectra of 5-carboxymellein (**6**) in DMSO-d_6

Computational data for Compounds 1 and 2

Compound 1:

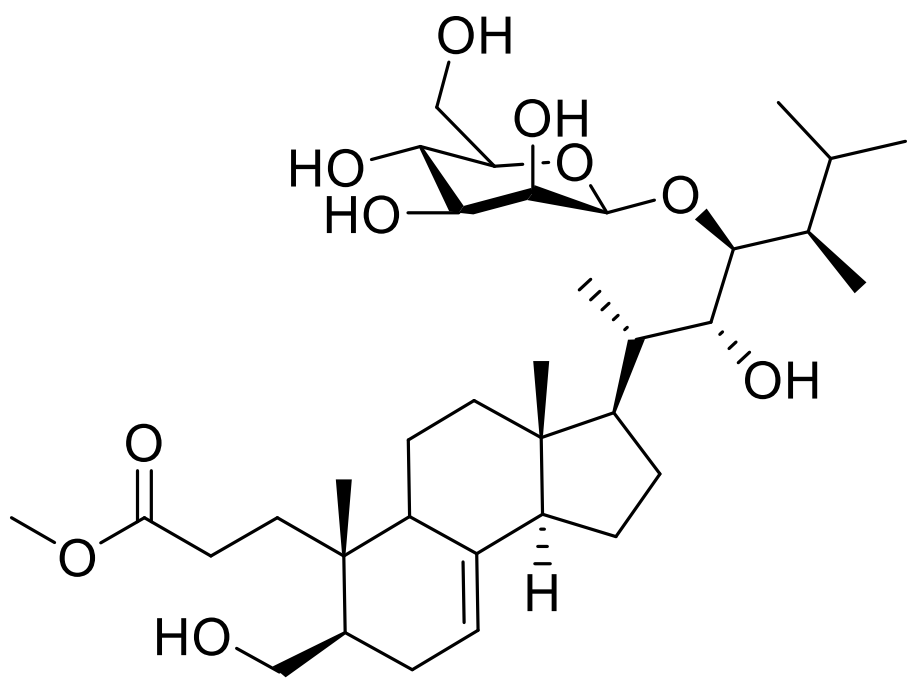

1 (*β*-D-mannopyranose)

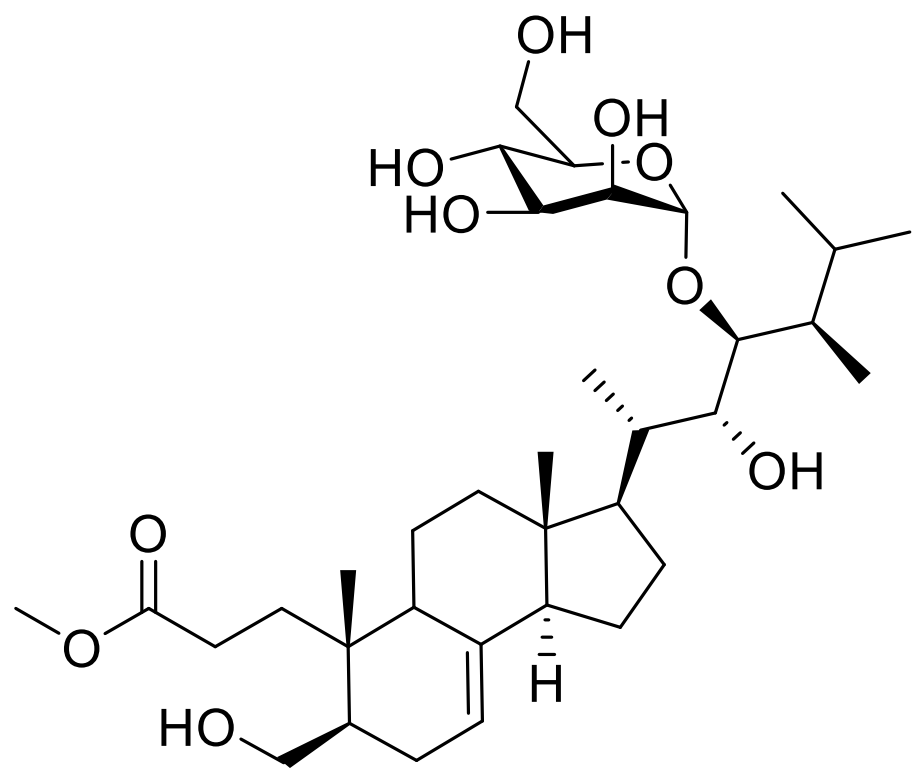

1 (*α*-D-mannopyranose)

Figure S27. Structures of compound **1** used for NMR and ECD (*β*-D-mannopyranose) calculations

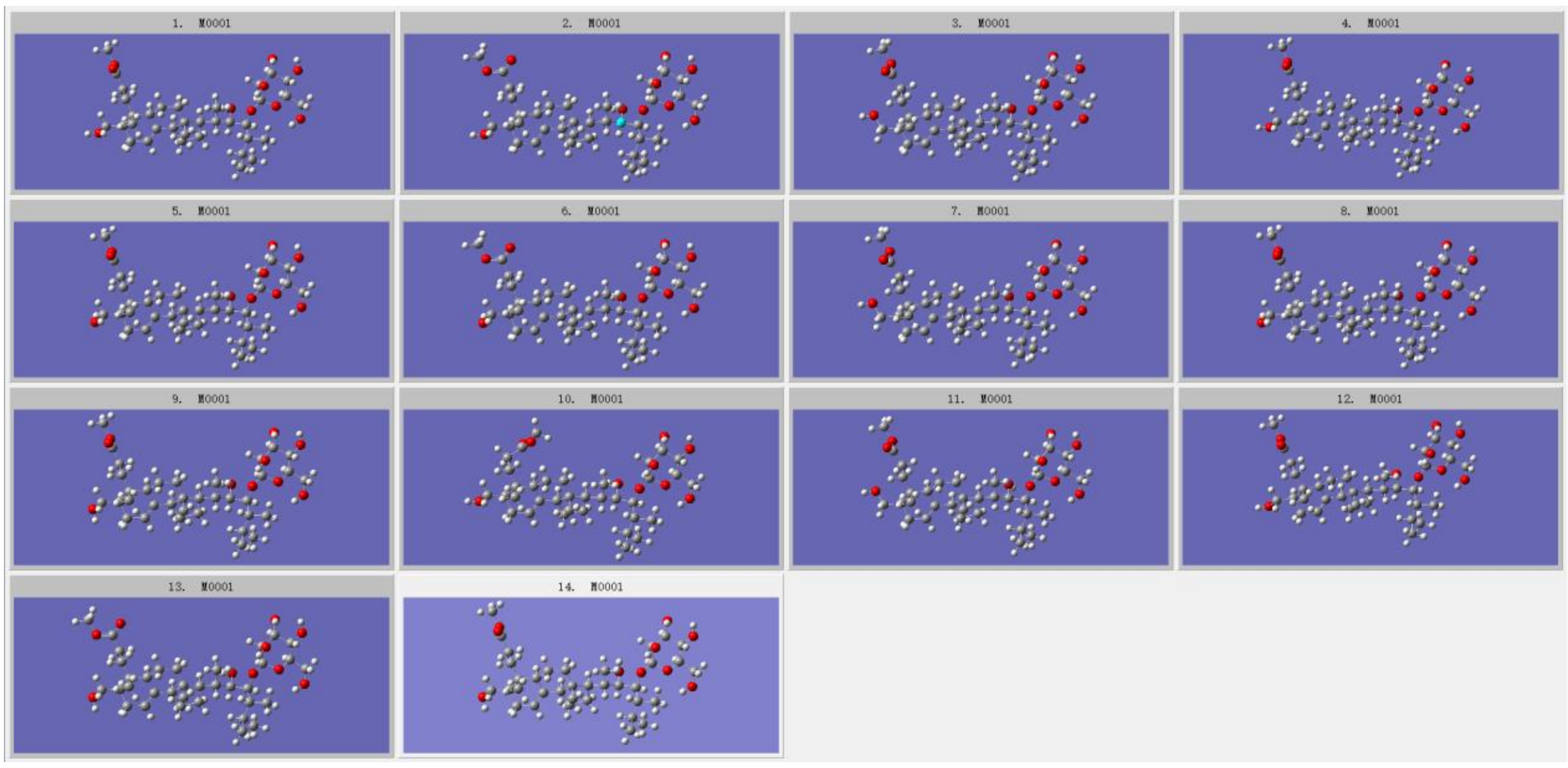

Figure S28. Optimized conformers of compound **1** (*β*-D-mannopyranose)

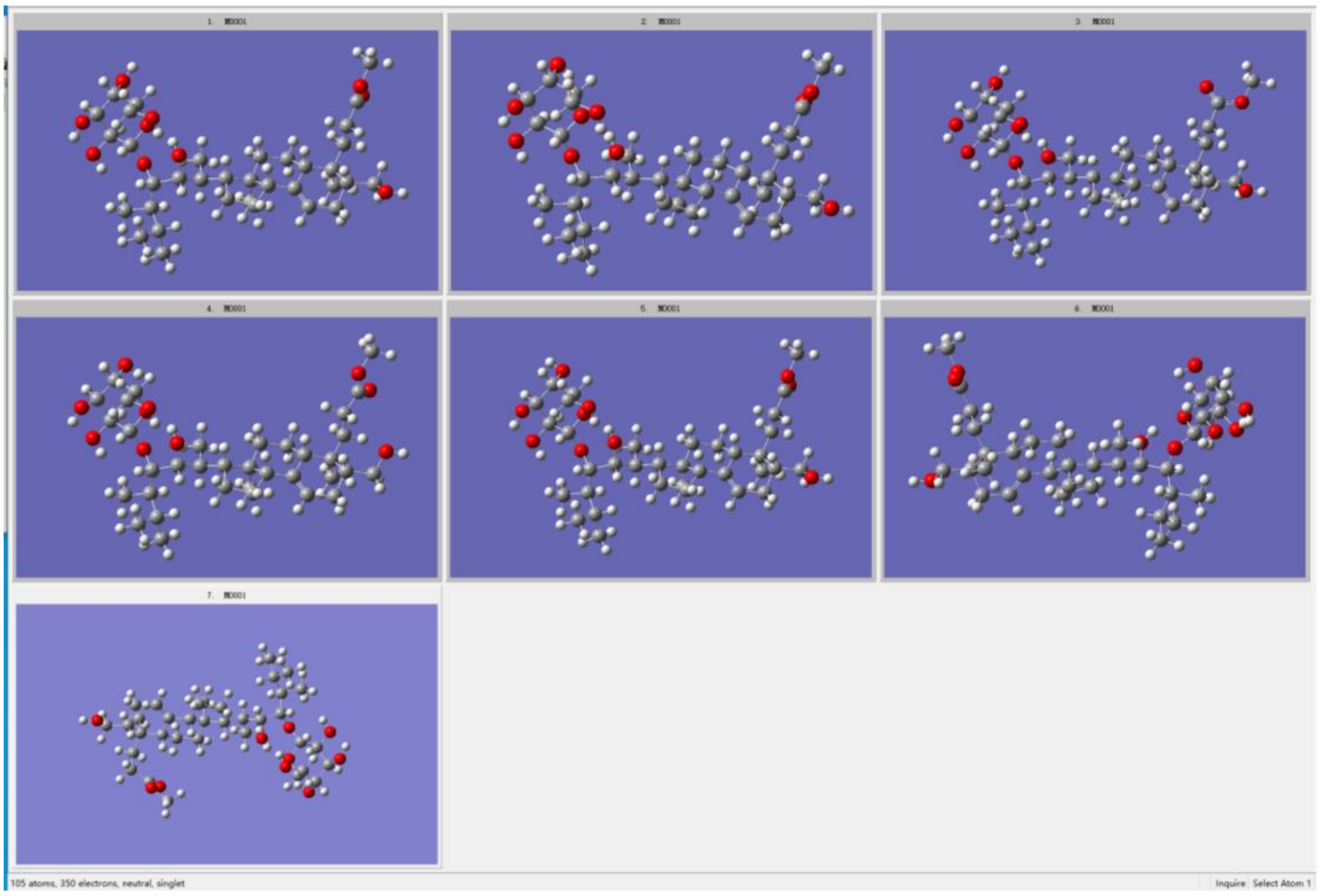

Figure S29. Optimized conformers of compound **1** (α-D-mannopyranose)

Functional		Solvent?		Basis Set		Type of Data	
mPW1PW91		PCM		6-311+G(d,p)		Unscaled Shifts	
		DP4+	100.00%	0.00%	-	-	-
Nuclei	sp2?	xperiments	Isomer 1	Isomer 2	Isomer 3	Isomer 4	Isomer 5
C		11.73	11.5	12.3			
C		12.36	12.6	12.7			
C		13.51	15.7	15.4			
C		18.22	19.5	19.8			
C		21.26	15.6	15.5			
C		22.33	23.5	23.2			
C		23.15	25.5	25.6			
C		23.76	26.4	26.4			
C		26.98	30.1	30.2			
C		28.56	30.7	31.2			
C		28.98	31.0	30.7			
C		30.11	32.83	32.52			
C		33.08	33.52	33.07			
C		37.25	41.75	41.45			
C		37.94	41.99	42.13			
C		41.1	43.53	43.45			
C		41.96	44.38	44.57			
C		43.28	45.26	44.48			
C		44.56	46.48	46.38			
C		44.82	50.09	49.81			
C		52.13	55.25	55.23			
C		54.16	56.51	56.75			
C		56.73	59.79	59.89			
C		62.67	67.96	64.10			
C		63.31	67.57	67.17			
C		68.27	73.35	74.37			
C		73.13	74.83	74.51			
C		73.39	78.30	75.81			
C		75.66	79.05	77.03			
C		78.06	79.36	88.33			
C		82.31	88.54	98.92			
C		103.2	104.78	103.52			
C	x	118.05	125.48	125.50			
C	x	140.24	149.69	149.88			
C	x	176.49	184.95	184.86			

Figure S30. DP4+ analysis of the calculated ^{13}C NMR data of compound **1** (*β*-D-mannopyranose)

Table S1 The calculated ^{13}C NMR data of compound **1** (*β*-D-mannopyranose)

No	Conforme 1	Boltzmann distribution	Conforme 2	Boltzmann distribution	Conforme 3	Boltzmann distribution	Conforme 4	Boltzmann distribution	Conforme 5	Boltzmann distribution	Conforme 6	Boltzmann distribution	Conforme 7	Boltzmann distribution
10-C	43.5688	0.1383625	43.5158	0.0652264	43.4152	0.0854518	43.6093	0.0732087	43.5571	0.1186641	43.4944	0.0714468	43.4264	0.0328358
11-C	25.6244	0.1383625	25.656	0.0652264	25.2056	0.0854518	25.5392	0.0732087	25.4873	0.1186641	25.493	0.0714468	25.0743	0.0328358
12-C	26.3687	0.1383625	26.3699	0.0652264	26.4978	0.0854518	26.2191	0.0732087	26.3567	0.1186641	26.3636	0.0714468	26.4169	0.0328358
13-C	30.6266	0.1383625	30.6228	0.0652264	30.6354	0.0854518	30.9202	0.0732087	30.6033	0.1186641	30.6017	0.0714468	30.8977	0.0328358
14-C	56.44	0.1383625	56.4479	0.0652264	56.3864	0.0854518	56.7456	0.0732087	56.4403	0.1186641	56.4503	0.0714468	56.6923	0.0328358
15-C	42.2017	0.1383625	42.18	0.0652264	42.2031	0.0854518	41.3211	0.0732087	42.1746	0.1186641	42.152	0.0714468	41.3193	0.0328358
16-C	78.3783	0.1383625	78.4183	0.0652264	78.3866	0.0854518	78.0957	0.0732087	78.3739	0.1186641	78.4212	0.0714468	78.1094	0.0328358
17-C	15.9133	0.1383625	15.95	0.0652264	15.9347	0.0854518	14.7598	0.0732087	15.916	0.1186641	15.9481	0.0714468	14.815	0.0328358
19-C	88.1881	0.1383625	88.1396	0.0652264	88.1336	0.0854518	89.6768	0.0732087	88.1935	0.1186641	88.113	0.0714468	89.6262	0.0328358
1-C	44.3398	0.1383625	44.2058	0.0652264	45.0318	0.0854518	44.4259	0.0732087	46.8162	0.1186641	46.6224	0.0714468	45.0515	0.0328358
20-C	44.3115	0.1383625	44.3866	0.0652264	44.4027	0.0854518	44.4582	0.0732087	44.3193	0.1186641	44.3863	0.0714468	44.4979	0.0328358
22-C	32.8665	0.1383625	32.8081	0.0652264	32.8307	0.0854518	32.8266	0.0732087	32.8656	0.1186641	32.806	0.0714468	32.7365	0.0328358
23-C	15.5831	0.1383625	15.5286	0.0652264	15.5622	0.0854518	15.6176	0.0732087	15.5828	0.1186641	15.5285	0.0714468	15.6082	0.0328358
24-C	23.4762	0.1383625	23.4014	0.0652264	23.4458	0.0854518	23.4485	0.0732087	23.4731	0.1186641	23.3958	0.0714468	23.3903	0.0328358
25-C	11.404	0.1383625	11.365	0.0652264	11.3997	0.0854518	11.7045	0.0732087	11.392	0.1186641	11.3603	0.0714468	11.7034	0.0328358
26-C	30.4022	0.1383625	33.6306	0.0652264	31.1243	0.0854518	30.349	0.0732087	30.3592	0.1186641	33.4968	0.0714468	31.169	0.0328358
27-C	184.6431	0.1383625	185.9923	0.0652264	185.3849	0.0854518	184.6771	0.0732087	184.5301	0.1186641	185.8941	0.0714468	185.3846	0.0328358
29-C	55.1827	0.1383625	55.767	0.0652264	54.9348	0.0854518	55.1724	0.0732087	55.1979	0.1186641	55.7974	0.0714468	54.9221	0.0328358
2-C	41.3406	0.1383625	42.2684	0.0652264	42.709	0.0854518	41.2643	0.0732087	41.5159	0.1186641	42.4208	0.0714468	42.7178	0.0328358
31-C	19.7438	0.1383625	19.8215	0.0652264	18.6502	0.0854518	19.9169	0.0732087	19.411	0.1186641	19.4963	0.0714468	18.7517	0.0328358
32-C	67.0218	0.1383625	66.8772	0.0652264	68.7773	0.0854518	67.0629	0.0732087	67.8306	0.1186641	67.649	0.0714468	68.806	0.0328358
34-C	12.5167	0.1383625	12.5803	0.0652264	12.4877	0.0854518	12.6556	0.0732087	12.5207	0.1186641	12.5769	0.0714468	12.5696	0.0328358
35-C	104.7233	0.1383625	104.6711	0.0652264	104.6942	0.0854518	105.0154	0.0732087	104.7374	0.1186641	104.6681	0.0714468	104.9351	0.0328358

Continued

37-C	74.8172	0.1383625	74.7952	0.0652264	74.8141	0.0854518	74.8931	0.0732087	74.8136	0.1186641	74.7884	0.0714468	74.8735	0.0328358
38-C	79.0681	0.1383625	79.056	0.0652264	79.0507	0.0854518	78.9934	0.0732087	79.0642	0.1186641	79.0573	0.0714468	79.0046	0.0328358
39-C	73.4281	0.1383625	73.4206	0.0652264	73.4654	0.0854518	73.0603	0.0732087	73.4303	0.1186641	73.4158	0.0714468	73.0566	0.0328358
3-C	32.6641	0.1383625	37.4721	0.0652264	34.1225	0.0854518	32.7103	0.0732087	32.3351	0.1186641	37.1182	0.0714468	34.1645	0.0328358
40-C	79.2653	0.1383625	79.2812	0.0652264	79.2663	0.0854518	79.638	0.0732087	79.2718	0.1186641	79.2832	0.0714468	79.7243	0.0328358
42-C	67.9803	0.1383625	67.9502	0.0652264	67.9302	0.0854518	67.9754	0.0732087	67.9736	0.1186641	67.9476	0.0714468	67.8763	0.0328358
4-C	30.1479	0.1383625	30.0258	0.0652264	31.9388	0.0854518	30.0862	0.0732087	29.9054	0.1186641	29.8084	0.0714468	31.9304	0.0328358
5-C	125.6989	0.1383625	125.5676	0.0652264	125.8722	0.0854518	125.5709	0.0732087	125.6861	0.1186641	125.5419	0.0714468	125.6997	0.0328358
6-C	149.3501	0.1383625	149.4861	0.0652264	150.1398	0.0854518	149.566	0.0732087	149.4273	0.1186641	149.5486	0.0714468	150.3473	0.0328358
7-C	46.3965	0.1383625	46.4174	0.0652264	46.8549	0.0854518	46.3767	0.0732087	46.4609	0.1186641	46.4482	0.0714468	46.8665	0.0328358
8-C	59.8673	0.1383625	59.7281	0.0652264	59.53	0.0854518	59.8714	0.0732087	59.8439	0.1186641	59.7078	0.0714468	59.5358	0.0328358
9-C	50.2199	0.1383625	50.2775	0.0652264	50.1933	0.0854518	49.6902	0.0732087	50.2076	0.1186641	50.2666	0.0714468	49.6425	0.0328358

Continued

No	Conforme 8	Boltzmann distribution	Conforme 9	Boltzmann distribution	Conforme 10	Boltzmann distribution	Conforme 11	Boltzmann distribution	Conforme 12	Boltzmann distribution	Conforme 13	Boltzmann distribution	Conforme 14	Boltzmann distribution
10-C	43.6014	0.0544784	43.485	0.0970333	43.1456	0.0066197	43.5214	0.0311751	43.5693	0.1416244	43.4085	0.0260657	43.5205	0.0578075
11-C	25.4162	0.0544784	25.3522	0.0970333	25.3835	0.0066197	25.3293	0.0311751	25.6238	0.1416244	25.3195	0.0260657	25.2443	0.0578075
12-C	26.2056	0.0544784	26.3613	0.0970333	26.5099	0.0066197	26.451	0.0311751	26.3696	0.1416244	26.3715	0.0260657	26.2185	0.0578075
13-C	30.9019	0.0544784	30.6133	0.0970333	30.3965	0.0066197	30.9176	0.0311751	30.627	0.1416244	30.5985	0.0260657	30.8979	0.0578075
14-C	56.7557	0.0544784	56.4126	0.0970333	56.3236	0.0066197	56.8632	0.0311751	56.4392	0.1416244	56.4194	0.0260657	56.7142	0.0578075
15-C	41.303	0.0544784	42.215	0.0970333	42.2262	0.0066197	41.9071	0.0311751	42.2015	0.1416244	42.1776	0.0260657	41.335	0.0578075
16-C	78.0947	0.0544784	78.363	0.0970333	78.4031	0.0066197	77.7956	0.0311751	78.3774	0.1416244	78.3981	0.0260657	78.0933	0.0578075
17-C	14.7639	0.0544784	15.9176	0.0970333	15.9102	0.0066197	15.8622	0.0311751	15.9131	0.1416244	15.9516	0.0260657	14.7755	0.0578075
19-C	89.6622	0.0544784	88.199	0.0970333	88.3373	0.0066197	89.6319	0.0311751	88.1922	0.1416244	88.1326	0.0260657	89.6813	0.0578075
1-C	46.8919	0.0544784	45.5999	0.0970333	43.8365	0.0066197	45.0307	0.0311751	44.3392	0.1416244	45.4523	0.0260657	45.6758	0.0578075
20-C	44.4648	0.0544784	44.3241	0.0970333	44.2774	0.0066197	44.8281	0.0311751	44.3109	0.1416244	44.3885	0.0260657	44.474	0.0578075
22-C	32.825	0.0544784	32.8614	0.0970333	32.8793	0.0066197	32.5973	0.0311751	32.8664	0.1416244	32.7954	0.0260657	32.8176	0.0578075
23-C	15.6151	0.0544784	15.5745	0.0970333	15.5983	0.0066197	15.5406	0.0311751	15.5828	0.1416244	15.5237	0.0260657	15.6044	0.0578075
24-C	23.4429	0.0544784	23.4694	0.0970333	23.4615	0.0066197	23.4949	0.0311751	23.4758	0.1416244	23.3856	0.0260657	23.4345	0.0578075
25-C	11.6989	0.0544784	11.3899	0.0970333	11.3919	0.0066197	11.3863	0.0311751	11.4025	0.1416244	11.3584	0.0260657	11.6898	0.0578075
26-C	30.3097	0.0544784	30.2792	0.0970333	31.055	0.0066197	31.1318	0.0311751	30.4013	0.1416244	33.3677	0.0260657	30.2119	0.0578075
27-C	184.5713	0.0544784	184.5982	0.0970333	184.8587	0.0066197	185.4227	0.0311751	184.6438	0.1416244	185.9171	0.0260657	184.6412	0.0578075
29-C	55.1924	0.0544784	55.2043	0.0970333	55.6204	0.0066197	54.9202	0.0311751	55.1827	0.1416244	55.813	0.0260657	55.1955	0.0578075
2-C	41.4321	0.0544784	41.4077	0.0970333	42.9243	0.0066197	42.7105	0.0311751	41.3411	0.1416244	42.3322	0.0260657	41.33	0.0578075
31-C	19.6089	0.0544784	19.3074	0.0970333	19.4408	0.0066197	18.6721	0.0311751	19.7421	0.1416244	19.3621	0.0260657	19.5063	0.0578075
32-C	67.8635	0.0544784	67.6412	0.0970333	66.8493	0.0066197	68.7998	0.0311751	67.02	0.1416244	67.46	0.0260657	67.6765	0.0578075
34-C	12.6493	0.0544784	12.5094	0.0970333	12.6472	0.0066197	12.5811	0.0311751	12.5174	0.1416244	12.5708	0.0260657	12.6353	0.0578075

Continued

35-C	105.0179	0.0544784	104.7236	0.0970333	104.7018	0.0066197	105.0128	0.0311751	104.726	0.1416244	104.6607	0.0260657	105.007	0.0578075
37-C	74.8915	0.0544784	74.8132	0.0970333	74.7583	0.0066197	74.7954	0.0311751	74.8155	0.1416244	74.7862	0.0260657	74.8887	0.0578075
38-C	78.993	0.0544784	79.0611	0.0970333	79.122	0.0066197	79.0564	0.0311751	79.0683	0.1416244	79.053	0.0260657	78.989	0.0578075
39-C	73.0622	0.0544784	73.4321	0.0970333	73.2655	0.0066197	73.4216	0.0311751	73.4274	0.1416244	73.4188	0.0260657	73.0626	0.0578075
3-C	32.4044	0.0544784	32.2782	0.0970333	32.7628	0.0066197	34.1251	0.0311751	32.6648	0.1416244	36.9699	0.0260657	32.3163	0.0578075
40-C	79.6432	0.0544784	79.27	0.0970333	79.2454	0.0066197	79.2815	0.0311751	79.269	0.1416244	79.2809	0.0260657	79.651	0.0578075
42-C	67.9709	0.0544784	67.971	0.0970333	68.0874	0.0066197	67.9679	0.0311751	67.9784	0.1416244	67.9378	0.0260657	67.9606	0.0578075
4-C	29.8126	0.0544784	28.8043	0.0970333	29.5362	0.0066197	31.9323	0.0311751	30.1466	0.1416244	28.6261	0.0260657	28.7446	0.0578075
5-C	125.5519	0.0544784	124.6959	0.0970333	125.9344	0.0066197	125.5212	0.0311751	125.6995	0.1416244	124.59	0.0260657	124.5677	0.0578075
6-C	149.649	0.0544784	150.0213	0.0970333	149.8179	0.0066197	150.4823	0.0311751	149.3496	0.1416244	150.1342	0.0260657	150.2324	0.0578075
7-C	46.4621	0.0544784	46.3572	0.0970333	47.6508	0.0066197	46.9464	0.0311751	46.3948	0.1416244	46.2932	0.0260657	46.3225	0.0578075
8-C	59.8578	0.0544784	59.8422	0.0970333	59.6536	0.0066197	59.6202	0.0311751	59.8677	0.1416244	59.6859	0.0260657	59.8353	0.0578075
9-C	49.6939	0.0544784	50.2167	0.0970333	50.2905	0.0066197	49.78	0.0311751	50.2189	0.1416244	50.2539	0.0260657	49.6917	0.0578075

Table S2 The calculated ^{13}C NMR data of compound **1** (α-D-mannopyranose)

No	Conforme 1	Boltzmann distribution	Conforme 2	Boltzmann distribution	Conforme 3	Boltzmann distribution	Conforme 4	Boltzmann distribution	Conforme 5	Boltzmann distribution	Conforme 6	Boltzmann distribution	Conforme 7	Boltzmann distribution
10-C	43.5688	0.1383625	43.5158	0.0652264	43.4152	0.0854518	43.6093	0.0732087	43.5571	0.1186641	43.4944	0.0714468	43.4264	0.0328358
11-C	25.7271	0.1317624	25.6625	0.1717077	25.2109	0.1250984	25.1472	0.0222332	25.7163	0.4006678	25.6256	0.1434138	25.4334	0.0051167
12-C	26.4215	0.1317624	26.4184	0.1717077	26.3869	0.1250984	26.4673	0.0222332	26.4289	0.4006678	26.2781	0.1434138	26.408	0.0051167
13-C	31.2799	0.1317624	31.2375	0.1717077	31.1463	0.1250984	31.1341	0.0222332	31.2852	0.4006678	31.0869	0.1434138	31.1665	0.0051167
14-C	56.7172	0.1317624	56.6979	0.1717077	56.7135	0.1250984	56.6552	0.0222332	56.7121	0.4006678	57.0135	0.1434138	56.6031	0.0051167
15-C	42.0551	0.1317624	41.993	0.1717077	41.9216	0.1250984	41.87	0.0222332	42.0624	0.4006678	42.7765	0.1434138	41.8277	0.0051167
16-C	76.1095	0.1317624	76.1717	0.1717077	75.9611	0.1250984	76.1401	0.0222332	76.0753	0.4006678	74.1961	0.1434138	76.1839	0.0051167
17-C	15.3496	0.1317624	15.3447	0.1717077	15.4964	0.1250984	15.4862	0.0222332	15.3432	0.4006678	15.9096	0.1434138	15.3592	0.0051167
19-C	99.4844	0.1317624	99.716	0.1717077	99.6961	0.1250984	99.8101	0.0222332	99.4705	0.4006678	95.0712	0.1434138	99.5841	0.0051167
1-C	44.3937	0.1317624	44.3791	0.1717077	44.4394	0.1250984	44.4416	0.0222332	44.3906	0.4006678	45.5987	0.1434138	44.3843	0.0051167
20-C	44.5159	0.1317624	44.499	0.1717077	44.299	0.1250984	44.8487	0.0222332	44.511	0.4006678	44.4746	0.1434138	43.9403	0.0051167
22-C	32.3656	0.1317624	32.3639	0.1717077	32.5663	0.1250984	32.4475	0.0222332	32.3641	0.4006678	33.29	0.1434138	32.5122	0.0051167
23-C	15.5394	0.1317624	15.5264	0.1717077	15.5349	0.1250984	15.5441	0.0222332	15.5381	0.4006678	15.5719	0.1434138	15.6145	0.0051167
24-C	23.2072	0.1317624	23.2079	0.1717077	23.2716	0.1250984	23.2337	0.0222332	23.2061	0.4006678	23.09	0.1434138	23.1434	0.0051167
25-C	12.568	0.1317624	12.5202	0.1717077	12.6563	0.1250984	12.5741	0.0222332	12.5879	0.4006678	10.9214	0.1434138	12.5601	0.0051167
26-C	19.7772	0.1317624	19.7539	0.1717077	19.7745	0.1250984	18.6224	0.0222332	19.775	0.4006678	19.8949	0.1434138	19.4993	0.0051167
27-C	67.1663	0.1317624	67.1598	0.1717077	67.0223	0.1250984	68.8508	0.0222332	67.1652	0.4006678	67.0902	0.1434138	66.7501	0.0051167
29-C	12.7099	0.1317624	12.7066	0.1717077	12.7509	0.1250984	12.6583	0.0222332	12.7133	0.4006678	12.6566	0.1434138	12.7866	0.0051167
2-C	41.293	0.1317624	41.2962	0.1717077	42.2021	0.1250984	42.6974	0.0222332	41.2988	0.4006678	41.2731	0.1434138	42.9557	0.0051167
31-C	103.6125	0.1317624	103.7408	0.1717077	103.0602	0.1250984	103.3598	0.0222332	103.4077	0.4006678	103.9177	0.1434138	103.5498	0.0051167
32-C	74.7192	0.1317624	74.5736	0.1717077	74.6766	0.1250984	74.3994	0.0222332	74.6976	0.4006678	73.6118	0.1434138	74.4849	0.0051167
34-C	76.29	0.1317624	77.2496	0.1717077	76.0375	0.1250984	77.0585	0.0222332	77.8225	0.4006678	76.062	0.1434138	77.1618	0.0051167
35-C	74.1734	0.1317624	74.0741	0.1717077	74.0365	0.1250984	73.9681	0.0222332	73.8847	0.4006678	76.6543	0.1434138	74.0078	0.0051167

Continued

37-C	63.022	0.1317624	62.7983	0.1717077	62.8822	0.1250984	62.7231	0.0222332	63.3388	0.4006678	70.1191	0.1434138	62.8539	0.0051167
38-C	30.129	0.1317624	30.1382	0.1717077	30.1015	0.1250984	32.0388	0.0222332	30.1308	0.4006678	30.0817	0.1434138	29.5083	0.0051167
39-C	32.4641	0.1317624	32.4576	0.1717077	36.8493	0.1250984	34.2609	0.0222332	32.4668	0.4006678	32.5764	0.1434138	33.1091	0.0051167
3-C	30.3138	0.1317624	30.2928	0.1717077	33.3837	0.1250984	31.171	0.0222332	30.3106	0.4006678	30.3212	0.1434138	31.1757	0.0051167
40-C	184.6861	0.1317624	184.6883	0.1717077	185.9425	0.1250984	185.3971	0.0222332	184.6907	0.4006678	184.6918	0.1434138	184.9279	0.0051167
42-C	55.1561	0.1317624	55.16	0.1717077	55.7833	0.1250984	54.9076	0.0222332	55.1596	0.4006678	55.1629	0.1434138	55.6435	0.0051167
4-C	125.4907	0.1317624	125.5304	0.1717077	125.5746	0.1250984	125.6795	0.0222332	125.487	0.4006678	125.4185	0.1434138	125.3052	0.0051167
5-C	149.9138	0.1317624	149.8847	0.1717077	149.6177	0.1250984	150.6544	0.0222332	149.9249	0.4006678	149.8023	0.1434138	150.6781	0.0051167
6-C	46.4373	0.1317624	46.4145	0.1717077	45.9166	0.1250984	46.8977	0.0222332	46.4378	0.4006678	46.3834	0.1434138	47.9693	0.0051167
7-C	59.9042	0.1317624	59.8784	0.1717077	59.8808	0.1250984	59.6158	0.0222332	59.8989	0.4006678	59.9513	0.1434138	59.8422	0.0051167
8-C	49.8266	0.1317624	49.8084	0.1717077	49.7283	0.1250984	49.6929	0.0222332	49.8287	0.4006678	49.8156	0.1434138	49.7189	0.0051167
9-C	43.3969	0.1317624	43.4008	0.1717077	43.4757	0.1250984	43.4766	0.0222332	43.3962	0.4006678	43.6685	0.1434138	43.2491	0.0051167

Compound 2:

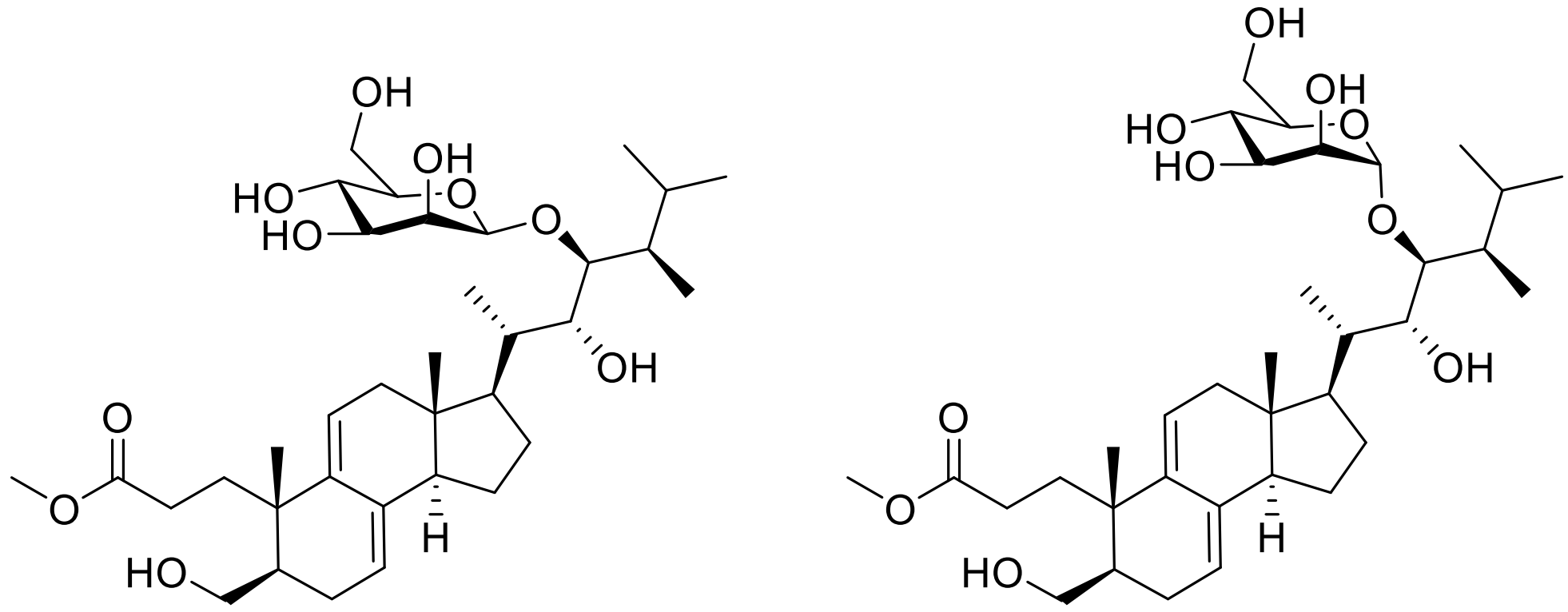

2 (*β*-D-mannopyranose) **2** (*α*-D-mannopyranose)

Figure S31. Structures of **c**ompound **2** used for NMR and ECD (*β*-D-mannopyranose) calculations

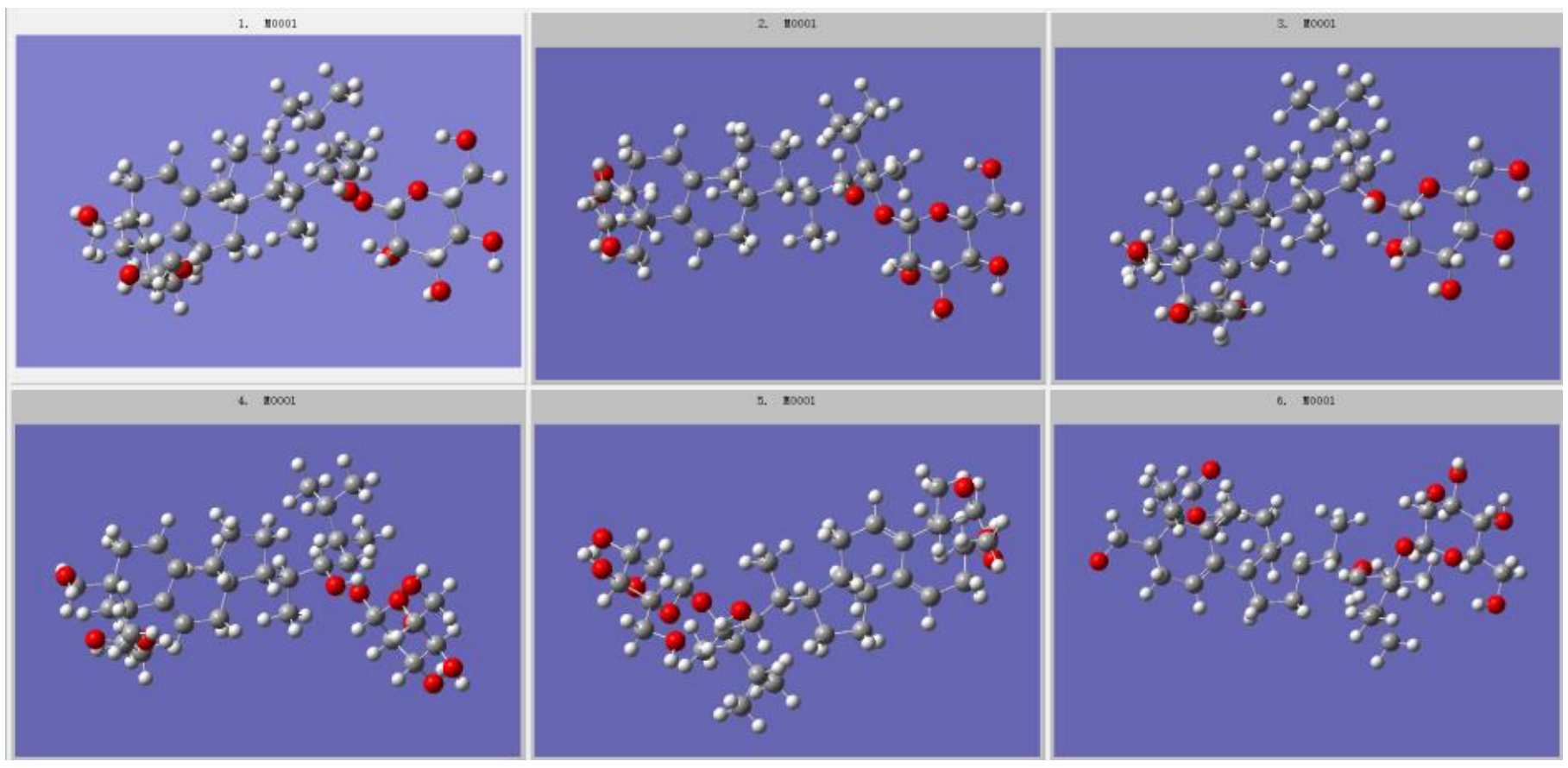

Figure S32. Optimized conformers of compound **2** (*β*-D-mannopyranose)

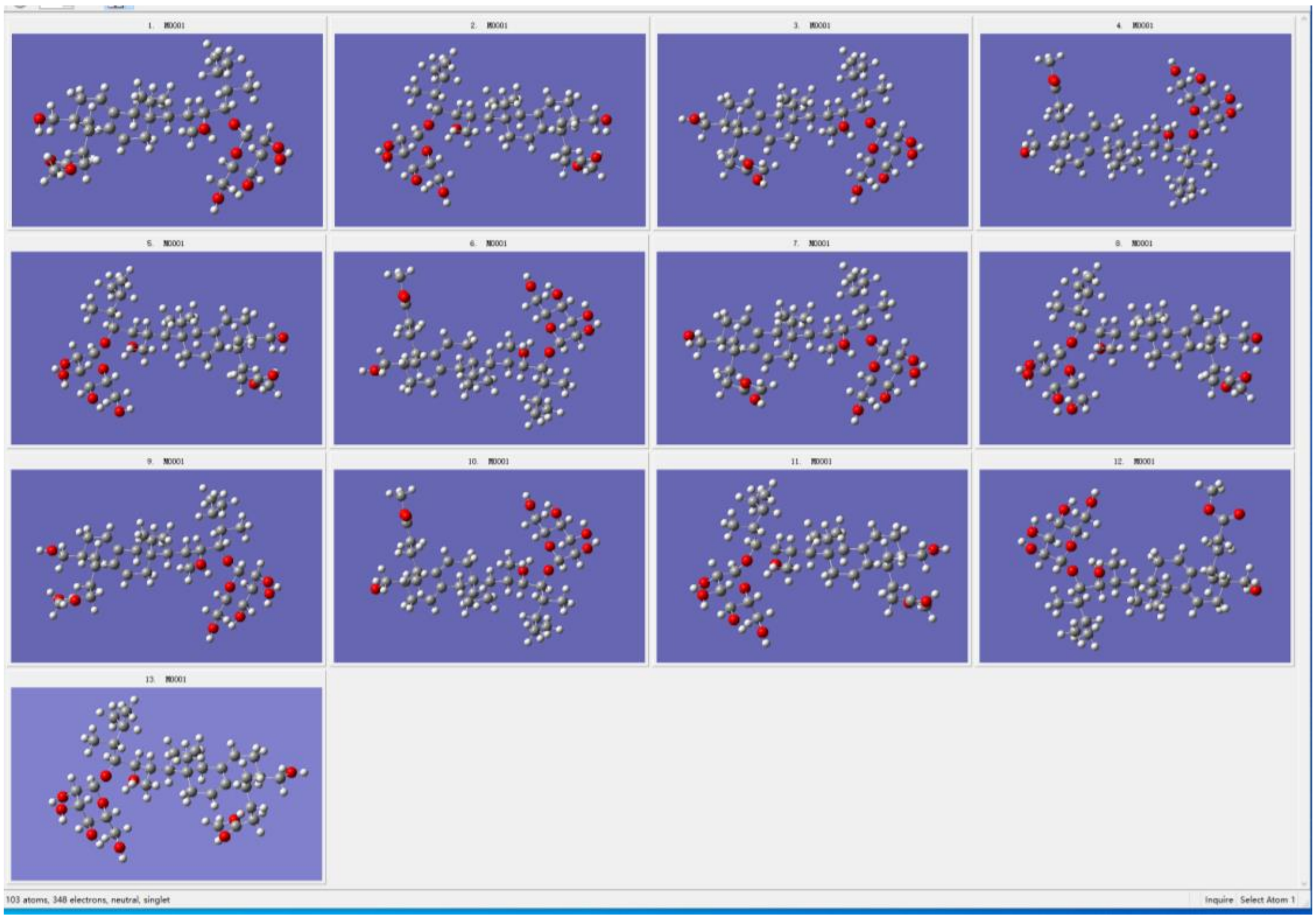

Figure S33. Optimized conformers of compound **2** (α-D-mannopyranose)

Functional		Solvent?		Basis Set		Type of Data	
mPW1PW91		PCM		6-311+G(d,p)		Unscaled Shifts	
		DP4+	100.00%	0.00%	-	-	-
Nuclei	sp2?	Experimental	Isomer 1	Isomer 2	Isomer 3	Isomer 4	Isomer 5
C		11.7	15.0	12.2			
C		12.7	25.6	12.4			
C		13	17.4	15.9			
C		21.2	21.5	15.5			
C		22.1	22.4	26.8			
C		22.3	23.1	23.2			
C		23.6	28.8	26.4			
C		25.4	27.6	29.6			
C		29	33.3	31.3			
C		30	31.7	31.5			
C		30.1	33.8	32.6			
C		35.3	35.51	32.89			
C		37.8	42.70	41.54			
C		38.9	43.20	45.04			
C		42	42.59	44.91			
C		44	41.01	46.62			
C		44.2	49.81	47.97			
C		46.4	50.62	45.05			
C		52.1	55.06	55.27			
C		52.6	55.19	55.81			
C		54.4	59.56	57.27			
C		62.7	65.67	67.73			
C		62.7	65.85	71.12			
C		68.3	71.67	76.85			
C		73.1	75.70	73.95			
C		73.4	73.69	75.85			
C		75.6	78.97	75.39			
C		78	78.51	73.63			
C		82.4	85.65	101.07			
C		103	100.12	109.94			
C	x	118.8	134.32	129.86			
C	x	122.7	125.68	128.88			
C	x	136.7	144.49	145.69			
C	x	139.5	144.40	147.70			
C	x	176.5	184.90	184.96			

Figure S34. DP4+ analysis of the calculated ^{13}C NMR data of compound **2** (*β*-D-mannopyranose)

Table S3 The calculated ^{13}C NMR data of compound **2** (*β*-D-mannopyranose)

No	Conforme 1	Boltzmann distribution	Conforme 2	Boltzmann distribution	Conforme 3	Boltzmann distribution	Conforme 4	Boltzmann distribution	Conforme 5	Boltzmann distribution	Conforme 6	Boltzmann distribution
10-C	145.944	0.0391683	143.574	0.9435270	145.9454	0.0085764	146.285	0.0000882	143.8387	0.0042675	145.6606	0.0043727
11-C	148.2089	0.0391683	149.5222	0.9435270	148.2699	0.0085764	148.0663	0.0000882	149.7563	0.0042675	146.7102	0.0043727
12-C	55.0713	0.0391683	55.5991	0.9435270	55.1911	0.0085764	55.4443	0.0000882	55.8338	0.0042675	55.1701	0.0043727
13-C	48.7216	0.0391683	49.3001	0.9435270	48.6368	0.0085764	48.1675	0.0000882	49.114	0.0042675	48.8653	0.0043727
14-C	46.6587	0.0391683	46.8193	0.9435270	46.7259	0.0085764	46.8372	0.0000882	46.8331	0.0042675	46.076	0.0043727
15-C	131.4986	0.0391683	130.2162	0.9435270	131.4727	0.0085764	131.5394	0.0000882	130.4693	0.0042675	131.596	0.0043727
16-C	26.2997	0.0391683	26.0285	0.9435270	26.2487	0.0085764	26.0827	0.0000882	26.1666	0.0042675	26.4958	0.0043727
17-C	30.9617	0.0391683	31.2704	0.9435270	31.0117	0.0085764	31.1987	0.0000882	31.1102	0.0042675	30.7487	0.0043727
18-C	56.7183	0.0391683	56.8185	0.9435270	56.7512	0.0085764	57.0906	0.0000882	57.0333	0.0042675	56.5925	0.0043727
19-C	41.7967	0.0391683	41.7704	0.9435270	41.7014	0.0085764	40.8701	0.0000882	41.0899	0.0042675	41.8407	0.0043727
1-C	33.6486	0.0391683	35.0562	0.9435270	33.662	0.0085764	33.8082	0.0000882	35.4303	0.0042675	32.6917	0.0043727
20-C	78.6625	0.0391683	78.2413	0.9435270	78.5252	0.0085764	78.589	0.0000882	78.5442	0.0042675	78.3225	0.0043727
21-C	15.478	0.0391683	15.6022	0.9435270	15.4794	0.0085764	14.6428	0.0000882	14.6109	0.0042675	15.5107	0.0043727
23-C	87.5564	0.0391683	88.6436	0.9435270	87.7061	0.0085764	84.1109	0.0000882	84.2492	0.0042675	88.5869	0.0043727
24-C	44.2911	0.0391683	44.1873	0.9435270	44.1824	0.0085764	44.1496	0.0000882	44.1863	0.0042675	44.3659	0.0043727
26-C	104.2375	0.0391683	104.8473	0.9435270	104.5306	0.0085764	101.2853	0.0000882	101.179	0.0042675	104.7469	0.0043727
28-C	79.2605	0.0391683	79.2168	0.9435270	75.3956	0.0085764	79.2982	0.0000882	79.2716	0.0042675	79.2215	0.0043727
29-C	73.4765	0.0391683	73.5797	0.9435270	79.9024	0.0085764	73.1013	0.0000882	73.105	0.0042675	73.2233	0.0043727
2-C	187.6506	0.0391683	185.6852	0.9435270	187.5388	0.0085764	187.2877	0.0000882	185.6042	0.0042675	185.0177	0.0043727
30-C	79.2366	0.0391683	79.0276	0.9435270	78.4393	0.0085764	79.7473	0.0000882	79.8256	0.0042675	79.0815	0.0043727
31-C	74.7983	0.0391683	74.9584	0.9435270	74.5181	0.0085764	74.7992	0.0000882	74.8779	0.0042675	74.7541	0.0043727
32-C	67.9255	0.0391683	67.8395	0.9435270	70.719	0.0085764	67.1833	0.0000882	67.2098	0.0042675	68.1057	0.0043727
37-C	32.9061	0.0391683	32.6996	0.9435270	32.8812	0.0085764	32.5347	0.0000882	32.5609	0.0042675	32.8188	0.0043727
38-C	15.5529	0.0391683	15.5233	0.9435270	15.5131	0.0085764	15.6704	0.0000882	15.8069	0.0042675	15.6216	0.0043727
39-C	23.3524	0.0391683	23.3322	0.9435270	23.2922	0.0085764	23.2854	0.0000882	23.3963	0.0042675	23.4667	0.0043727
40-C	11.336	0.0391683	11.3209	0.9435270	11.1898	0.0085764	12.5728	0.0000882	12.5314	0.0042675	11.3619	0.0043727

continued

42-C	55.9498	0.0391683	55.6465	0.9435270	55.9387	0.0085764	55.879	0.0000882	55.6557	0.0042675	55.2275	0.0043727
44-C	26.5263	0.0391683	24.1141	0.9435270	26.4795	0.0085764	26.516	0.0000882	23.9297	0.0042675	27.4919	0.0043727
45-C	12.413	0.0391683	13.0451	0.9435270	12.4095	0.0085764	12.4458	0.0000882	13.0098	0.0042675	12.4298	0.0043727
4-C	69.7797	0.0391683	65.6834	0.9435270	69.8747	0.0085764	69.7014	0.0000882	65.7278	0.0042675	67.7576	0.0043727
5-C	47.0497	0.0391683	45.0246	0.9435270	46.9863	0.0085764	47.5219	0.0000882	45.0239	0.0042675	45.0268	0.0043727
6-C	46.8625	0.0391683	43.7325	0.9435270	46.8817	0.0085764	46.6526	0.0000882	43.5524	0.0042675	45.6589	0.0043727
7-C	32.3039	0.0391683	43.178	0.9435270	32.2081	0.0085764	32.5286	0.0000882	43.7646	0.0042675	35.0528	0.0043727
8-C	30.9126	0.0391683	27.4297	0.9435270	30.8931	0.0085764	30.9112	0.0000882	26.7563	0.0042675	29.2995	0.0043727
9-C	129.245	0.0391683	126.3724	0.9435270	129.0895	0.0085764	128.9263	0.0000882	125.9823	0.0042675	128.8382	0.0043727

Table S4 The calculated ^{13}C NMR data of compound **2** (*α*-D-mannopyranose)

No	Conforme 1	Boltzmann distribution	Conforme 2	Boltzmann distribution	Conforme 3	Boltzmann distribution	Conforme 4	Boltzmann distribution	Conforme 5	Boltzmann distribution	Conforme 6	Boltzmann distribution	Conforme 7	Boltzmann distribution
10-C	131.6231	0.0082610	131.6268	0.0082348	131.6279	0.0004937	129.7089	0.2612597	131.5426	0.0073759	129.6906	0.2643214	131.5953	0.0004293
11-C	26.2847	0.0082610	26.2851	0.0082348	26.4735	0.0004937	26.4556	0.2612597	26.2783	0.0073759	26.4689	0.2643214	26.4662	0.0004293
12-C	31.2576	0.0082610	31.258	0.0082348	31.1916	0.0004937	31.2816	0.2612597	31.2433	0.0073759	31.2987	0.2643214	31.1723	0.0004293
13-C	57.3331	0.0082610	57.3297	0.0082348	57.1837	0.0004937	57.2779	0.2612597	57.3508	0.0073759	57.2738	0.2643214	57.1953	0.0004293
14-C	41.6462	0.0082610	41.6493	0.0082348	41.7566	0.0004937	41.5082	0.2612597	41.6032	0.0073759	41.525	0.2643214	41.7309	0.0004293
15-C	75.9471	0.0082610	75.9437	0.0082348	75.8413	0.0004937	75.8491	0.2612597	75.8988	0.0073759	75.8566	0.2643214	75.8403	0.0004293
16-C	15.8283	0.0082610	15.8295	0.0082348	15.8303	0.0004937	15.9177	0.2612597	15.8826	0.0073759	15.9159	0.2643214	15.8299	0.0004293
17-C	101.0648	0.0082610	101.0704	0.0082348	101.0031	0.0004937	101.0875	0.2612597	101.0595	0.0073759	101.0891	0.2643214	100.9898	0.0004293
19-C	44.857	0.0082610	44.8603	0.0082348	44.8715	0.0004937	44.9233	0.2612597	44.8606	0.0073759	44.9295	0.2643214	44.8682	0.0004293
1-C	47.3838	0.0082610	47.3787	0.0082348	42.6606	0.0004937	45.6643	0.2612597	47.3727	0.0073759	43.2857	0.2643214	44.9905	0.0004293
20-C	32.7084	0.0082610	32.7078	0.0082348	32.8676	0.0004937	32.6335	0.2612597	32.7072	0.0073759	32.6423	0.2643214	32.8686	0.0004293
22-C	15.5507	0.0082610	15.5501	0.0082348	15.5517	0.0004937	15.5215	0.2612597	15.5426	0.0073759	15.5214	0.2643214	15.5528	0.0004293
23-C	23.2761	0.0082610	23.2771	0.0082348	23.2363	0.0004937	23.1991	0.2612597	23.2706	0.0073759	23.2005	0.2643214	23.2327	0.0004293
24-C	12.2048	0.0082610	12.2059	0.0082348	12.1596	0.0004937	12.1619	0.2612597	12.1973	0.0073759	12.1619	0.2643214	12.1595	0.0004293
25-C	26.5534	0.0082610	26.5527	0.0082348	27.8824	0.0004937	26.7908	0.2612597	26.5507	0.0073759	27.1137	0.2643214	27.5179	0.0004293
26-C	69.508	0.0082610	69.5111	0.0082348	66.9361	0.0004937	67.8797	0.2612597	69.5032	0.0073759	67.0871	0.2643214	67.6549	0.0004293
27-C	12.6328	0.0082610	12.6323	0.0082348	12.629	0.0004937	12.3501	0.2612597	12.6293	0.0073759	12.3564	0.2643214	12.6201	0.0004293
29-C	46.7048	0.0082610	46.7079	0.0082348	45.492	0.0004937	44.778	0.2612597	46.7053	0.0073759	44.6792	0.2643214	45.5452	0.0004293
2-C	110.1422	0.0082610	110.1423	0.0082348	110.0872	0.0004937	109.9218	0.2612597	110.1632	0.0073759	109.9275	0.2643214	110.085	0.0004293
31-C	73.8386	0.0082610	73.8402	0.0082348	73.8509	0.0004937	73.9199	0.2612597	73.9001	0.0073759	73.9165	0.2643214	73.8486	0.0004293
32-C	75.3114	0.0082610	75.3107	0.0082348	75.3493	0.0004937	75.4219	0.2612597	75.2439	0.0073759	75.4209	0.2643214	75.3473	0.0004293
34-C	77.0414	0.0082610	77.0415	0.0082348	77.0301	0.0004937	77.0852	0.2612597	77.0706	0.0073759	77.087	0.2643214	77.0281	0.0004293
35-C	73.6612	0.0082610	73.6658	0.0082348	73.7	0.0004937	73.4201	0.2612597	74.3171	0.0073759	73.4335	0.2643214	73.6931	0.0004293

Continued

37-C	71.403	0.0082610	71.4028	0.0082348	71.3497	0.0004937	71.3578	0.2612597	71.1762	0.0073759	71.3635	0.2643214	71.3469	0.0004293
38-C	31.0364	0.0082610	31.0382	0.0082348	29.418	0.0004937	29.5206	0.2612597	31.0353	0.0073759	29.7307	0.2643214	29.1306	0.0004293
39-C	32.4607	0.0082610	32.4516	0.0082348	35.7028	0.0004937	32.1324	0.2612597	32.4565	0.0073759	32.4652	0.2643214	35.3515	0.0004293
3-C	33.8446	0.0082610	33.8422	0.0082348	32.8855	0.0004937	30.807	0.2612597	33.8392	0.0073759	30.8314	0.2643214	32.8738	0.0004293
40-C	187.5789	0.0082610	187.5773	0.0082348	185.1022	0.0004937	184.5817	0.2612597	187.565	0.0073759	184.6929	0.2643214	184.9884	0.0004293
42-C	55.938	0.0082610	55.9399	0.0082348	55.313	0.0004937	55.1564	0.2612597	55.9422	0.0073759	55.1378	0.2643214	55.3236	0.0004293
4-C	129.1786	0.0082610	129.1787	0.0082348	128.8897	0.0004937	128.832	0.2612597	129.2277	0.0073759	128.9071	0.2643214	128.8033	0.0004293
5-C	146.2054	0.0082610	146.2055	0.0082348	145.8285	0.0004937	145.6838	0.2612597	146.1702	0.0073759	145.5711	0.2643214	145.9317	0.0004293
6-C	148.2881	0.0082610	148.2907	0.0082348	146.7519	0.0004937	147.6948	0.2612597	148.3314	0.0073759	147.696	0.2643214	146.7815	0.0004293
7-C	55.2401	0.0082610	55.2395	0.0082348	55.4429	0.0004937	55.8636	0.2612597	55.2298	0.0073759	55.8647	0.2643214	55.4339	0.0004293
8-C	48.1205	0.0082610	48.1206	0.0082348	48.1069	0.0004937	47.9465	0.2612597	48.1152	0.0073759	47.955	0.2643214	48.1151	0.0004293
9-C	46.6797	0.0082610	46.6793	0.0082348	46.2706	0.0004937	46.6096	0.2612597	46.6744	0.0073759	46.6319	0.2643214	46.2459	0.0004293

Continued

No	Conforme 8	Boltzmann distribution	Conforme 9	Boltzmann distribution	Conforme 10	Boltzmann distribution	Conforme 11	Boltzmann distribution	Conforme 12	Boltzmann distribution	Conforme 13	Boltzmann distribution
10-C	131.5898	0.034773245	131.1853	0.0034078	129.7047	0.2620912	131.1879	0.0033970	129.9513	0.1454441	131.6307	0.0005107
11-C	26.2752	0.034773245	26.429	0.0034078	26.4566	0.2620912	26.4279	0.0033970	26.3556	0.1454441	26.4732	0.0005107
12-C	31.2923	0.034773245	31.2543	0.0034078	31.2838	0.2620912	31.2525	0.0033970	31.4028	0.1454441	31.1922	0.0005107
13-C	57.2297	0.034773245	57.3633	0.0034078	57.276	0.2620912	57.3605	0.0033970	57.217	0.1454441	57.1849	0.0005107
14-C	41.6858	0.034773245	41.6042	0.0034078	41.5073	0.2620912	41.6046	0.0033970	41.604	0.1454441	41.7569	0.0005107
15-C	76.1052	0.034773245	75.9665	0.0034078	75.8488	0.2620912	75.9676	0.0033970	75.7487	0.1454441	75.8399	0.0005107
16-C	15.8121	0.034773245	15.8278	0.0034078	15.9162	0.2620912	15.8297	0.0033970	15.9166	0.1454441	15.8307	0.0005107
17-C	100.9726	0.034773245	101.0472	0.0034078	101.0828	0.2620912	101.0409	0.0033970	101.0354	0.1454441	101.005	0.0005107
19-C	44.8593	0.034773245	44.8816	0.0034078	44.924	0.2620912	44.8813	0.0033970	44.8709	0.1454441	44.8722	0.0005107
1-C	47.4451	0.034773245	43.0664	0.0034078	45.6619	0.2620912	43.0621	0.0033970	45.227	0.1454441	42.6622	0.0005107
20-C	32.6731	0.034773245	32.7574	0.0034078	32.6332	0.2620912	32.7583	0.0033970	32.4997	0.1454441	32.8673	0.0005107
22-C	15.5934	0.034773245	15.5458	0.0034078	15.5199	0.2620912	15.5488	0.0033970	15.5218	0.1454441	15.5505	0.0005107
23-C	23.3242	0.034773245	23.2508	0.0034078	23.1995	0.2620912	23.2505	0.0033970	23.2385	0.1454441	23.2364	0.0005107
24-C	12.2613	0.034773245	12.1796	0.0034078	12.1615	0.2620912	12.1812	0.0033970	12.2037	0.1454441	12.1585	0.0005107
25-C	26.5736	0.034773245	27.1917	0.0034078	26.7892	0.2620912	27.1903	0.0033970	26.6231	0.1454441	27.8839	0.0005107
26-C	69.4376	0.034773245	67.7694	0.0034078	67.8786	0.2620912	67.7727	0.0033970	67.6732	0.1454441	66.9349	0.0005107
27-C	12.5527	0.034773245	12.609	0.0034078	12.3481	0.2620912	12.609	0.0033970	12.3429	0.1454441	12.6293	0.0005107
29-C	46.6763	0.034773245	47.1289	0.0034078	44.7757	0.2620912	47.1274	0.0033970	45.8758	0.1454441	45.4931	0.0005107
2-C	110.2477	0.034773245	110.1869	0.0034078	109.9226	0.2620912	110.1812	0.0033970	109.8783	0.1454441	110.0891	0.0005107
31-C	74.7417	0.034773245	73.835	0.0034078	73.9168	0.2620912	73.8334	0.0033970	73.9406	0.1454441	73.8509	0.0005107
32-C	74.61	0.034773245	75.3169	0.0034078	75.4185	0.2620912	75.3151	0.0033970	75.4173	0.1454441	75.3507	0.0005107
34-C	70.3051	0.034773245	77.0327	0.0034078	77.0857	0.2620912	77.0324	0.0033970	77.0895	0.1454441	77.0307	0.0005107

35-C	79.04	0.034773245	73.7445	0.0034078	73.4214	0.2620912	73.7501	0.0033970	73.424	0.1454441	73.6988	0.0005107
37-C	64.4862	0.034773245	71.3947	0.0034078	71.3585	0.2620912	71.3946	0.0033970	71.347	0.1454441	71.3501	0.0005107
38-C	30.9607	0.034773245	29.5789	0.0034078	29.5224	0.2620912	29.5797	0.0033970	29.1962	0.1454441	29.4147	0.0005107
39-C	32.4768	0.034773245	33.2779	0.0034078	32.1278	0.2620912	33.2794	0.0033970	36.5146	0.1454441	35.7025	0.0005107
3-C	33.7697	0.034773245	33.6615	0.0034078	30.8045	0.2620912	33.6622	0.0033970	33.9266	0.1454441	32.8829	0.0005107
40-C	187.6668	0.034773245	184.9688	0.0034078	184.5814	0.2620912	184.9685	0.0033970	185.7162	0.1454441	185.103	0.0005107
42-C	55.9301	0.034773245	55.5506	0.0034078	55.1577	0.2620912	55.5471	0.0033970	55.6307	0.1454441	55.3148	0.0005107
4-C	129.1796	0.034773245	128.9868	0.0034078	128.8321	0.2620912	128.9878	0.0033970	128.8658	0.1454441	128.8898	0.0005107
5-C	146.2034	0.034773245	146.0213	0.0034078	145.682	0.2620912	146.0259	0.0033970	145.7359	0.1454441	145.8285	0.0005107
6-C	148.318	0.034773245	148.6572	0.0034078	147.6938	0.2620912	148.6599	0.0033970	147.4667	0.1454441	146.7495	0.0005107
7-C	55.1873	0.034773245	55.3149	0.0034078	55.8651	0.2620912	55.3137	0.0033970	55.7836	0.1454441	55.4423	0.0005107
8-C	48.1185	0.034773245	48.041	0.0034078	47.9459	0.2620912	48.0433	0.0033970	48.0229	0.1454441	48.106	0.0005107
9-C	46.6365	0.034773245	46.7075	0.0034078	46.6103	0.2620912	46.7072	0.0033970	46.6415	0.1454441	46.2713	0.0005107

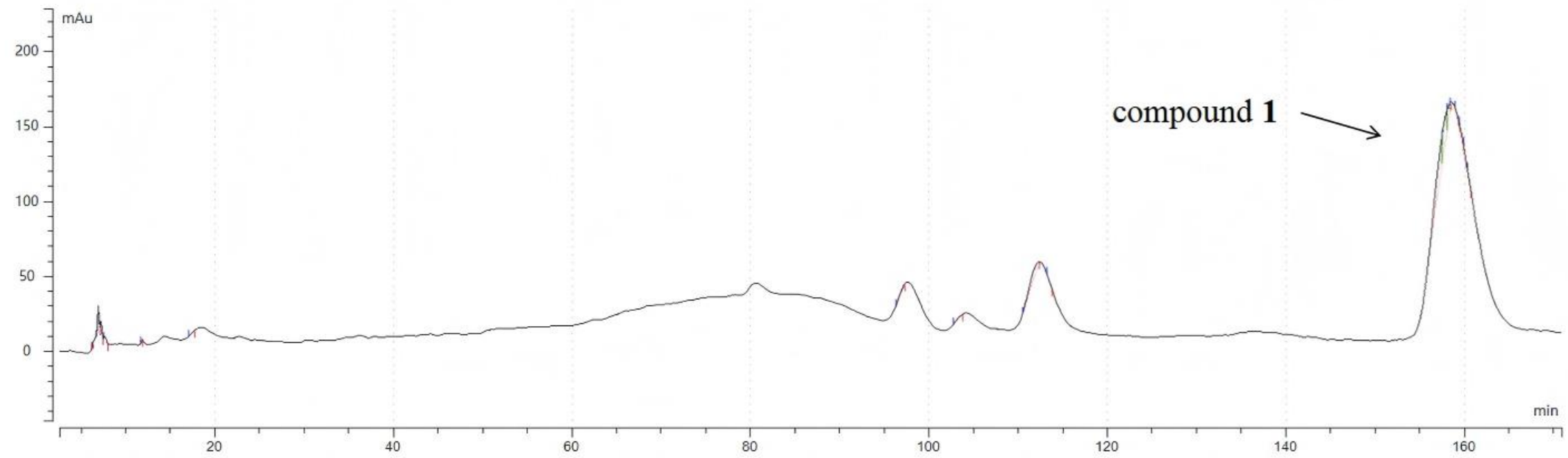

Figure S35. Preparative HPLC chromatogram of mannosylxylarinol A (**1**)

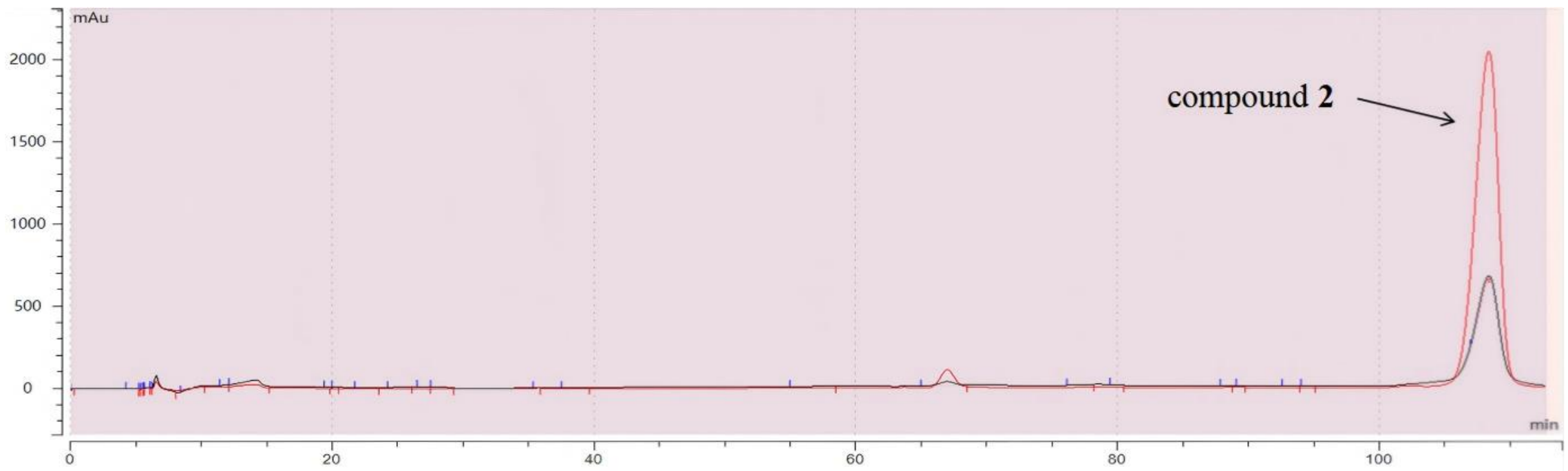

Figure S36. Preparative HPLC chromatogram of mannosylxylarinol B (**2**)

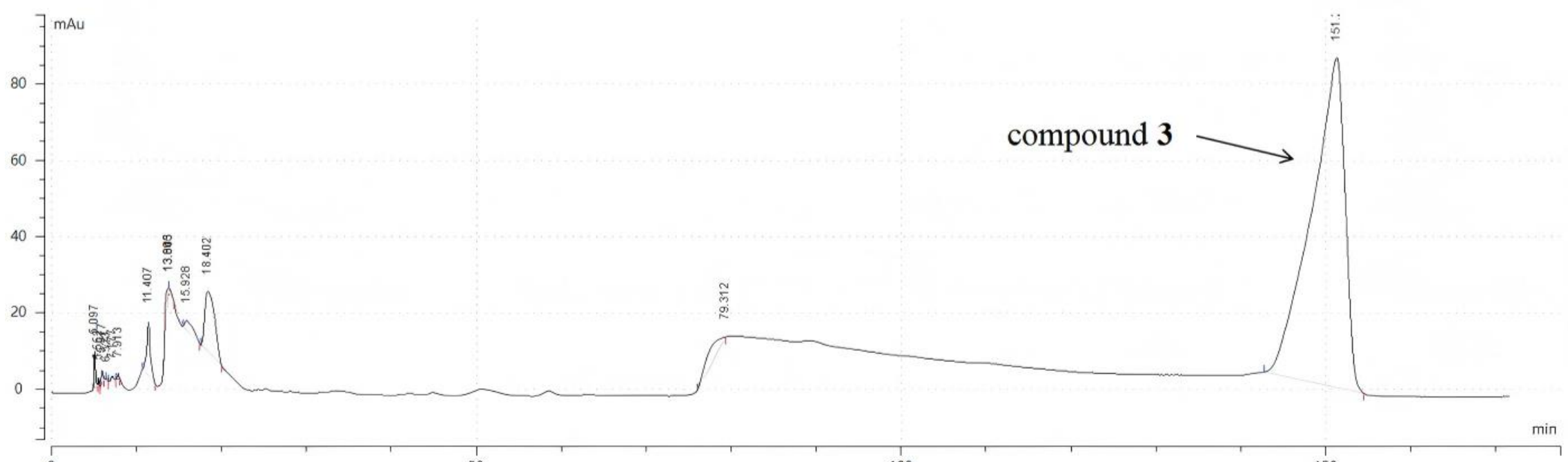

Figure S37. Preparative HPLC chromatogram of 2-hexylidene-3-methylsuccinic acid (**3**)

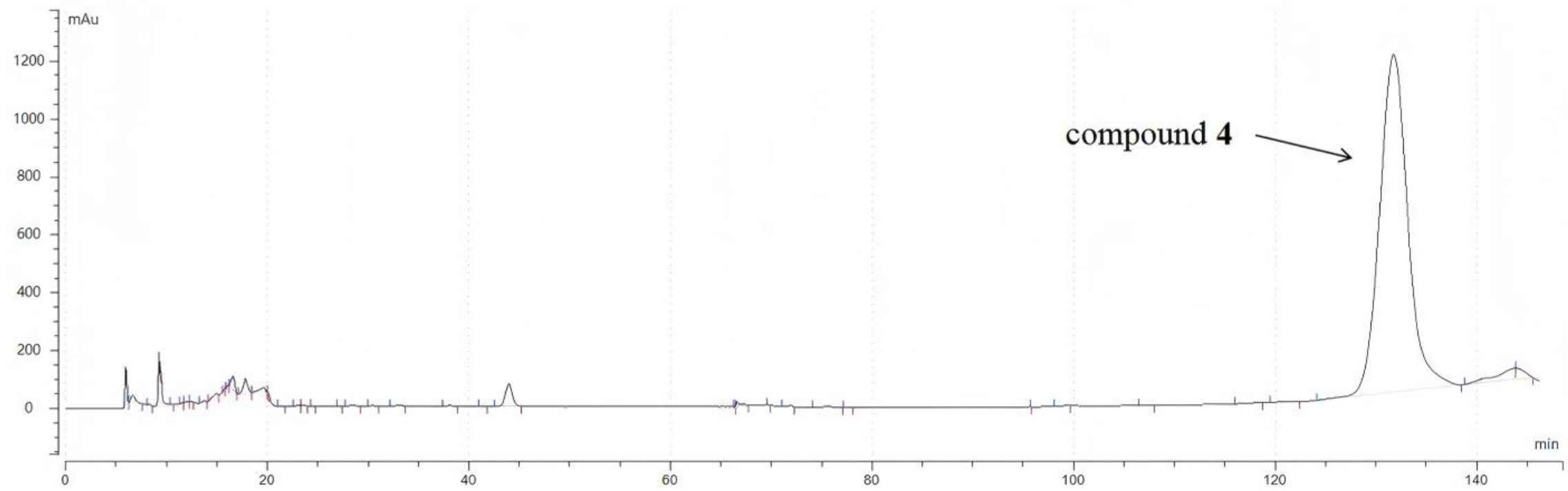

Figure S38. Preparative HPLC chromatogram of xylcarpin C (**4**)

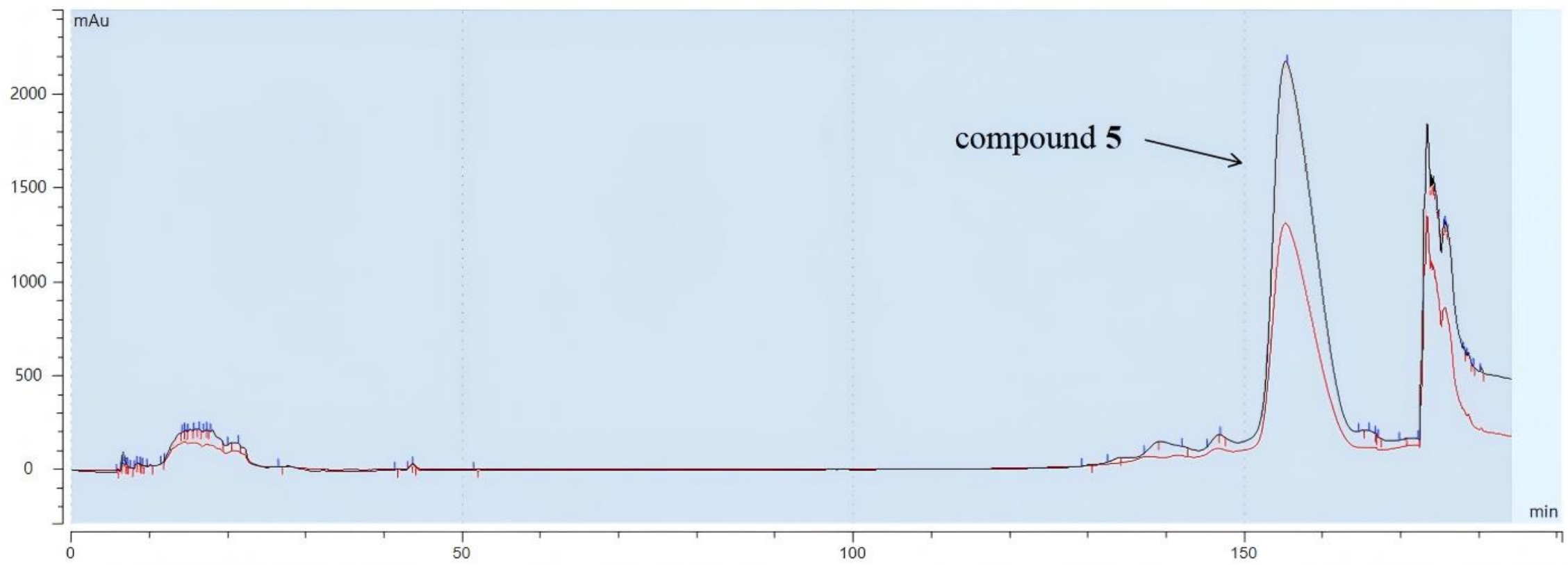

Figure S39. Preparative HPLC chromatogram of (24*R*)-22,23-dihydroxy-ergosta-4,6,8(14)-trien-3-one 23-*β*-D-glucopyranoside (**5**)

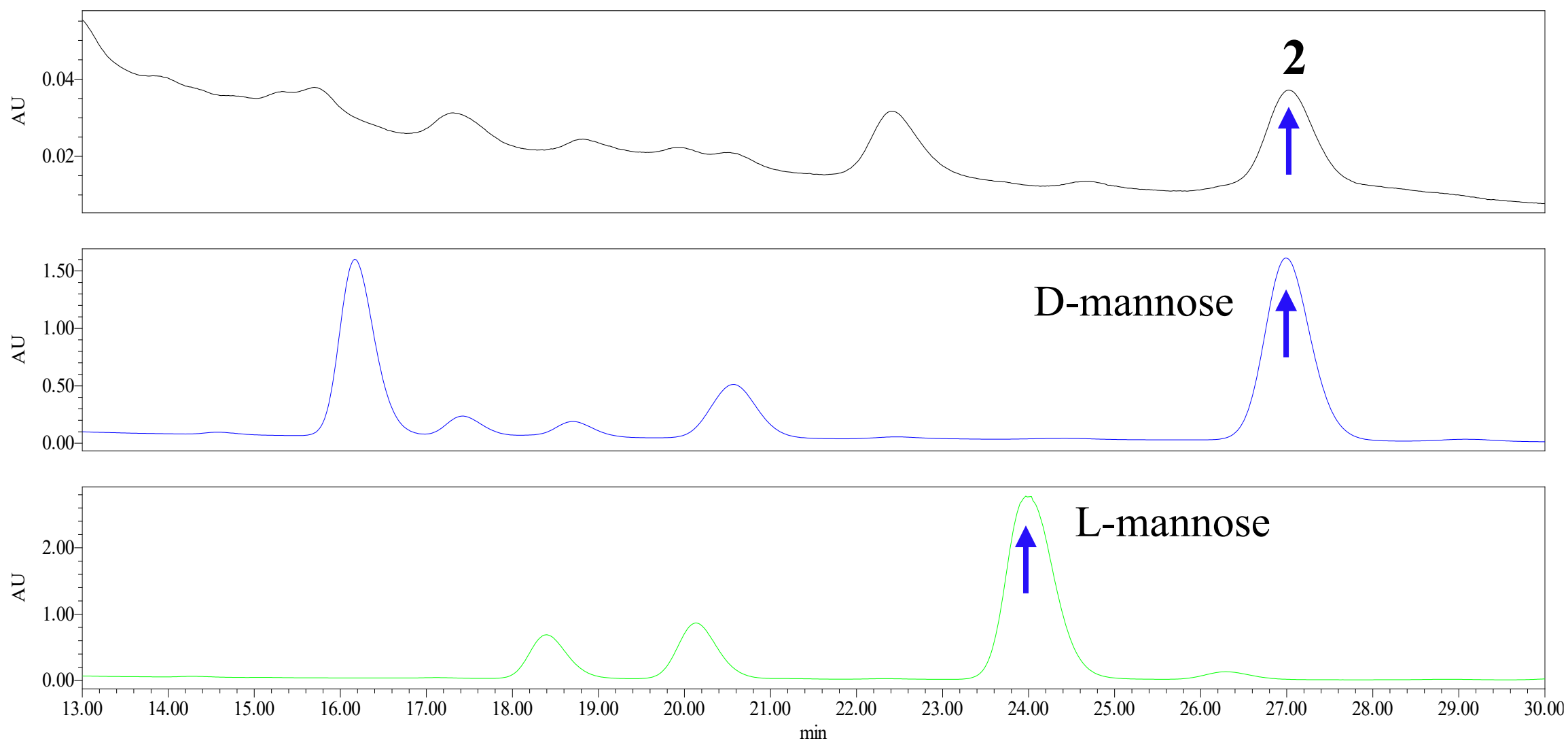

Figure S40 HPLC chromatogram for sugar derivatization of compound **2** and derivatized D-mannose and L-mannose standards
